# RMut: R package for a Boolean sensitivity analysis against various types of mutations

**DOI:** 10.1371/journal.pone.0213736

**Published:** 2019-03-19

**Authors:** Hung-Cuong Trinh, Yung-Keun Kwon

**Affiliations:** 1 Faculty of Information Technology, Ton Duc Thang University, Ho Chi Minh City, Vietnam; 2 Department of Electrical/Electronic and Computer Engineering, University of Ulsan, Nam-gu, Ulsan, Korea; Universidade Federal de Santa Maria, BRAZIL

## Abstract

There have been many *in silico* studies based on a Boolean network model to investigate network sensitivity against gene or interaction mutations. However, there are no proper tools to examine the network sensitivity against many different types of mutations, including user-defined ones. To address this issue, we developed RMut, which is an R package to analyze the Boolean network-based sensitivity by efficiently employing not only many well-known node-based and edgetic mutations but also novel user-defined mutations. In addition, RMut can specify the mutation area and the duration time for more precise analysis. RMut can be used to analyze large-scale networks because it is implemented in a parallel algorithm using the OpenCL library. In the first case study, we observed that the real biological networks were most sensitive to overexpression/state-flip and edge-addition/-reverse mutations among node-based and edgetic mutations, respectively. In the second case study, we showed that edgetic mutations can predict drug-targets better than node-based mutations. Finally, we examined the network sensitivity to double edge-removal mutations and found an interesting synergistic effect. Taken together, these findings indicate that RMut is a flexible R package to efficiently analyze network sensitivity to various types of mutations. RMut is available at https://github.com/csclab/RMut.

## Introduction

Many different types of mutations have been used to investigate dynamic behaviors of biological networks; these have focused on essential components identification [1, 2], genetic interactions prediction [3], network intervention [4], and the relationship between dynamic and structural properties [5–7]. In addition, many computational tools have been developed to support *in silico* simulations based on these mutations. For example, CABeRNET, a recent Cytoscape app, can assess the dynamics of a network via state-flip, knockout, and overexpression mutations [8]. PANET was developed for parallel analysis of sensitivity-related dynamics against state-flip and rule-flip mutations in large-scale networks [9]. BooleSim [10], Cell Collective [11], and GINsim [12] can manipulate dynamic simulations by employing knockout and overexpression mutations. GDSCalc [13] can evaluate the stability of network dynamics upon a state-flip mutation. BoolNet [14] can investigate network sensitivity via state-flip, knockout, and overexpression mutations.

However, each of these tools provides a partial set of previously well-known mutation types, most of which were designed to examine the effects of nodes on network dynamics. On the other hand, there are few tools implementing edgetic mutations, even though recent experimental results have shown that edgetic mutations are useful for genotype-to-phenotype relationship identification and drug discovery [15, 16]. Furthermore, the existing *in silico* tools are not flexible because only a few prespecified mutations can be simulated for analysis. To overcome these limitations, we developed a novel R package called RMut, which can investigate network sensitivity for many well-known node-based and edgetic mutations, as well as user-defined mutations using a synchronous Boolean network model. In addition, we can specify the mutation area and the duration time for more precise analysis. To specify the unknown regulatory rules, we employed the nested canalyzing function (NCF) model [17] where a Boolean function is constructed by randomly choosing a sequence of pairs of a canalyzing gene and a canalyzed value. The package provides some additional functions such as attractor identification, feedback/feed-forward search, and centrality calculations. To allow analysis of large-scale networks, we implemented RMut in a parallel computation using the OpenCL library. We note that the core algorithms of RMut were written in Java; thus, a Java SE Development Kit (JDK) is required to run it.

In this study, the usefulness of RMut was demonstrated through three case studies. First, we compared 10 different mutations predefined in RMut over real biological networks, and found that the networks are most sensitive to overexpression/state-flip and edge-addition/-reverse mutations among node-based and edgetic mutations, respectively. In the second case study, we further observed that edgetic mutations can predict drug-targets better than node-based mutations. Interestingly, edge-attenuation (which has never been considered in previous tools) showed high performance in drug-targets prediction. Finally, we examined the network sensitivity to double edge-removal mutations and found a synergistic effect. Altogether, these findings indicate that RMut is a useful and flexible tool for analyzing network dynamics against various types of mutations.

## Methods and implementations

This section is organized into four subsections. A Boolean network model employed in this study is first introduced. The next two subsections present predefined mutations that have been widely used in previous studies, and user-defined mutations based on a Java template implementation, respectively. Finally, two network sensitivity measures used in this study are defined.

### A Boolean network model

A Boolean network is represented by a directed graph *G*(*V*,*E*), where *V* = {*v*_1_,*v*_2_,…,*v*_*N*_} is a set of nodes, and *E* = {*e*_1_,*e*_2_,…,*e*_*M*_} is a set of ordered pairs of nodes called directed edges. The state of each node *v*∈*V* is represented by a Boolean variable having a value of 1 or 0. A directed link (*v*_*s*_,*v*_*t*_)∈*E* has a positive or negative relationship from *v*_*s*_ to *v*_*t*_ (*v*_*s*_ and *v*_*t*_ are called the source and the target nodes of the link, respectively). For a node *v*_*i*_∈*V* with *d*_*i*_ incoming links from nodes $u_{1},u_{2},\ldots ,u_{d_{i}}$ where *u*_*j*_≠*u*_*k*_ for ∀*j*≠*k*, the value of *v*_*i*_ at time *t* + 1 is determined by the values of $u_{1},u_{2},\ldots ,u_{d_{i}}$ at time *t* by a Boolean function $f_{i}:{\left\{0,1\right\}}^{d_{i}}\to \{0,1\}\;(u_{1},u_{2},\ldots ,u_{d_{i}}$ are called *input* nodes of *v*_*i*_). Here, we employed a nested canalyzing function (NCF) model [17] to represent the update rule as follows:

$$v_{i}(t+1)=f_{i}\left(u_{1}(t),u_{2}(t),\ldots ,u_{d_{i}}(t)\right)$$

$$=\left\{ \begin{array}{l} O_{1}\;if\;u_{1}(t)=I_{1}\\ O_{2}\;if\;u_{1}(t)\neq I_{1}\;\mathrm{and}\;u_{2}(t)=I_{2}\\ O_{3}\;if\;u_{1}(t)\neq I_{1}\;\mathrm{and}\;u_{2}(t)\neq I_{2}\;\mathrm{and}\;u_{3}(t)=I_{3}\\ \;\vdots \\ O_{d_{i}}\;if\;u_{1}(t)\neq I_{1}\;\mathrm{and}\;\cdots \;\mathrm{and}u_{d_{i}-1}(t)\neq I_{d_{i}-1}\;\mathrm{and}\;u_{d_{i}}(t)=I_{d_{i}}\\ O_{{default}_{i}}\;otherwise \end{array}\right.$$

where *I*_*k*_ and *O*_*k*_ (*k* = 1,2,⋯,*d*_*i*_) are called canalyzing and canalyzed Boolean values, respectively, and $O_{{default}_{i}}$ is generally set to $1-O_{d_{i}}$. For convenience, we denote *f*_*i*_ as $(I_{1},O_{1})(I_{2},O_{2})\cdots (I_{d_{i}},O_{d_{i}})O_{{default}_{i}}$, which is a sequence of pairs of canalyzing and canalyzed values, followed by the default value. In addition, *v*_*i*_(*t*+1) can be expressed in a recursive form of Boolean logic as follows:

$$v_{i}(t+1)=f_{i}\left(u_{1}(t),u_{2}(t),\ldots ,u_{d_{i}}(t)\right)={f_{i}}^{\left(d_{i}\right)}$$

$$\mathrm{where}\;{f_{i}}^{\left(d_{i}-k+1\right)}=\left\{ \begin{array}{l} u_{k}(t)\wedge (f^{\left(d_{i}-k\right)})\;if\;k<d_{i}\;\mathrm{and}\;O_{k}=0\;\mathrm{and}\;O_{k}=I_{k}\\ \bar{u_{k}(t)}\wedge (f^{\left(d_{i}-k\right)})\;if\;k<d_{i}\;\mathrm{and}\;O_{k}=0\;\mathrm{and}\;O_{k}\neq I_{k}\\ u_{k}(t)\vee (f^{\left(d_{i}-k\right)})\;if\;k<d_{i}\;\mathrm{and}\;O_{k}=1\;\mathrm{and}\;O_{k}=I_{k}\\ \bar{u_{k}(t)}\vee (f^{\left(d_{i}-k\right)})\;if\;k<d_{i}\;\mathrm{and}\;O_{k}=1\;\mathrm{and}\;O_{k}\neq I_{k}\\ u_{k}(t)\;if\;k=d_{i}\;\mathrm{and}\;O_{k}=I_{k}\\ \bar{u_{k}(t)}\;if\;k=d_{i}\;\mathrm{and}\;O_{k}\neq I_{k} \end{array}\right.$$

Herein, *k* = 1,2,⋯,*d*_*i*_, and $\bar{u(t)}$ represents the negation value of *u*(*t*) (i.e., $\bar{u(t)}=1-u(t))$. A canalyzing rule means a Boolean rule with a property such that a specific value of one of inputs alone determines the output value. This input and output values are referred to as the canalyzing and the canalyzed value, respectively. A nested canalyzing function is a recursive extension of canalyzing functions as follows. When the first canalyzing variable is not set to the canalyzing value, the second canalyzing variable and the corresponding canalyzing/canalyzed values are determined. By repeating this determination over all regulatory genes, the nested canalyzing function is constructed. It was shown that NCFs properly fit the experimental data obtained from a literature [17]. Furthermore, many logical interaction rules inferred from gene expression data can be represented by NCFs [18, 19]. As in previous studies [17, 20], we independently and randomly specified *I*_*k*_ and *O*_*k*_ values with the probabilities as:

$$Pr(I_{k}=1)=\frac{1}{2}\;and\;Pr(O_{k}=1)=\frac{exp({-2}^{-k}\theta )}{1+exp({-2}^{-k}\theta )}$$ (1)

where *θ* is a constant. In this study, *θ* was set to 7 following the previous studies [17, 20], which implies that the value of *O*_*k*_ is more likely to be biased to 0 as *k* decreases. We note that some previous tools such as BoolNet [14] and CABeRNET [8] also employed the NCF model to generate random update-rule functions.

A state of a network *G* is defined as a vector of the states of all nodes, which are synchronously updated by a set of update functions *F* = {*f*_1_,*f*_2_,…,*f*_*N*_}. A state trajectory starts from an initial state and eventually converges to either a fixed-point or a limit-cycle attractor. When another trajectory starting from the same initial state along with mutations converges to a different attractor, the network is regarded as sensitive to the mutations.

### Predefined mutations

We conducted a survey of different types of mutations in previous *in silico* studies (see Table A in S1 File for details), and found the 10 most frequently used mutations and classified them into node-based and edgetic mutation groups as follows:

- Node-based mutation group: state-flip, knockout, overexpression, rule-flip, and outcome-shuffle.
- Edgetic mutation group: edge-removal, edge-addition, edge-attenuation, edge-sign-switch, and edge-reverse.

**Fig 1:**
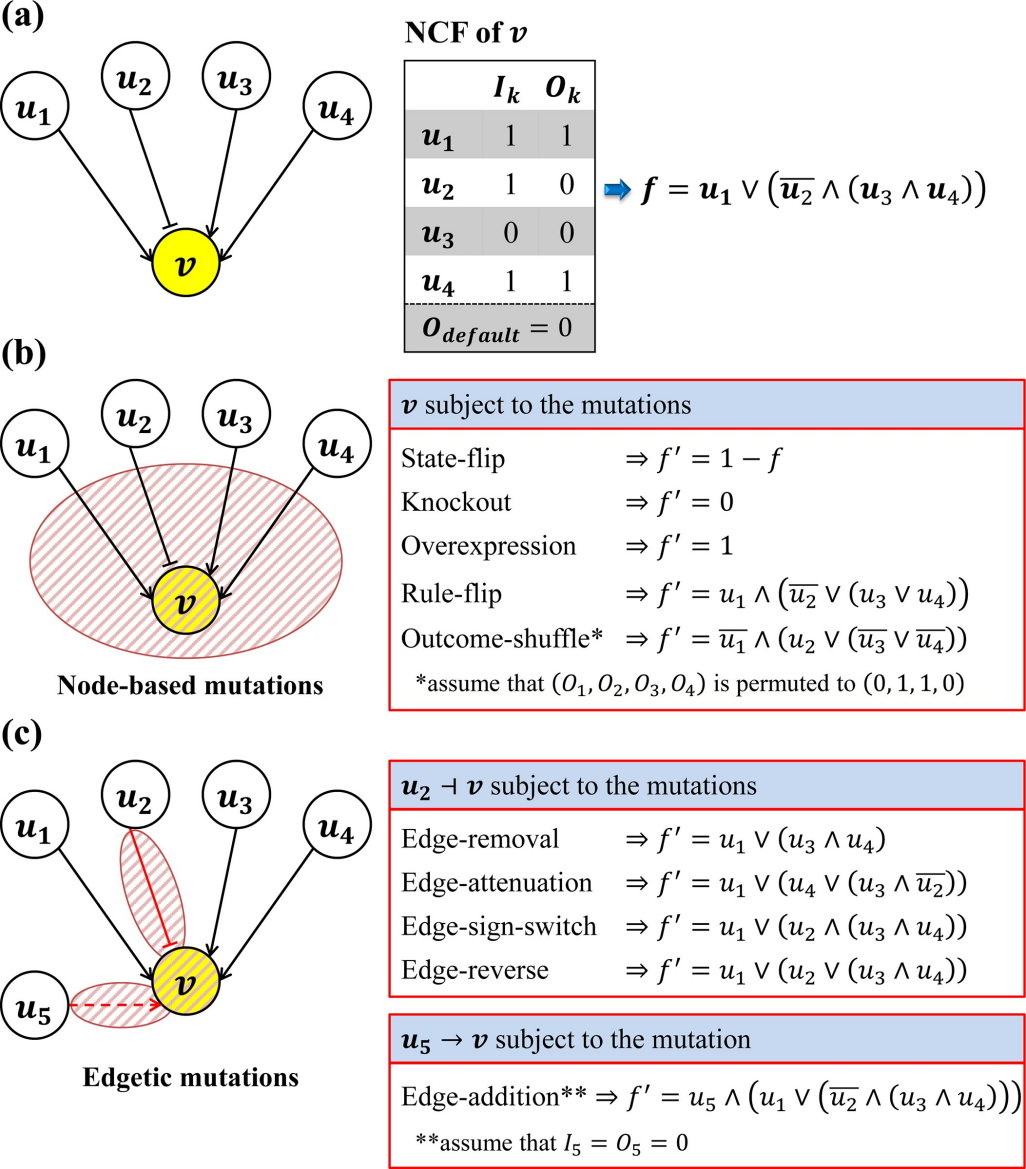
An illustrative example of the predefined mutations implemented in RMut. (a) An example node subject to mutations. Let *v* be a node with four incoming links from a set of nodes *u*_1_,*u*_2_,*u*_3_, and *u*_4_, and *f* be the update rule of *v*. The arrows and bar-headed lines represent positive and negative interactions, respectively. (b) Changes of the update function by node-based mutations subject to node *v*. The update rule *f* is modified to *f*′ by each of five node-based mutations. (c) Changes of the update function by edgetic mutations subject to (*u*_5_,*v*)∉*E* or (*u*_2_,*v*)∈*E* in the case of the edge-addition and the other edgetic mutations, respectively.

In the following subsections, we explain each mutation in detail, and each is illustrated in Fig 1. Node-based mutations represent changes to most incoming interactions, whereas edgetic mutations represent changes to specific incoming interactions.

### Node-based mutations

Let *v*_*i*_ be a node subject to a mutation. Note that each node-based mutation causes a change from *F* = {*f*_1_,…,*f*_*i*_,…,*f*_*N*_} to *F*′ = {*f*_1_,…,*f*_*i*_′,…,*f*_*N*_}.

- State-flip: This mutation represents a situation in which a protein or gene outputs an opposite state value to expectations [21–24]. More specifically, it can describe a biological process such that genes may become either activated or inhibited by external stimuli such as mutagens [25, 26] and heat stress [27].
  *Implementation*—this mutation is implemented by changing *f*_*i*_ to *f*_*i*_′ = 1−*f*_*i*_.
- Knockout: This mutation represents the effect of suppressing the expression of a gene or the pharmaceutical inhibition of secondary messenger production or kinase/phosphatase activity [28]. For example, it can be used to simulate the loss of p53 expression in p53 knockout mice in order to reveal a role for p53 in the protection of mice from spontaneous tumorigenesis [29, 30]. The knockout mutation is accomplished through a variety of techniques *in vivo* such as homologous recombination [31–34] and site-specific nucleases [35–37].
  *Implementation*—this mutation is implemented by changing *f*_*i*_ to *f*_*i*_′ = 0.
- Overexpression: This mutation represents the effect of induced gene expression [1]. Increased expression of a wild-type gene can also be disruptive to a cell, organism, or phenotypes [38]. The overexpression of HER2, MYC, REL, or AKT2 often drives a variety of human cancers [39]. In addition, the overexpression due to a gene amplification results in drug-, insecticide-, and heavy metal-resistance [40].
  *Implementation*—this mutation is implemented by changing *f*_*i*_ to *f*_*i*_′ = 1.
- Rule-flip: This mutation corresponds to a change in the relationships between nodes. The mutation might represent a deleterious change in the function of a protein or gene [41]. It has similar effects to small-scale mutations in the DNA sequence of a gene [42–46].
  *Implementation*—this mutation is implemented by changing *f*_*i*_ to *f*_*i*_′ where every canalyzing and canalyzed value is flipped (i.e., all *I*_*k*_ and *O*_*k*_ changed to 1−*I*_*k*_ and 1−*O*_*k*_, respectively).
- Outcome-shuffle: This mutation represents the abnormal and chaotic expression of a gene, and may change the function of a protein [47]. Hence, it also has similar effects to small-scale mutations in the DNA sequence of a gene [42–46].
  *Implementation*—this mutation is implemented by changing *f*_*i*_ to *f*_*i*_′ where the canalyzed values are permuted (i.e., all canalyzed values are randomly changed to $\left({O_{1}^{\prime },O_{2}^{\prime },\ldots ,O}_{d_{i}}^{\prime }\right)$, which is a permutation of $\left({O_{1},O_{2},\ldots ,O}_{d_{i}}\right)$).

### Edgetic mutations

In this study, we define five edgetic mutations. Let *v*_*i*_ be a target node of a link subject to an edgetic mutation, and $f_{i}=(I_{1},O_{1})\cdots (I_{d_{i}},O_{d_{i}})O_{{default}_{i}}$ is the update rule of *v*_*i*_. We first explain the edge-addition mutation, and let (*u*_*k*_,*v*_*i*_)∉*E* be a new edge, which will be added by a mutation.

- Edge-addition: This mutation represents the gain of a new molecular interaction between two proteins or genes [48]. In fact, it is known that some disease potentially can be led through the new incorrect interactions [49, 50]. For instance, the Glu6Val mutation of β-hemoglobin causes sickle cell anemia [51]. Specifically, this mutation introduces a hydrophobic residue to the surface of the protein that can bind to a hydrophobic patch on another hemoglobin molecule under low oxygen conditions, leading to polymerization and the characteristic sickling of the erythrocyte [52].

*Implementation*—this mutation is implemented by changing *E* to *E*′ = *E*∪(*u*_*k*_,*v*_*i*_) and *f*_*i*_ to $f_{i}^{\prime }=(I_{k},O_{k})(I_{1},O_{1})\cdots (I_{d_{i}},O_{d_{i}})O_{{default}_{i}}$. This is accomplished by inserting (*I*_*k*_,*O*_*k*_), which represents the interaction from *u*_*k*_ to *v*_*i*_, into the first position of *f*_*i*_. Note that *I*_*k*_ and *O*_*k*_ values are specified by Eq (1).

For the rest of the edgetic mutations, let (*u*_*j*_,*v*_*i*_)∈*E* be an edge subject to a mutation.

- Edge-removal: This mutation represents the loss of a specific molecular interaction between two proteins or genes [48]. For example, the cancer associated C305F missense mutation in the acidic zinc finger domain of Mdm2 results in the loss of Mdm2 binding to L5 and L11. This edgetic perturbation causes the loss of the ribosomal stress response and an increase in c-Myc induced tumorigenesis [53].

*Implementation*—this mutation is implemented by changing *E* to *E*′ = *E*\(*u*_*j*_,*v*_*i*_) and *f*_*i*_ to $f_{i}^{\prime }=(I_{1},O_{1})\cdots (I_{j-1},O_{j-1})(I_{j+1},O_{j+1})\cdots (I_{d_{i}},O_{d_{i}})O_{{default}_{i}}$ by removing (*I*_*j*_,*O*_*j*_) from *f*_*i*_.

- Edge-attenuation: This mutation corresponds to the weakening of a specific molecular interaction between two proteins or genes [54–56]. As mentioned in a previous study, low-affinity drugs inhibit their targets and can change a strong link into a weak link instead of eliminating the link completely [55].

*Implementation*—this mutation is implemented by changing *f*_*i*_ to $f_{i}^{\prime }=(I_{1},O_{1})\cdots (I_{j-1},O_{j-1})(I_{d_{i}},O_{d_{i}})(I_{j+1},O_{j+1})\cdots (I_{j},O_{j})O_{{default}_{i}}$ by swapping (*I*_*j*_,*O*_*j*_) with $\left(I_{d_{i}},O_{d_{i}}\right)$ in *f*_*i*_. Note that the position order in the sequence of the pairs of canalyzing and canalyzed values represents the order of precedence in updating the state value of *v*_*i*_.

- Edge-sign-switch: This mutation represents the switch of the type of molecular interaction between two proteins or genes [57]. An activating interaction is switched to an inhibiting one, and vice versa. This mutation represents the partial change in the function of the source gene/protein as other types of edgetic mutations, but no experimental study about this mutation has been reported.

*Implementation*—this mutation is implemented by changing *f*_*i*_ to $f_{i}^{\prime }=(I_{1},O_{1})\cdots ({1-I}_{j},O_{j})\cdots (I_{d_{i}},O_{d_{i}})O_{{default}_{i}}$.

- Edge-reverse: This mutation represents a switch in both the type and outcome of a molecular interaction or relationship between two proteins or genes [58]. In other words, both the activation/inhibition relationship and the output value of an interaction are switched. As in the edge-sign-switch mutation, there is no experimental study about this mutation yet.

*Implementation*—this mutation is implemented by changing *f*_*i*_ to $f_{i}^{\prime }=(I_{1},O_{1})\cdots (I_{j},1-O_{j})\cdots (I_{d_{i}},O_{d_{i}})O_{{default}_{i}}$.

These previous mutations were predefined in RMut and therefore can be easily embedded by setting a parameter value. We note that there was no previous tool implementing all of these mutations, and Table 1 shows a comparison of RMut and other tools with respect to available types of mutations.

**Table 1:** Comparisons of RMut and other *in silico* tools based on available mutation types.

Mutation type	RMut	BoolNet	CABeRNET	PANET	Cell-Collective	BooleSim	GINsim	GDSCalc
State-flip	✓	✓	✓	✓				✓
Rule-flip	✓			✓				
Knockout & Overexpression	✓	✓	✓		✓	✓	✓	
Outcome-shuffle	✓	✓						
Edge-attenuation	✓							
Edge-removal	✓							
Edge-addition	✓							
Edge-sign-switch	✓							
Edge-reverse	✓	✓						

### User-defined mutations

**Fig 2:**
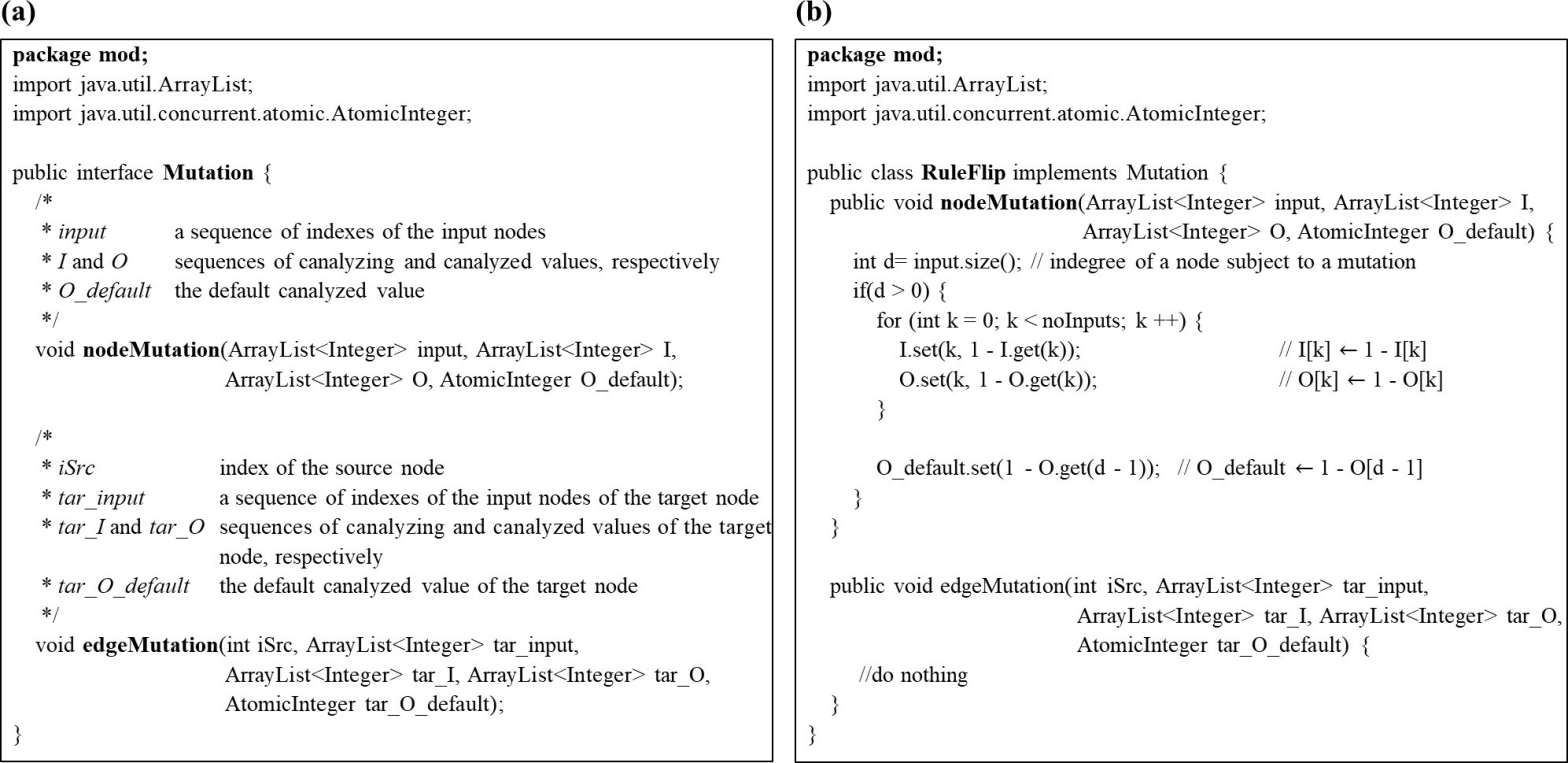
User-defined mutations in RMut. (a) A Java template for implementation of a user-defined mutation. (b) An example of reimplementing the rule-flip mutation using the template.

Although most well-known mutations are included in RMut, it is also interesting to simulate new kinds of mutations because some future experimental studies can discover a new mutation type. For example, the edgetic mutations have emerged as a promising strategy for interpretation of genotype-to-phenotype relationships in recent years [50]. To this end, we created a Java template in which a user can flexibly implement novel mutations (Fig 2A). Specifically, RMut provides two functions for node-based and edgetic mutations, respectively. Therefore, many different types of node-based, edgetic mutations, or both can be embedded in RMut. Fig 2B shows an example of an implementation wherein the rule-flip mutation is redefined (see Figures A-I in S1 File for other mutations).

**Fig 3:**
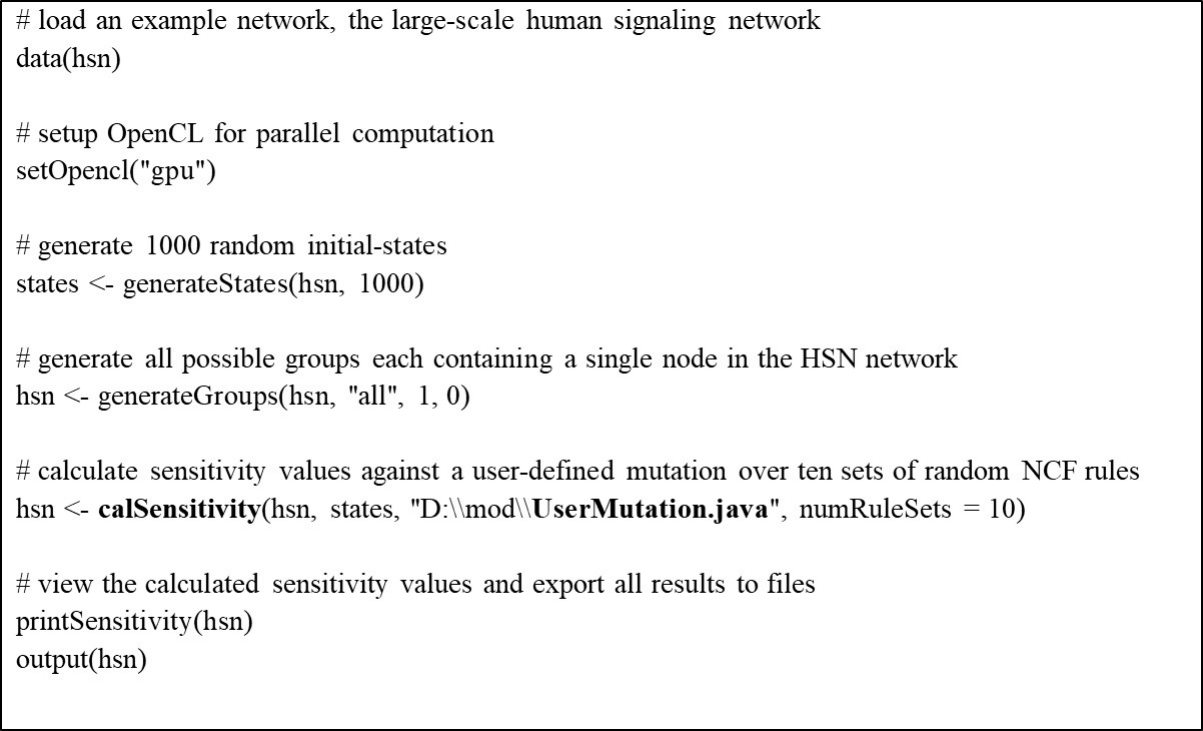
An example of network sensitivity analysis using RMut.

Although it is possible to implement user-defined mutations in some previous tools like BoolNet [14], we note that RMut provides a more systematic way to employ them for the dynamics analysis by using the Java template function. Fig 3 shows an example code for dynamics analysis using a user-defined mutation. As shown in the figure, a user can simply simulate a user-defined mutation, which was saved to a java file, by specifying the file path when calling the sensitivity calculation function ‘calSensitivity()’.

### Sensitivity analysis based on a Boolean network model

As described before, we employed a Boolean network model to investigate the network sensitivity. Given a Boolean network, a network state trajectory starting from an initial state converges to an attractor. When another trajectory starting from the same initial state along with a mutation converges to a different attractor, the network is called sensitive to the mutation. To quantify the network sensitivity, we define a mutation group consisting of a subset of nodes, a subset of edges, or both, which describes the area subject to the mutation. This notion allows RMut to use multiple mutations or a single mutation for analysis. We further define the duration time of a mutation, denoted by *τ*. It means that a mutation is assumed to occur only for a time period *t*∈[1,*τ*]. We note that no previous tools incorporated either mutation area or duration time. Given a Boolean network *G*(*V*,*E*), a *network state* at time *t* can be denoted by an ordered list of state values of all nodes, **v**(*t*) = [*v*_1_(*t*),*v*_2_(*t*),…,*v*_*N*_(*t*)]∈{0,1}^*N*^. Every network state transits to another network state through a set of Boolean update functions *F* = {*f*_1_,*f*_2_,…,*f*_*N*_}. We firstly define an attractor more rigorously as follows:

Let **v**(0),**v**(1),⋯, be a network state trajectory starting at **v**(0). The attractor is defined as an ordered list of network states 〈*G*,*F*,**v**(0)〉 = [**v**(*τ*),**v**(*τ*+1),…,**v**(*τ*+*p*−1)] where *τ* is the smallest time step such that **v**(*t*) = **v**(*t*+*p*) for ∀*t*≥*τ* with **v**(*i*)≠**v**(*j*) for ∀*i*≠*j*∈{*τ*,*τ*+1,…,*τ*+*p*−1} (herein, *p* is called a length of the attractor).

Generate a set of random initial states *S*. For each initial state **v**(0)∈*S*, we obtain two attractors 〈*G*,*F*,**v**(0)〉 and 〈*G*′,*F*′,**v**(0)〉 in the wild-type and the mutant networks, respectively. For convenience, let 〈*G*,*F*,**v**(0)〉 = [**v**(*τ*),**v**(*τ*+1),…,**v**(*τ*+*p*−1)] and 〈*G*′,*F*′,**v**(0)〉 = [**v**′(*τ*′),**v**′(*τ*′+1),…,**v**′(*τ*′+*p*′−1)]. Finally, we define the network sensitivity as follows:

$$\lambda =\frac{\sum_{v(0)\in S}d(\langle G,F,v(0)\rangle ,\langle G^{\prime },F^{\prime },v(0)\rangle )}{\left|S\right|},$$ (2)

where *d*(∙) denotes a distance function between the wild-type and the mutant attractors. Specifically, we considered two different distance functions based on identicalness and similarity of attractors, respectively, in this study. Let *H*(**v**(*t*),**v**′(*t*′)) the Hamming distance between a pair of Boolean vectors, **v**(*t*) and **v**′(*t*′), computed as $\sum_{i=1}^{N}\left|v_{i}(t)-v_{i}^{\prime }(t^{\prime })\right|$, i.e., the number of different bits. Then the identicalness-based distance is defined as follows:

$$d(\langle G,F,v(0)\rangle ,\langle G^{\prime },F^{\prime },v(0)\rangle )=\left\{ \begin{array}{l} 1,\;p=p^{\prime }and\;\exists m\in \{0,\ldots ,p-1\}\;s.t.\sum_{l=0}^{p-1}H\left(v(\tau +l+m),v^{\prime }(\tau^{\prime }+l)\right)=0\\ 0,\;otherwise \end{array}\right..$$

In addition, the similarity-based distance function is defined considering various possible time lags between two attractors as follows:

$$d(\langle G,F,v(0)\rangle ,\langle G^{\prime },F^{\prime },v(0)\rangle )=\underset{m\in \{0,\ldots ,d-1)}{\min}\frac{1}{c∙N}\sum_{l=0}^{c-1}H(v(\tau +l+m),v^{\prime }(\tau^{\prime }+l)),$$

where *c* and *d* are the least common multiple and the greatest common divisor, respectively, of *p* and *p*′. Note that *m* in both two distance definitions represents a possible time lag between two attractors. As a result, the identicalness-based distance represents whether the wild-type and the mutant attractors are identical to each other or not whereas the similarity-based distance represents the minimum ratio of a bitwise difference between the states sequence in the wild-type and the mutant attractors over the least common period (*c*) of the two attractors.

With respect to the update function implementation, a user can choose a user-defined NCF or a randomly-generated NCF for each gene, when calling the sensitivity calculation function ‘calSensitivity()’. In the case of the user-defined NCF, a parameter of the file path containing the NCF rules should be specified (see Figure J in S1 File for example). In the case of the random NCF, the sensitivity can be averaged out over a given number of trials which is specified by a parameter ‘numRuleSets’ as shown in Fig 3.

## Parallel computation

To allow analysis of large-scale networks, the sensitivity is calculated in parallel using the OpenCL library as in our previous tool–PANET [9]. Specifically, we assign each initial state included in a set of random initial states *S* in Eq (2) to processing elements of a central processing unit or graphics processing unit where the wild-type and the mutant attractors are computed in parallel.

### Availability

RMut is OS-independent and available at https://github.com/csclab/RMut. It requires R 3.5.0 or higher, Java 8 platform (Java SE 8u202 or higher) or Java 11 platform (Java SE 11.0.2 or higher), and OpenCL library (optional). See S2 File for detailed specification of all functions and S3 File for user-manual.

All data generated or analyzed during this study are included in this published project and its supplementary information files.

## Case studies

To demonstrate the usefulness of RMut, we conducted three case studies using the following real biological networks.

- HSN: This is the large-scale human signaling network (HSN) with 1192 nodes and 3102 links after removing neutral links [59]. Based on the network, some general principles were provided for understanding protein evolution in the context of signaling networks.
- CCSN: This is the canonical cell signaling network (CCSN) with 771 nodes and 1633 links [60]. The network was obtained from http://stke.sciencemag.org/, and all the neutral interactions were excluded.
- AMRN: This is the *Arabidopsis* morphogenesis regulatory network (AMRN) with 10 nodes and 22 links [61]. This regulatory network is known to robustly control the process of flower development.

### Effects of different types of mutations on network sensitivity

**Fig 4:**
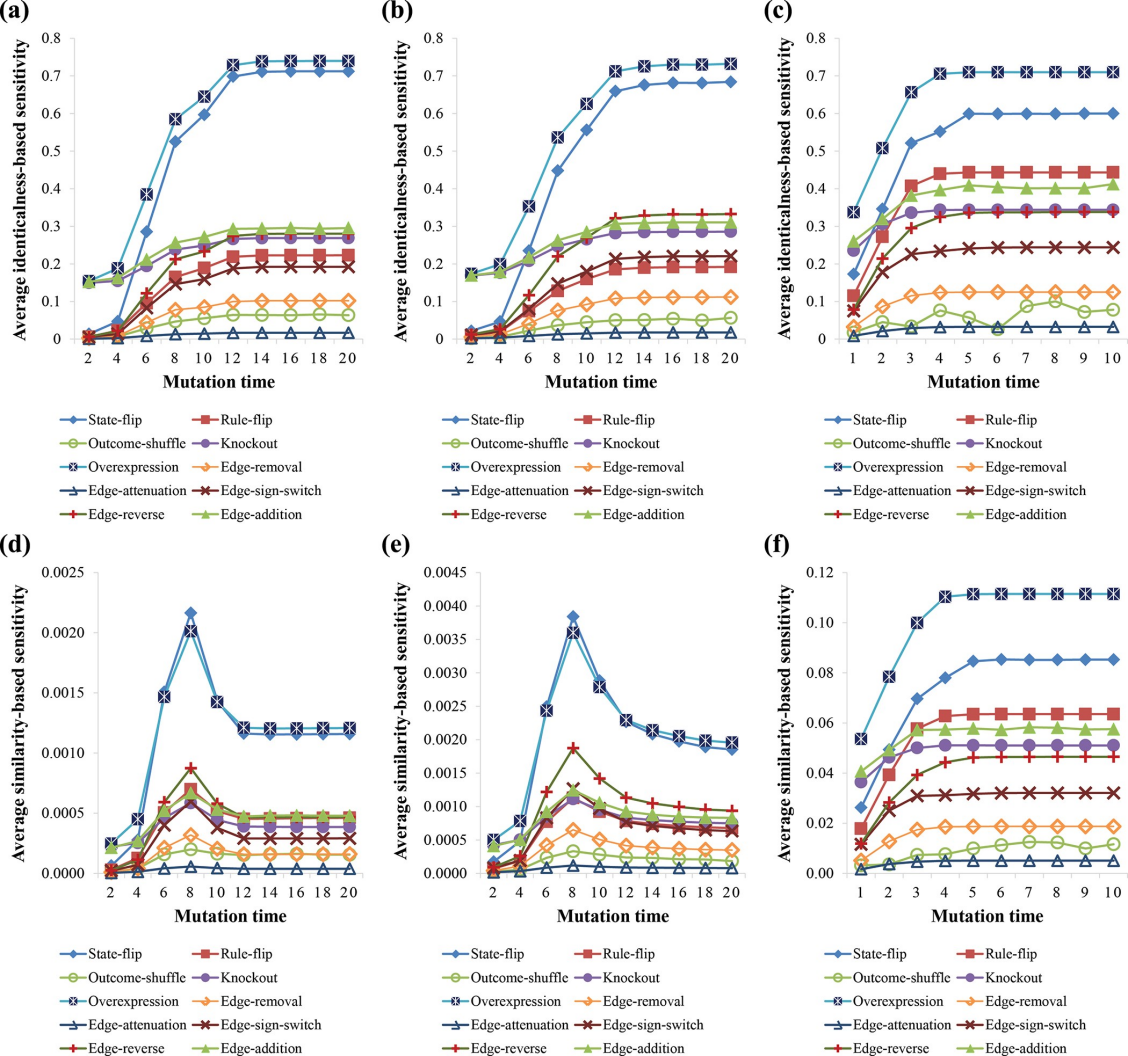
Average sensitivities based on the predefined mutations. (a)-(c) Results of HSN, CCSN, and AMRN networks, respectively, using the identicalness-based sensitivity. (d)-(f) Results of HSN, CCSN, and AMRN networks, respectively, using the similarity-based sensitivity. In each subfigure, Y-axis values represent the average sensitivity values.

In this case study, we examined the network sensitivity of 10 predefined mutations of RMut. We calculated the average sensitivity value over all nodes or edges in HSN, CCSN, and AMRN assuming that a single mutation occurs at a node (in the case of node-based mutations) or an edge (in the case of edgetic mutations) (Fig 4). The mutation duration time *τ* was varied from 1 to 10 for the small-scale network, AMRN, and from 2 to 20 by 2 for the large-scale networks, CCSN and HSN.

We note that the sensitivity was averaged over ten sets of random NCF rules. It took about 8.35 days, 3.55 days, and 1.26 minutes to conduct a total of 1000 simulations (10 mutations × 10 mutation times × 10 sets of NCF rules) for HSN, CCSN, and AMRN networks, respectively, on a system with an NVIDIA GeForce GTX 680 GPU with 1536 processor at 1 GHz, four-core Intel Core i7-3770 CPU 3.40 GHz, and 8 GB of memory. As shown in Fig 4A–4C, the sensitivity values based on the attractor-identicalness distance increased as the mutation time increased. The sensitivity values based on the attractor-similarity distance also increased against the mutation time on the small-scale network (Fig 4F) but it decreased after some mutation time (*τ* = 8) for the large-scale networks (Fig 4D and 4E). It turns out that the sensitivity value of some mutations was considerably variable against the mutation time. In addition, we found that the overexpression mutations showed the largest average sensitivity values in all real networks. On the other hand, the knockout mutation showed smaller sensitivity values than the overexpression mutation because the output values of a Boolean variable are likely to be biased to 0 (see Eq (1)). The state-flip mutation showed the second largest sensitivity values, while the rule-flip showed a moderate level of sensitivity. These node-based mutations have been employed in a variety of studies, but without comparing them with other types of mutations. For instance, knockout and overexpression mutations [1, 28], or rule-flip and state-flip mutations [2, 22] were used to predict essential components in signaling networks. Another study used the knockout mutation to predict mutant phenotypes of fission yeast [62]. Shmulevich, Dougherty [21] developed a method for intervening dynamical network behaviors using the state-flip mutation. Moreover, the relationships between dynamic behaviors and structural properties were examined based on knockout and rule-flip mutations [5–7]. These previous studies can be extended by employing additional mutations, considering that some recent studies [63–66] have found pleiotropic phenomenon such that different types of mutations can occur in a same gene. Finally, the outcome-shuffle mutation having shown the smallest sensitivity value among the node-based mutations was rarely investigated [14, 47].

Edgetic mutations have been recently considered in experimental studies to better reveal genotype-to-phenotype relationships and drug discovery [15, 16]. Some *in silico* studies have also been conducted [57, 58, 67]. For example, Li, Long [57] investigated the dynamic properties and stability of the yeast cell-cycle network by applying edge-removal, edge-addition, and edge-sign-switch mutations. In this study, we examined a total of five edgetic mutations including all of the previous ones. As shown in Fig 4, we observed that the edge-addition mutations showed much larger sensitivity values than the edge-removal mutations in all real networks. This is interesting because these two mutations seem to be similar to each other in terms of the number of changed interactions. It is known that the edge-addition mutation can lead to disease by incorporating new incorrect interactions [68–70], or can also prevent unexpected network damage by recovering the loss of other interactions [67]. This implies that the edge-addition mutation can be an efficient tool to control the network dynamics, although it is costly and difficult to implement in experimental studies [71]. In addition, edge-reverse mutations showed larger sensitivity values than the edge-sign-switch mutations, which is expected based on the definitions. Previous studies have focused on the former [4, 14, 47, 58, 72] rather than the latter [57]. Specifically, edge-reverse mutations were implemented by flipping an output value of an input variable in a Boolean update rule [4, 58]. Finally, the edge-attenuation mutation, which was not considered in any previous *in silico* studies, showed the smallest sensitivity values in all real networks.

### Comparisons of node-based and edgetic sensitivities in drug-targets prediction

**Fig 5:**
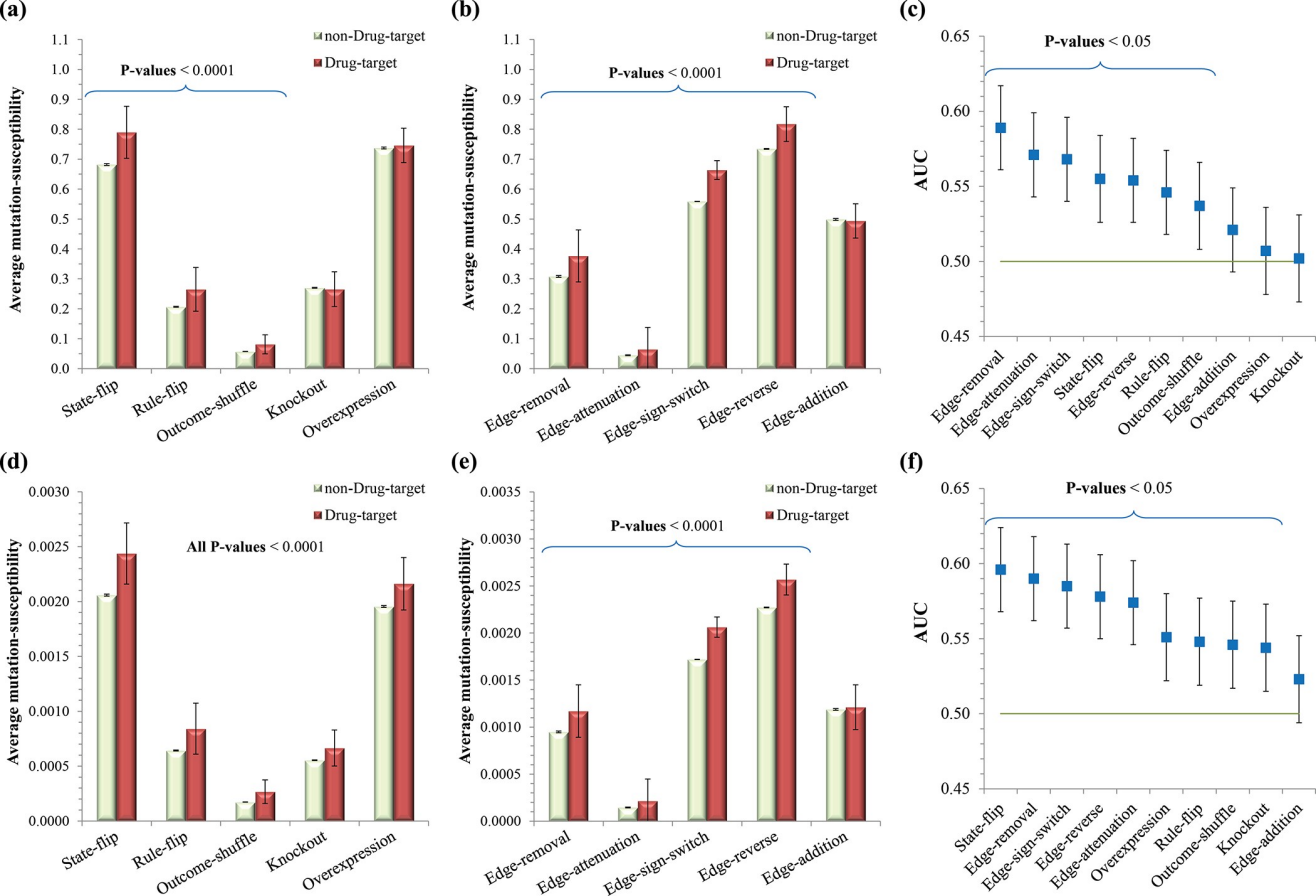
Results of drug-targets prediction based on network sensitivity analysis using RMut. (a)-(b) Comparison of average mutation-susceptibility values between groups of drug-targets and non-drug-targets over node-based and edgetic mutations, respectively, using the identicalness-based sensitivity. (c) AUC values in drug-target prediction using the identicalness-based sensitivity. (d)-(e) Comparison of average mutation-susceptibility values between groups of drug-targets and non-drug-targets over node-based and edgetic mutations, respectively, using the similarity-based sensitivity. (f) AUC values in drug-target prediction using the similarity-based sensitivity. In all sub-figures, the error bars represent 95% confidence intervals.

To show the usefulness of RMut, we applied it to the drug-target identification problem. A drug-target is a protein, peptide or nucleic acid whose activity can be modulated by a drug to produce a specific effect, which might be a desirable therapeutic effect or an unwanted adverse effect. We hypothesized that a network is susceptible to mutations subject to drug-target genes. To validate this hypothesis, we first profiled a total of 333 drug targets included in HSN using the DrugBank database [73]. We defined the mutation-susceptibility of a node to represent how susceptible a network is to a mutation subject to that node as follows. For a node *v* and an edge *e*, let *λ*(*v*) and *λ*(*e*) be the network sensitivity value when the mutation group consists only of *v* and *e*, respectively. In the case of node-based mutations, the mutation-susceptibility of node *v* corresponds to *λ*(*v*), whereas in the case of edgetic mutations, it is specified by max_*e*∈*A*_*λ*(*e*), where *A* is the set of edges that are incident to *v*. The mutation duration time *τ* was set to 20 and 8 for identicalness- and similarity-based sensitivities, respectively, because they led to the highest sensitivity to mutations (Fig 4A and 4D). We compared the average mutation-susceptibility values between groups of drug-targets and non-drug targets in HSN (Fig 5A, 5B, 5D and 5E). As shown in the figure, the average mutation-susceptibility values of the drug-target group are significantly higher than those of the non-drug target group (P-values < 0.0001 using t-test) in three or all node-based mutations (Fig 5A and 5D) for identicalness-/similarity-based sensitivity measures, respectively, and in four edgetic mutations for both identicalness-/similarity-based sensitivity measures (Fig 5B and 5E). This implies that edgetic mutations are more stable than node-based mutations in identifying drug targets. For a more precise analysis, we further examined the area under the curve (AUC) when the mutation-susceptibility value was used to prioritize candidate drug-target genes in HSN (Fig 5C and 5F). As shown in the figure, four edgetic mutations, edge-removal, edge-attenuation, edge-sign-switch, and edge-reverse are placed in the top 5 AUC values for both identicalness-/similarity-based sensitivity measures. All of these results illustrate the importance of dynamics analysis based on edgetic mutations, which have not been a primary focus in previous studies compared to node-based mutations.

### Synergy effects of double mutant interactions

In genetic interactions, synergy occurs when the contribution of two mutations to the phenotype of a double mutant exceeds the expectations from the additive effects of the individual mutations [74]. Most previous studies have focused on synergy effects based on node-based mutations such as knockout and overexpression mutations [3, 75–78], because they tried to determine the functional roles of a gene or a protein. On the other hand, no previous experimental study focused on the synergistic effects of edgetic mutations. Considering that many experimental results showed the usefulness of edgetic mutations in genotype-to-phenotype relationships and drug discovery [15, 16], *in silico* studies focusing on this issue would be interesting. Therefore, we conducted a case study to examine the synergy effects based on the edge-removal mutation using RMut. To quantify the synergy effect, we defined the deviation in network sensitivity value by a double edge-removal mutation from the expected one as a single edge-removal mutation as follows:

$$\varepsilon (e_{i},e_{j})=\lambda (e_{i}\mathrm{and}e_{j})-\psi \left(\lambda (e_{i}),\lambda (e_{j})\right),$$

where *λ*(*e*_*i*_ and *e*_*j*_) and *λ*(*e*) represent the network sensitivity values (see Eq (2) in Section 2.4) when the mutations group is {*e*_*i*_,*e*_*j*_} (i.e., a double mutation) and {*e*} (i.e., a single mutation), respectively. In addition, *ψ*(*x*,*y*) represents the expected network sensitivity to a single mutation, and the following three functions were considered for this purpose.

$$\psi_{MAX}(x,y)=max(x,y)\;(\max)$$

$$\psi_{ROOT}(x,y)=\sqrt{x^{2}+y^{2}}\;(\mathrm{square}\;\mathrm{root})$$

$$\psi_{ADD}(x,y)=min(x+y,1)\;(\mathrm{additive})$$

Note that *ψ*_*MAX*_≤*ψ*_*ROOT*_≤*ψ*_*ADD*_ holds. We examined the synergistic effects between all pairs of edges in AMRN and CCSN (Table 2), and we classified (*e*_*i*_,*e*_*j*_) into “Synergy” or “No synergy” groups if *ε*(*e*_*i*_,*e*_*j*_) is greater than a threshold *β* or not, respectively. The thresholds *β* were set to 0.1 and 0.01 in the cases of identicalness-based sensitivity (Table 2A) and similarity-based sensitivity (Table 2B), respectively. The mutation duration times were set to 10 for both identicalness-/similarity-based sensitivity measures in the case of AMRN network. For CCSN network, the mutation duration times were set to 20 and 8 for identicalness-based sensitivity and similarity-based sensitivity, respectively. These mutation times induced the highest sensitivity to single edge-removal mutations (Fig 4B, 4C, 4E and 4F). As shown in the table, the numbers of synergistic gene pairs were largest and smallest in the case of *ψ*_*MAX*_ and *ψ*_*ADD*_, respectively, due to the definitions. In addition, the percentages of the synergistic groups in CCSN were lower than those in AMRN regardless of the type of *ψ*. This implies that a pair of edges in a large network is less likely to show a synergistic effect than that in a small network. This is because the downstream areas affected by the mutations are not likely to be overlapped in a large network.

**Table 2:** Synergistic effects of double edge-removal mutations in two real biological networks, AMRN and CCSN.

**(a) Sensitivity based on attractor-identicalness**				
**AMRN**	Synergy		No synergy	
*ψ*(*x*,*y*)	Number of edge pairs	%	Number of edge pairs	%
*ψ*_*MAX*_	16	6.93	215	93.07
*ψ*_*ROOT*_	6	2.60	225	97.40
*ψ*_*ADD*_	5	2.16	226	97.84
**CCSN**	Synergy		No synergy	
*ψ*(*x*,*y*)	Number of edge pairs	%	Number of edge pairs	%
*ψ*_*MAX*_	57782	4.34	1274746	95.66
*ψ*_*ROOT*_	10426	0.78	1322102	99.22
*ψ*_*ADD*_	2759	0.21	1329769	99.79
**(b) Sensitivity based on attractor-similarity**				
**AMRN**	Synergy		No synergy	
*ψ*(*x*,*y*)	Number of edge pairs	%	Number of edge pairs	%
*ψ*_*MAX*_	52	22.51	179	77.49
*Ψ*_*ROOT*_	30	12.99	201	87.01
*ψ*_*ADD*_	7	3.03	224	96.97
			**s**	
**CCSN**	Synergy		No synergy	
*ψ*(*x*,*y*)	Number of edge pairs	%	Number of edge pairs	%
*ψ*_*MAX*_	3255	0.2443	1329273	99.7557
*ψ*_*ROOT*_	3253	0.2441	1329275	99.7559
*ψ*_*ADD*_	3249	0.2438	1329279	99.7562

In the case of *ψ*_*ADD*_, we further examined the number of edge pairs in the synergy group when both *λ*(*e*_*i*_) and *λ*(*e*_*j*_) were nonzero. This condition reflects the situation in which it is most difficult to induce a synergistic effect. Interestingly, we observed a large number of such gene pairs. Specifically, the numbers were 5 out of 5 in AMRN and 242 out of 2759 in CCSN for the identicalness-based sensitivity, and 7 out of 7 in AMRN and 1474 out of 3249 in CCSN for the similarity-based sensitivity.

These edge-pairs tend to maximize the network sensitivity in a synergistic context and could be potential candidates for future experimental studies.

### Scalability by parallel computation in RMut

**Fig 6:**
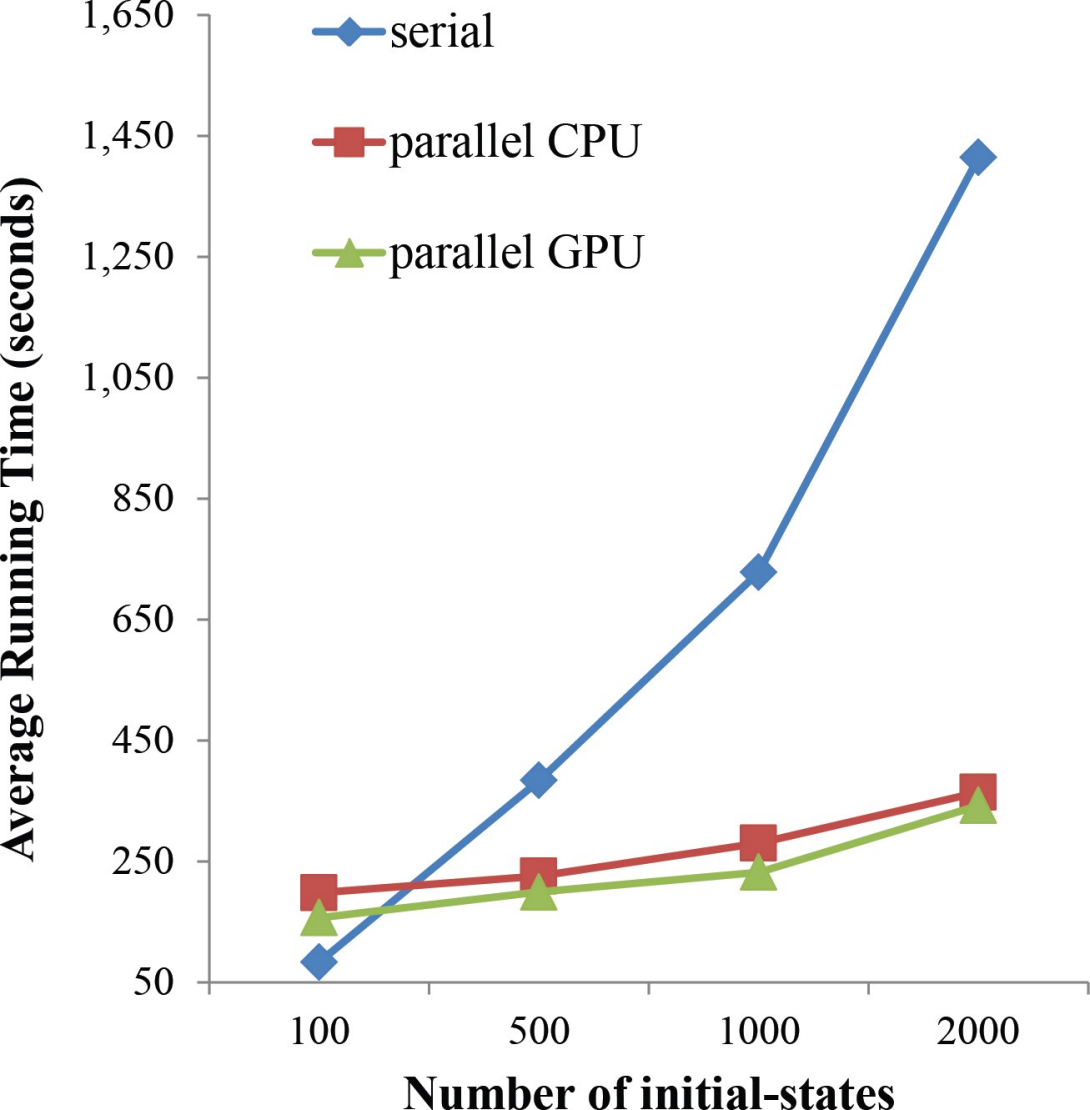
Scalability by parallel computation in RMut. We compared the running times for calculating average sensitivity of the HSN in three modes: serial computation, parallel computation on multi-core CPU (denoted as “parallel CPU”), and parallel computation on GPU (denoted as “parallel GPU”). The knockout mutation is considered and the number of initial states varied from 100 to 2000.

As we mentioned, we implemented RMut in a parallel computation using the OpenCL library. To show the scalability of it, we compared the running times of three versions such as serial, parallel on multi-core CPU, and parallel on GPU modes. We calculated the average sensitivity of the HSN and assumed the knockout mutation. All were tested on a system with an NVIDIA GeForce GTX 680 GPU with 1536 processor at 1 GHz, four-core Intel Core i7-3770 CPU 3.40 GHz, and 8 GB of memory. By varying the number of initial states, we controlled the problem size and Fig 6 shows the result. In the figure, “parallel CPU” and “parallel GPU” represent the results of parallel versions on multi-core CPU and GPU, respectively. As the number of initial-states increases, the speedup by parallelism increases. When the number of initial states was set to 100, the parallel versions were even slower than the serial version. On the other hand, the former versions were about four times faster than the latter. This result explains that the parallel implementation of RMut is properly scalable to the problem size.

## Discussion

Although it is ideal to specify all the update rule based on real regulatory relations, most of them are not available, particularly in the case of large-scale biological networks. In this regard, we employed NCFs to randomly specify the update rule in this study. It is known that NCFs can represent various types of regulatory interactions [18, 19, 79–81]. For example, 133 out of 139 rules compiled from a dataset about a transcriptional regulatory network [18] and 39 out of 42 rules inferred from a dataset about signaling pathways [19] were NCFs. Despite these supports, we note that the accurate representation of the regulatory interaction can be limited in our tool. Another issue to be discussed about our Boolean network model is the synchronous update scheme. In fact, it is very likely that the genes in the real signaling networks are regulated in an asynchronous manner. However, it is required to properly specify some unknown parameters to implement the asynchronous scheme such as the number of genes to be updated in a single step and a strategy to choose an update sequence. To avoid this problem, we employed the synchronous update scheme which can be another limitation of our tool.

Next, we discuss the applications of edgetic mutations and the related biological experiments. Despite the experimental cost and the difficulty of implementing the edgetic mutations [82, 83], many recent experimental studies have reported that the edgetic mutations are useful to identify novel genotype-to-phenotype relations [15, 49, 50, 69, 84, 85]. In particular, they mostly employed the edge-removal and the edge-addition mutations. For example, a previous study has shown how much proportion of human diseases may potentially arise from protein–protein interactions-disruptive mutations by using protein structural information [85]. Another study explained that certain PHOX2B variants are associated with neuroblastoma pathogenesis because of their inability to bind to key interacting proteins such as HPCAL1 [84]. It was also shown that the gain of interaction (edge-addition) mutation can lead to a disease by incorporating new incorrect interactions [68–70]. In addition, interaction-targeted drugs called edgetic drugs have been emerged as a novel strategy in the drug discovery [16, 86–89] because they can be more specific than node-targeted ones. Some previous studies focused on small drug-like molecules mediating protein-protein interactions [86–89], and identified a few small molecules through experiments [90–94]. In [90, 91], the authors revealed that Mdm2/Mdm-X interaction is a promising target for therapeutic reactivation of the tumor-suppressor gene p53 in cancer treatments and found small molecule inhibitors to disrupt Mdm2/Mdm-X interaction and activate p53 function. Another study [92] identified ICG-001 which is a small molecule that inhibits the interaction between β-catenin and CBP, and reduces the growth of colon carcinoma cells. On the other hand, there were few experimental studies based on other edgetic mutation types. For example, a previous study found that low-affinity drugs inhibit their targets and can change the intensity of an interaction instead of eliminating the link completely [55]. We note that this experiment can be simulated by using the edge-attenuation mutation in our tool. Taken together, a variety of edgetic mutations can be prominent in future drug discovery.

Finally, we here summarize the benefits of RMut package over the previous existing tools. The most benefit is that a user can analyze the network dynamics over many different types of mutations as well as novel user-defined ones. In addition, it is possible to more precisely analyze the dynamics by changing various environmental parameters such as the mutation area (i.e., multiple mutations) and the duration time. Moreover, the large-scale networks can be investigated due to the parallel implementation using an OpenCL platform. Our package also features not only the dynamics analysis but also the structure analysis such as calculating node-/edge-based centralities and identifying feedback/feed-forward loops in a single package. Based on these advantages, RMut package can be used in various applications. For example, we can identify some essential components [1, 2] by examining the sensitivity values of the interested components. In addition, it can be used to predict genetic interactions [3] by comparing the sensitivity value of a double gene mutation from the value predicted from single mutations, and reveal the network intervention [4] by applying the state-flip mutation subject to a single gene. It is also possible to investigate an emergent property by examining the relationship between dynamic and structural properties [5–7]. Another application is the drug discovery [15, 16] by computing the sensitivity values of the genes or interactions to identify drug-target candidates. In this way, we believe our tool can give various benefits to many researchers.

## Conclusions

We developed RMut, which is an efficient R package to investigate the network sensitivity for both predefined node-based and edgetic mutations. Moreover, new user-defined mutations can be easily embedded using a Java template. RMut also provides more precise analysis by specifying the mutation area and the duration time. We implemented RMut in a parallel algorithm using the OpenCL library to analyze large-scale networks. In this study, we demonstrated the usefulness of RMut through three case studies. First, we compared 10 different mutations predefined in RMut over real biological networks and found that the networks were most sensitive to overexpression/state-flip and edge-addition/-reverse mutations among node-based and edgetic mutations, respectively. In the second case study, we observed that edgetic mutations can predict drug-targets better than node-based mutations. Interestingly, edge-attenuation, which has not been considered in previous tools, showed high performance in drug-target prediction. Finally, we compared double and single edge-removal mutations based on network sensitivity values, and found an interesting synergy effect even for a pair of susceptible edges. Taken together, these findings indicate that RMut can be a useful tool to efficiently analyze network sensitivity against various types of mutations. In future, RMut could be extended to employ arbitrary update-rules or asynchronous update-scheme in the Boolean network model, and provide more visualization features for the analysis.

## Supporting information

S1 File Supporting figures and tables. The file includes the following: Figure A. Defining code of the state-flip mutation. Figure B. Defining code of the knockout mutation. Figure C. Defining code of the overexpression mutation. Figure D. Defining code of the outcome-shuffle mutation. Figure E. Defining code of the edge-removal mutation. Figure F. Defining code of the edge-addition mutation. Figure G. Defining code of the edge-attenuation mutation. Figure H. Defining code of the edge-sign-switch mutation. Figure I. Defining code of the edge-reverse mutation. Figure J. An example of network sensitivity analysis using user-defined NCF. Table A. List of the well-known mutations and their related studies. (PDF 134 KB)
(DOCX)

S2 File Detailed specification of all functions in RMut. (PDF 129 KB). (PDF)

S3 File User manual of RMut. (PDF 1,002 KB).
(PDF)
