# Supplementary material for: RMut: R package for a Boolean sensitivity analysis against various types of mutations
Source: PLoS One. 2019 Mar 19;14(3):e0213736. doi: 10.1371/journal.pone.0213736 (PMC6424452; doi:10.1371/journal.pone.0213736)
Supplement: S1 File — The file includes the following: Figure A. Defining code of the state-flip mutation. Figure B. Defining code of the knockout mutation. Figure C. Defining code of the overexpression mutation. Figure D. Defining code of the outcome-shuffle mutation. Figure E. Defining code of the edge-removal mutation. Figure F. Defining code of the edge-addition mutation. Figure G. Defining code of the edge-attenuation mutation. Figure H. Defining code of the edge-sign-switch mutation. Figure I. Defining code of the edge-reverse mutation. Figure J. An example of network sensitivity analysis using user-defined NCF. Table A. List of the well-known mutations and their related studies. (PDF 134 KB) (DOCX) [file pone.0213736.s001.docx]

**RMut: R package for a Boolean sensitivity analysis against various types of mutations**

Hung-Cuong Trinh ^1^ and Yung-Keun Kwon ^2,^*

^1^ Faculty of Information Technology, Ton Duc Thang University, Ho Chi Minh City, Vietnam

^2^ Department of Electrical/Electronic and Computer Engineering, University of Ulsan, 93 Daehak-ro, Nam-gu, Ulsan 680-749, Korea

^*^Corresponding author

E-mail: kwonyk@ulsan.ac.kr

Hung-Cuong Trinh

E-mail: trinhhungcuong@tdtu.edu.vn

Supporting Information

**Supplementary Figures**

Figure A. Defining code of the state-flip mutation.

package mod;

import java.util.ArrayList;

import java.util.concurrent.atomic.AtomicInteger;

public class **StateFlip** implements Mutation {

public void **nodeMutation**(ArrayList<Integer> input, ArrayList<Integer> I,

ArrayList<Integer> O, AtomicInteger O_default) {

int noInputs = input.size();

if(noInputs > 0) {

for (int k = 0; k < noInputs; k ++) {

O.set(k, 1 - O.get(k));

}

O_default.set(1 - O.get(noInputs - 1));

}

}

public void edgeMutation(int iSrc, ArrayList<Integer> tar_input, ArrayList<Integer> tar_I,

ArrayList<Integer> tar_O, AtomicInteger tar_O_default) {

}

}

Figure B. Defining code of the knockout mutation.

package mod;

import java.util.ArrayList;

import java.util.concurrent.atomic.AtomicInteger;

public class **Knockout** implements Mutation {

public void **nodeMutation**(ArrayList<Integer> input, ArrayList<Integer> I,

ArrayList<Integer> O, AtomicInteger O_default) {

int noInputs = input.size();

for (int k = 0; k < noInputs; k ++) {

O.set(k, 0);

}

O_default.set(0);

}

public void edgeMutation(int iSrc, ArrayList<Integer> tar_input, ArrayList<Integer> tar_I,

ArrayList<Integer> tar_O, AtomicInteger tar_O_default) {

}

}

Figure C. Defining code of the overexpression mutation.

package mod;

import java.util.ArrayList;

import java.util.concurrent.atomic.AtomicInteger;

public class **OverExpression** implements Mutation {

public void **nodeMutation**(ArrayList<Integer> input, ArrayList<Integer> I,

ArrayList<Integer> O, AtomicInteger O_default) {

int noInputs = input.size();

for (int k = 0; k < noInputs; k ++) {

O.set(k, 1);

}

O_default.set(1);

}

public void edgeMutation(int iSrc, ArrayList<Integer> tar_input, ArrayList<Integer> tar_I,

ArrayList<Integer> tar_O, AtomicInteger tar_O_default) {

}

}

Figure D. Defining code of the outcome-shuffle mutation.

package mod;

import java.util.ArrayList;

import java.util.concurrent.atomic.AtomicInteger;

public class **OutcomeShuffle** implements Mutation {

public void **nodeMutation**(ArrayList<Integer> input, ArrayList<Integer> I,

ArrayList<Integer> O, AtomicInteger O_default) {

int noInputs = input.size();

java.util.Collections.shuffle(O);

if(noInputs > 0) {

O_default.set(1 - O.get(noInputs - 1));

}

}

public void edgeMutation(int iSrc, ArrayList<Integer> tar_input, ArrayList<Integer> tar_I,

ArrayList<Integer> tar_O, AtomicInteger tar_O_default) {

}

}

Figure E. Defining code of the edge-removal mutation.

package mod;

import java.util.ArrayList;

import java.util.concurrent.atomic.AtomicInteger;

public class **EdgeRemoval** implements Mutation {

public void nodeMutation(ArrayList<Integer> input, ArrayList<Integer> I,

ArrayList<Integer> O, AtomicInteger O_default) {

}

public void **edgeMutation**(int iSrc, ArrayList<Integer> tar_input, ArrayList<Integer> tar_I,

ArrayList<Integer> tar_O, AtomicInteger tar_O_default) {

int k_source = tar_input.indexOf(iSrc);

if(k_source != -1) {

tar_input.remove(k_source);

tar_I.remove(k_source);

tar_O.remove(k_source);

}

int noInputs = tar_input.size();

if(noInputs > 0) {

tar_O_default.set(1 - tar_O.get(noInputs - 1));

} else {

tar_O_default.set(-1);

}

}

}

Figure F. Defining code of the edge-addition mutation.

package mod;

import java.util.ArrayList;

import java.util.concurrent.atomic.AtomicInteger;

import mod.jmut.core.comp.Node;

public class **EdgeAddition** implements Mutation {

public void nodeMutation(ArrayList<Integer> input, ArrayList<Integer> I,

ArrayList<Integer> O, AtomicInteger O_default) {

}

public void **edgeMutation**(int iSrc, ArrayList<Integer> tar_input, ArrayList<Integer> tar_I,

ArrayList<Integer> tar_O, AtomicInteger tar_O_default) {

int Ik, Ok;

int k = tar_input.size() + 1;

double temp = Math.exp(0 - Node.THETA * (Math.pow(2, -k)));

double probO = temp / (1 + temp);

Ik = (Math.random() < 0.5) ? 1 : 0;

Ok = (Math.random() < probO) ? 1 : 0;

//Insert the new edge into the first position in the input nodes

tar_input.add(0, iSrc);

tar_I.add(0, Ik);

tar_O.add(0, Ok);

if(k == 1) {

//Initially, the target node has no incoming links

tar_O_default.set(1 - Ok);

}

}

}

Figure G. Defining code of the edge-attenuation mutation.

package mod;

import java.util.ArrayList;

import java.util.concurrent.atomic.AtomicInteger;

public class **EdgeAttenuation** implements Mutation {

public void nodeMutation(ArrayList<Integer> input, ArrayList<Integer> I,

ArrayList<Integer> O, AtomicInteger O_default) {

}

public void **edgeMutation**(int iSrc, ArrayList<Integer> tar_input, ArrayList<Integer> tar_I,

ArrayList<Integer> tar_O, AtomicInteger tar_O_default) {

int k_source = tar_input.indexOf(iSrc);

int k_last = tar_input.size() - 1;

if(k_source != -1 && k_source != k_last) {

java.util.Collections.swap(tar_input, k_source, k_last);

java.util.Collections.swap(tar_I, k_source, k_last);

java.util.Collections.swap(tar_O, k_source, k_last);

tar_O_default.set(1 - tar_O.get(k_last));

}

}

}

Figure H. Defining code of the edge-sign-switch mutation.

package mod;

import java.util.ArrayList;

import java.util.concurrent.atomic.AtomicInteger;

public class **EdgeSignSwitch** implements Mutation {

public void nodeMutation(ArrayList<Integer> input, ArrayList<Integer> I,

ArrayList<Integer> O, AtomicInteger O_default) {

}

public void **edgeMutation**(int iSrc, ArrayList<Integer> tar_input, ArrayList<Integer> tar_I,

ArrayList<Integer> tar_O, AtomicInteger tar_O_default) {

int k_source = tar_input.indexOf(iSrc);

if(k_source != -1) {

tar_I.set(k_source, 1 - tar_I.get(k_source));

}

}

}

Figure I. Defining code of the edge-reverse mutation.

package mod;

import java.util.ArrayList;

import java.util.concurrent.atomic.AtomicInteger;

public class **EdgeReverse** implements Mutation {

public void nodeMutation(ArrayList<Integer> input, ArrayList<Integer> I,

ArrayList<Integer> O, AtomicInteger O_default) {

}

public void **edgeMutation**(int iSrc, ArrayList<Integer> tar_input, ArrayList<Integer> tar_I,

ArrayList<Integer> tar_O, AtomicInteger tar_O_default) {

int k_source = tar_input.indexOf(iSrc);

if(k_source != -1) {

tar_O.set(k_source, 1 - tar_O.get(k_source));

if(k_source == tar_input.size() - 1) {

tar_O_default.set(1 - tar_O.get(k_source));

}

}

}

}

Figure J. An example of network sensitivity analysis using user-defined NCF.

# load an example network, the large-scale human signaling network

data(hsn)

# setup OpenCL for parallel computation

setOpencl("gpu")

# generate 1000 random initial-states

states <- generateStates(hsn, 1000)

# generate all possible groups each containing a single node in the HSN network

hsn <- generateGroups(hsn, "all", 1, 0)

# calculate sensitivity values against a knockout mutation over user-defined NCF rules

hsn <- **calSensitivity**(hsn, states, "knockout", ruleFile = "D:\\mod\\**UserNCF.txt**")

# view the calculated sensitivity values and export all results to files

printSensitivity(hsn)

output(hsn)

**Supplementary Tables**

**Table A. List of the well-known mutations and their related studies.**

| **Category** | **Mutation name** | **References** |
| --- | --- | --- |
| Node-based mutations | State-flip | ([Elmeligy Abdelhamid *et al.*, 2015](#_ENREF_10); [Kwon *et al.*, 2007](#_ENREF_16); [Müssel *et al.*, 2010](#_ENREF_22); [Paroni *et al.*, 2016](#_ENREF_26); [Shmulevich *et al.*, 2002](#_ENREF_28); [Trinh *et al.*, 2014](#_ENREF_30)) |
|  | Rule-flip | ([Kwon *et al.*, 2007](#_ENREF_16); [Kwon *et al.*, 2015](#_ENREF_17); [Le *et al.*, 2013](#_ENREF_18); [Trinh *et al.*, 2015](#_ENREF_29); [Trinh *et al.*, 2014](#_ENREF_30)) |
|  | Knockout | ([Azuaje *et al.*, 2010](#_ENREF_2); [Calzone *et al.*, 2015](#_ENREF_4); [Davidich *et al.*, 2013](#_ENREF_8); [Hetmanski *et al.*, 2016](#_ENREF_12); [Kwon *et al.*, 2015](#_ENREF_17); [Li *et al.*, 2006](#_ENREF_20); [Ortiz-Gutiérrez *et al.*, 2015](#_ENREF_24)) |
|  | Overexpression | ([Azuaje *et al.*, 2010](#_ENREF_2); [Calzone *et al.*, 2015](#_ENREF_4); [Li *et al.*, 2006](#_ENREF_20)) |
|  | Outcome-shuffle | ([Müssel *et al.*, 2010](#_ENREF_22); [Xiaoning *et al.*, 2008](#_ENREF_34)) |
| Edgetic mutations | Edge-attenuation | ([Ágoston *et al.*, 2005](#_ENREF_1); [Csermely *et al.*, 2005](#_ENREF_7); [Grindrod *et al.*, 2014](#_ENREF_11)) |
|  | Edge-removal | ([Li *et al.*, 2004](#_ENREF_19); [Murrugarra *et al.*, 2015](#_ENREF_21); [Nepusz *et al.*, 2012](#_ENREF_23); [Pardo *et al.*, 2015](#_ENREF_25); [Sahni *et al.*, 2015](#_ENREF_27); [Wang *et al.*, 2014](#_ENREF_31); [Wang *et al.*, 2012](#_ENREF_32)) |
|  | Edge-addition | ([Beygelzimer *et al.*, 2005](#_ENREF_3); [Campbell *et al.*, 2014](#_ENREF_5); [Chan *et al.*, 2016](#_ENREF_6); [Li *et al.*, 2004](#_ENREF_19); [Yang *et al.*, 2015](#_ENREF_35)) |
|  | Edge-sign-switch | ([Li *et al.*, 2004](#_ENREF_19)) |
|  | Edge-reverse | ([Dehghannasiri *et al.*, 2015](#_ENREF_9); [Hu *et al.*, 2016](#_ENREF_13); [Müssel *et al.*, 2010](#_ENREF_22); [Xiao *et al.*, 2007](#_ENREF_33); [Xiaoning *et al.*, 2008](#_ENREF_34)) |
|  |  |  |

**References**

Ágoston, V., Csermely, P. and Pongor, S. (2005) Multiple, weak hits confuse complex systems: A transcriptional regulatory network as an example, *Physical Review E*, **71**, 051909.

Azuaje, F., Devaux, Y. and Wagner, D.R. (2010) Identification of potential targets in biological signalling systems through network perturbation analysis, *Biosystems*, **100**, 55-64.

Beygelzimer, A.*, et al.* (2005) Improving network robustness by edge modification, *Physica A: Statistical Mechanics and its Applications*, **357**, 593-612.

Calzone, L., Barillot, E. and Zinovyev, A. (2015) Predicting genetic interactions from Boolean models of biological networks, *Integrative Biology*, **7**, 921-929.

Campbell, C. and Albert, R. (2014) Stabilization of perturbed Boolean network attractors through compensatory interactions, *BMC Systems Biology*, **8**, 1-16.

Chan, H. and Akoglu, L. (2016) Optimizing network robustness by edge rewiring: a general framework, *Data Mining and Knowledge Discovery*, 1-31.

Csermely, P., Ágoston, V. and Pongor, S. (2005) The efficiency of multi-target drugs: the network approach might help drug design, *Trends in Pharmacological Sciences*, **26**, 178-182.

Davidich, M.I. and Bornholdt, S. (2013) Boolean Network Model Predicts Knockout Mutant Phenotypes of Fission Yeast, *PLoS ONE*, **8**, e71786.

Dehghannasiri, R., Yoon, B.J. and Dougherty, E.R. (2015) Optimal Experimental Design for Gene Regulatory Networks in the Presence of Uncertainty, *IEEE/ACM Transactions on Computational Biology and Bioinformatics*, **12**, 938-950.

Elmeligy Abdelhamid, S.H.*, et al.* (2015) GDSCalc: A Web-Based Application for Evaluating Discrete Graph Dynamical Systems, *PLoS ONE*, **10**, e0133660.

Grindrod, P. and Higham, D.J. (2014) A dynamical systems view of network centrality, *Proceedings of the Royal Society of London A: Mathematical, Physical and Engineering Sciences*, **470**.

Hetmanski, J.H.R.*, et al.* (2016) A MAPK-Driven Feedback Loop Suppresses Rac Activity to Promote RhoA-Driven Cancer Cell Invasion, *PLoS Comput Biol*, **12**, e1004909.

Hu, M.*, et al.* (2016) An efficient algorithm to identify the optimal one-bit perturbation based on the basin-of-state size of Boolean networks, *Scientific Reports*, **6**, 26247.

Kauffman, S.*, et al.* (2003) Random Boolean network models and the yeast transcriptional network, *Proceedings of the National Academy of Sciences*, **100**, 14796-14799.

Kauffman, S.*, et al.* (2004) Genetic networks with canalyzing Boolean rules are always stable, *Proceedings of the National Academy of Sciences of the United States of America*, **101**, 17102-17107.

Kwon, Y.-K., Choi, S. and Cho, K.-H. (2007) Investigations into the relationship between feedback loops and functional importance of a signal transduction network based on Boolean network modeling, *BMC Bioinformatics*, **8**, 384.

Kwon, Y.-K., Kim, J. and Cho, K.-H. (2015) Dynamical Robustness Against Multiple Mutations in Signaling Networks, *Computational Biology and Bioinformatics, IEEE/ACM Transactions on*, **PP**.

Le, D.-H. and Kwon, Y.-K. (2013) A coherent feedforward loop design principle to sustain robustness of biological networks, *Bioinformatics*, **29**, 630-637.

Li, F.*, et al.* (2004) The yeast cell-cycle network is robustly designed, *Proceedings of the National Academy of Sciences of the United States of America*, **101**, 4781-4786.

Li, S., Assmann, S.M. and Albert, R. (2006) Predicting Essential Components of Signal Transduction Networks: A Dynamic Model of Guard Cell Abscisic Acid Signaling, *PLoS Biol*, **4**, e312.

Murrugarra, D. and Dimitrova, E.S. (2015) Molecular network control through boolean canalization, *EURASIP Journal on Bioinformatics and Systems Biology*, **2015**, 9.

Müssel, C., Hopfensitz, M. and Kestler, H.A. (2010) BoolNet—an R package for generation, reconstruction and analysis of Boolean networks, *Bioinformatics*, **26**, 1378-1380.

Nepusz, T. and Vicsek, T. (2012) Controlling edge dynamics in complex networks, *Nat Phys*, **8**, 568-573.

Ortiz-Gutiérrez, E.*, et al.* (2015) A Dynamic Gene Regulatory Network Model That Recovers the Cyclic Behavior of Arabidopsis thaliana Cell Cycle, *PLoS Comput Biol*, **11**, e1004486.

Pardo, E.P. and Godzik, A. (2015) Analysis of Individual Protein Regions Provides Novel Insights on Cancer Pharmacogenomics, *PLoS Comput Biol*, **11**, e1004024.

Paroni, A.*, et al.* (2016) CABeRNET: a Cytoscape app for augmented Boolean models of gene regulatory NETworks, *BMC Bioinformatics*, **17**, 1-12.

Sahni, N.*, et al.* (2015) Widespread Macromolecular Interaction Perturbations in Human Genetic Disorders, *Cell*, **161**, 647-660.

Shmulevich, I., Dougherty, E.R. and Zhang, W. (2002) Gene perturbation and intervention in probabilistic Boolean networks, *Bioinformatics*, **18**, 1319-1331.

Trinh, H.-C. and Kwon, Y.-K. (2015) Effective Boolean dynamics analysis to identify functionally important genes in large-scale signaling networks, *Biosystems*, **137**, 64-72.

Trinh, H.-C., Le, D.-H. and Kwon, Y.-K. (2014) PANET: A GPU-Based Tool for Fast Parallel Analysis of Robustness Dynamics and Feed-Forward/Feedback Loop Structures in Large-Scale Biological Networks, *PLoS ONE*, **9**, e103010.

Wang, W.*, et al.* (2014) Mutations that disrupt PHOXB interaction with the neuronal calcium sensor HPCAL1 impede cellular differentiation in neuroblastoma, *Oncogene*, **33**, 3316-3324.

Wang, X.*, et al.* (2012) Three-dimensional reconstruction of protein networks provides insight into human genetic disease, *Nat Biotech*, **30**, 159-164.

Xiao, Y. and Dougherty, E.R. (2007) The impact of function perturbations in Boolean networks, *Bioinformatics*, **23**, 1265-1273.

Xiaoning, Q. and Dougherty, E.R. (2008) Effect of Function Perturbation on the Steady-State Distribution of Genetic Regulatory Networks: Optimal Structural Intervention, *Signal Processing, IEEE Transactions on*, **56**, 4966-4976.

Yang, Y.*, et al.* (2015) Improving the Robustness of Complex Networks with Preserving Community Structure, *PLoS ONE*, **10**, e0116551.
