# Supplementary material for: RMut: R package for a Boolean sensitivity analysis against various types of mutations
Source: PLoS One. 2019 Mar 19;14(3):e0213736. doi: 10.1371/journal.pone.0213736 (PMC6424452; doi:10.1371/journal.pone.0213736)
Supplement: S2 File — (PDF) [file pone.0213736.s002.pdf]

Package ‘RMut’

November 25, 2018

Type Package

Title RMut: a collective framework for mutations analysis in network dynamics

Version 0.1.0

Author Hung-Cuong Trinh, Yung-Keun Kwon

Maintainer Hung-Cuong Trinh <hungcuong1986@gmail.com>

Description Provides functions for intensely analyzing dynamics of biological networks and also random networks based on different kinds of mutations.

License Apache License 2.0

LazyData TRUE

Depends rJava (>= 0.9-8)

RoxygenNote 6.1.1

Suggests knitr,
rmarkdown

VignetteBuilder knitr

R topics documented:

RMut-package 2
amrn 5
calCentrality 6
calSensitivity 7
cchs 8
ccsn 9
cdrn 9
createRBNs 10
findAttractors 11
findFBLs 12
findFFLs 13
generateGroup 14
generateGroups 14
generateRule 15
generateState 16
generateStates 17
hsn 18
initJVM 18
loadNetwork 19

output 20
perturb 20
printSensitivity 22
restore 23
setOpencl 24
showOpencl 25

Index **26**

`RMut-package` *An R package for investigating different types of mutations in network dynamics analyses.*

Description

The RMut package provides some categories of useful functions for examining dynamics of biological networks based on many kinds of mutations. The package also computes some node/edge-based structural characteristics of the networks.

Details

This package provides useful functions for intensely analyzing dynamics of biological networks and also random networks based on different kinds of mutations. For those analyses, the package utilizes a Boolean network model with synchronous updating scheme. The package also examines some node/edge-based structural characteristics of the networks. In summary, there are four main types of functions in the package, Setup functions, Data handling functions, Dynamics-related functions and Structure-related functions, as follows:

Setup functions

Core algorithms of dynamics analyses and feedback/feed-forward loops search were processed in parallel using an OpenCL parallel computing platform, which is an open-source library designed to run on any modern central processing units (CPUs) or graphics processing units (GPUs). Thus, the package can be used on any computer equipped with multi-core CPUs and/or GPUs that can support the OpenCL library. The `showOpencl` function shows installed OpenCL platforms and its corresponding CPU/GPU devices. `setOpencl` enables OpenCL computation by selecting a CPU/GPU device and utilizing all of its cores for further computation.

Data handling functions

Networks can be loaded in two ways using RMut: the `loadNetwork` function creates a network from a Tab-separated values text file. Otherwise, the package provides some example networks that could be simply loaded by `data` command. Furthermore, random networks of four generation models could be generated by using the `createRBNs` function.

Via `output`, all examined attributes of the networks will be exported to CSV files.

Dynamics-related functions

The `findAttractors` function identifies attractors in a network, and the resulted transition network could be exported by the function `output`. Then, those resulted files could be further loaded and analyzed by other softwares with powerful visualization functions like Cytoscape. In addition, the package also provides two other functions to manually investigate the network dynamics: `perturb` and `restore`. The `perturb` function makes perturbations on a set of node/edge groups in the examined network. `restore` puts a set of node/edge groups in the examined network back to its normal condition. Hence, we can manually compare the converged attractors before and after perturbations by utilizing those three functions.

The calSensitivity function computes sensitivity values of node/edge groups in the examined networks. Two kinds of sensitivity measures are computed: macro-distance and bitwise-distance sensitivity measures. We can use embedded mutations in the package or define our own mutations. Multiple sets of random Nested Canalyzing rules could be specified, and thus resulted in multiple sensitivity values for each group.

Structure-related functions

Via findFBLs and findFFLs, the package supports methods of searching feedback/feed-forward loops, respectively, for all nodes/edges in the examined networks.

The calCentrality function calculates node-/edge-based centralities of the examined networks such as Degree, In-/Out-Degree, Closeness, Betweenness, Stress, Eigenvector, Edge Degree and Edge Betweenness.

Author(s)

Hung-Cuong Trinh, Yung-Keun Kwon

Examples

```
#######################################
# Example 1: single network analyses #
#######################################

# setup OpenCL
showOpencl()
setOpencl("cpu")

# load example network
data(amrn)

### Calculate sensitivity values based on various types of mutations, and structural measures ###
# generate all possible initial-states each containing 10 Boolean nodes
set1 <- generateStates(10, "all")
print(set1)

# generate all possible groups each containing a single node in the AMRN network
amrn <- generateGroups(amrn, "all", 1, 0)
amrn <- calSensitivity(amrn, set1, "knockout")
print(amrn$Group_1)

# generate all possible groups each containing a single edge in the AMRN network
amrn <- generateGroups(amrn, "all", 0, 1)
amrn <- calSensitivity(amrn, set1, "edge removal")
print(amrn$Group_2)

# generate all possible groups each containing a new edge (the edge did not exist in the AMRN network)
amrn <- generateGroups(amrn, "all", 0, 1, TRUE)
amrn <- calSensitivity(amrn, set1, "edge addition")
print(amrn$Group_3)

# employ a user-defined mutation
amrn <- calSensitivity(amrn, set1, "D:\\dyna\\mod\\MyMutation.java", 1)
print(amrn$Group_1)

# search feedback/feed-forward loops
amrn <- findFBLs(amrn, maxLength = 10)
```

```
amrn <- findFFLs(amrn)

# calculate node-/edge-based centralities
amrn <- calCentrality(amrn)

# export all results to CSV files
output(amrn)


### Identify attractor cycles ###
# generate a set of random Nested Canalyzing rules
generateRule(amrn, 2)

# generate a specific initial-state for the AMRN network
state1 <- generateState(amrn, "1110011011")

# find the original transition network (before making perturbations)
transNet <- findAttractors(amrn, state1)
print(transNet)
output(transNet)


### Perturb and restore a node/edge group
# generate a group of two nodes and two edges in the AMRN network
amrn <- generateGroup(amrn, nodes = "AG, SUP", edges = "UFO (1) PI, LUG (-1) PI")
print(amrn$Group_4)

# perturb the group with overexpression mutation,
#  in this case only two nodes (AG, SUP) of the group are affected by the mutation.
perturb(amrn, 4, "overexpression")

# continuously perturb the group with edge-removal mutation,
#  in this case only two edges of the group are removed by the mutation.
perturb(amrn, 4, "edge removal")

# continuously perturb the group with "state flip" mutation,
#  thus only two nodes (AG, SUP) of the group are affected by the mutation.
perturb(amrn, 4, "state flip")

# find the perturbed transition network
perturbed_transNet <- findAttractors(amrn, state1)
print(perturbed_transNet)

# restore the group and initial-state from previous mutations
restore(amrn, 4)

# find again the original transition network, it should be same with the "transNet" network
origin_transNet <- findAttractors(amrn, state1)
print(origin_transNet)



##################################
# Example 2: Batch-mode analyses #
##################################

# generate all possible initial-states each containing 10 Boolean nodes
```

```
set1 <- generateStates(10, "all")

### generate random networks based on BA model ###
ba_rbns <- createRBNs("BA_RBN_", 2, "BA", 10, 17)

# for each random network, generate all possible groups each containing a single node
ba_rbns <- generateGroups(ba_rbns, "all", 1, 0)

# for each random network, calculate the sensitivity values of all nodes against "knockout" mutation
ba_rbns <- calSensitivity(ba_rbns, set1, "knockout")

# for each random network, calculate structural measures of all nodes/edges
ba_rbns <- findFBLs(ba_rbns, maxLength = 10)
ba_rbns <- findFFLs(ba_rbns)
ba_rbns <- calCentrality(ba_rbns)

print(ba_rbns)
output(ba_rbns)

### generate random networks based on "Shuffle 2" model ###
amrn_rbns <- createRBNs("AMRN_RBN_", 2, "shuffle 2", referedNetwork = amrn)

# for each random network, generate all possible groups each containing a single edge
amrn_rbns <- generateGroups(amrn_rbns, "all", 0, 1)

# for each random network, calculate the sensitivity values of all edges against "remove" mutation
amrn_rbns <- calSensitivity(amrn_rbns, set1, "edge removal")

# for each random network, calculate structural measures of all nodes/edges
amrn_rbns <- findFBLs(amrn_rbns, maxLength = 10)
amrn_rbns <- findFFLs(amrn_rbns)
amrn_rbns <- calCentrality(amrn_rbns)

print(amrn_rbns)
output(amrn_rbns)
```

`amrn` *Arabidopsis morphogenesis regulatory network.*

Description

The Arabidopsis morphogenesis regulatory network (AMRN) with 10 nodes and 22 links. This regulatory network is known to robustly control the process of flower development.

Usage

```
data(amrn)
```

Format

A data frame with 22 rows and 3 variables:

`Source` the identifier of the source node

`Interaction` interaction type of the edge

`Target` the identifier of the target node ...

Source

http://www.ncbi.nlm.nih.gov/pubmed/10487867

`calCentrality` *Calculate node-/edge-based centralities of a network or a set of networks.*

Description

Calculate centrality measures for all nodes/edges in a network or in a set of networks.

Usage

```
calCentrality(networks)
```

Arguments

`networks`	A network or a set of networks used for the calculation

Details

This function calculates node-/edge-based centralities of a specific network or a set of networks. For each network, the returned results are stored in a data frame of the network object. The data frame has one column for nodes/edges identifiers, and nine columns contain corresponding values of some node-based centrality measures such as Degree, In-/Out-Degree, Closeness, Betweenness, Stress and Eigenvector, and some edge-based measures like Edge Degree and Edge Betweenness.

Value

The updated network objects including values of centrality measures for each node/edge.

See Also

`findFBLs`, `findFFLs`, `calSensitivity`

Examples

```
data(amrn)
amrn <- calCentrality(amrn)
print(amrn$nodes)
print(amrn$edges)
```

calSensitivity *Compute sensitivity values of node/edge groups*

Description

Computes sensitivity values of node/edge groups in a network or in a set of networks, and returns the network objects with newly calculated results.

Usage

```
calSensitivity(networks, stateSet, mutateMethod = "rule flip",
            groupSet = 0, mutationTime = 1000L, numRuleSets = 1, ruleFile = NULL)
```

Arguments

`networks`	A network or a set of networks used for the calculation
`stateSet`	The identifier for accessing a set of initial-states
`mutateMethod`	The method of mutation to be performed, default is "rule flip"
`groupSet`	The indexing number of node/edge groups for whose sensitivity values are calculated. Default is 0 which specify the latest generated groups.
`mutationTime`	The period of time in which the mutation occurs, default is 1000
`numRuleSets`	Number of random Nested Canalyzing Function sets, default is 1
`ruleFile`	The path points to a file containing user-defined Nested Canalyzing Function rules, default is NULL. In case the path is specified, the parameter `numRuleSets` is forced to 1.

Details

This function computes sensitivity values of node/edge groups in a specific network or in a set of networks. Two kinds of sensitivity measures are computed: macro-distance and bitwise-distance sensitivity measures.

The calculation is based on a set of initial-states specified by an identifier `stateSet`. The node/edge groups in each network are determined by an indexing number `groupSet`. For example, the number `1` would point to the data frame of node/edge groups named `Group_1`. For mutation settings, there exist some embedded mutations: "state flip", "rule flip", "outcome shuffle", "knockout", "overexpression", "edge removal", "edge attenuation", "edge addition", "edge sign switch", and "edge reverse". Besides, users can define their own mutation and apply here as shown in the below example. Users can also set the operational time of the mutation as determined by the parameter `mutationTime`. Finally, synchronous updating scheme is used for calculating state transitions. Single or multiple sets of random update-rules are generated based on the parameter `numRuleSets`. A file containing user-defined rules could be specified by the parameter `ruleFile`.

For each network, the sensitivity values are stored in the same data frame of node/edge groups. The data frame has one column for group identifiers (lists of nodes/edges), and some next columns containing their sensitivity values according to each set of random update-rules.

Value

The updated network objects including sensitivity values of the examined node/edge groups.

See Also

generateStates, generateState, generateGroups, generateGroup, findFBLs, findFFLs, calCentrality, findAttractors

Examples

```
# load an example network, the large-scale human signaling network
data(hsn)

# setup OpenCL for parallel computation
setOpencl("gpu")

# generate 1000 random initial-states
states <- generateStates(hsn, 1000)
print(states)

# generate all possible groups each containing a single node in the HSN network
hsn <- generateGroups(hsn, "all", 1, 0)

# calculate sensitivity values of all nodes against the knockout mutation
hsn <- calSensitivity(hsn, states, "knockout")

# calculate sensitivity values against a user-defined mutation, also use user-defined rules
hsn <- calSensitivity(hsn, states, "D:\\mod\\UserMutation.java", ruleFile="D:\\mod\\UserNCF.txt")

# view the calculated sensitivity values and export all results to files
printSensitivity(hsn)
output(hsn)
```

---

cchs *Cell cycle pathway of the species Homo sapiens.*

Description

The cell cycle pathway of the species Homo sapiens (CCHS) with 161 nodes and 223 links. The cell cycle is the series of events that takes place in a cell leading to its division and duplication (replication).

Usage

```
data(cchs)
```

Format

A data frame with 223 rows and 3 variables:

- `Source` the identifier of the source node
- `Interaction` interaction type of the edge
- `Target` the identifier of the target node ...

Source

https://www.ncbi.nlm.nih.gov/pubmed/9733515

`ccsn` *Canonical cell signaling network.*

Description

The canonical cell signaling network (CCSN) with 771 nodes and 1633 links. The network was obtained from http://stke.sciencemag.org/, and all the neutral interactions were excluded.

Usage

```
data(ccsn)
```

Format

A data frame with 1633 rows and 3 variables:

`Source` the identifier of the source node

`Interaction` interaction type of the edge

`Target` the identifier of the target node ...

Source

http://bioinformatics.oxfordjournals.org/content/24/17/1926.long

`cdrn` *Cell differentiation regulatory network.*

Description

The cell differentiation regulatory network (CDRN) with 9 nodes and 15 links. CDRN has seven positive and two negative FBLs is found to robustly induce quiescence, terminal differentiation, and apoptosis.

Usage

```
data(cdrn)
```

Format

A data frame with 15 rows and 3 variables:

`Source` the identifier of the source node

`Interaction` interaction type of the edge

`Target` the identifier of the target node ...

Source

http://www.ncbi.nlm.nih.gov/pubmed/11082279

createRBNs *Create a set of random networks.*

Description

Create a set of random networks using different generation models.

Usage

```
createRBNs(prexName = "RBN_", numNetworks = 10, model = "BA",
           numNodes = 10, numEdges = 20, probOfNegative = 0.5,
           referedNetwork = NULL, shuffleRate = 4.0)
```

Arguments

prexName	The common prefix of generated random network names, default is "RBN_"
numNetworks	The number of generated random networks, default is 10
model	The specified generation model from among four models: BA, ER, Shuffle 1 and Shuffle 2. Default is "BA".
numNodes	The number of nodes in a random network, default is 10
numEdges	The number of edges in a random network, default is 20
probOfNegative	The probability of negative links's assignment in a random network, default is 0.5
referedNetwork	The specific reference network used for two shuffling models
shuffleRate	The shuffling intensity of "Shuffle 2" model. The number of rewiring steps = (Shuffling intensity) x (number of edges). Default is 4.

Details

This function generates a set of random networks using a generation model from among four models: Barabasi-Albert (BA) model [1], Erdos-Renyi (ER) variant model [2] and two shuffling models (Shuffle 1 and Shuffle 2) [3]. Refer to the literature in the References section for more details.

Value

The generated random network objects.

References

1. Barabasi A-L, Albert R (1999) Emergence of Scaling in Random Networks. Science 286: 509-512. doi: 10.1126/science.286.5439.509

2. Le D-H, Kwon Y-K (2011) NetDS: A Cytoscape plugin to analyze the robustness of dynamics and feedforward/feedback loop structures of biological networks. Bioinformatics.

3. Trinh H-C, Le D-H, Kwon Y-K (2014) PANET: A GPU-Based Tool for Fast Parallel Analysis of Robustness Dynamics and Feed-Forward/Feedback Loop Structures in Large-Scale Biological Networks. PLoS ONE 9: e103010.

See Also

loadNetwork, calSensitivity, generateStates, generateState, generateGroups, generateGroup, findFBLs, findFFLs, calCentrality

Examples

```
# Generate all possible initial-states each containing 10 Boolean nodes
set1 <- generateStates(10, "all")

# Generate random networks based on BA model
ba_rbns <- createRBNs("BA_RBN_", 2, "BA", 10, 17)

# For each random network, generate all possible groups each containing a single node
ba_rbns <- generateGroups(ba_rbns, "all", 1, 0)

# For each random network, calculate sensitivity values of all nodes against "knockout" mutation
ba_rbns <- calSensitivity(ba_rbns, set1, "knockout")

# For each random network, calculate structural measures of all nodes/edges
ba_rbns <- findFBLs(ba_rbns, maxLength = 10)
ba_rbns <- findFFLs(ba_rbns)
ba_rbns <- calCentrality(ba_rbns)

print(ba_rbns)
output(ba_rbns)
```

---

findAttractors — *Identifies attractors of a network.*

Description

Identifies attractors of a network and returns the resulted transition network.

Usage

```
findAttractors(network, stateSet)
```

Arguments

network	A network used for the attractors search
stateSet	The identifier for accessing a set of initial-states

Details

This function searches attractors of a specific network, and the returned results are stored in a Transition network object. The calculation is based on a set of initial-states specified by an identifier `stateSet`. The current set of update-rules of the network is used with a synchronous updating scheme.

Value

The resulted transition network.

See Also

generateStates, generateState, generateRule, calSensitivity

Examples

```
data(amrn)
# Generate a set of random Nested Canalyzing rules
generateRule(amrn, 2)

# Generate a specific initial-state for the AMRN network
state1 <- generateState(amrn, "1110011011")

transNet <- findAttractors(amrn, state1)
print(transNet)
output(transNet)
```

---

findFBLs *Identifies feedback loops in a network or in a set of networks.*

Description

Searches and counts feedback loops for all nodes/edges in a network or in a set of networks.

Usage

```
findFBLs(networks, maxLength = 2L)
```

Arguments

networks	A network or a set of networks used for FBLs search
maxLength	The maximal length of FBLs, default is 2

Details

This function searches feedback loops (FBLs) in a specific network or in a set of networks. For each network, the returned results are stored in two corresponding data frames of node/edge attributes. Each data frame has one column for node/edge identifiers, and three columns contain corresponding number of involved FBLs and number of involved positive/negative FBLs. Another data frame of the network object, "network" data frame, contains total number of FBLs and total number of positive/negative FBLs in the network.

Value

The updated network objects including number of FBLs for each node/edge.

See Also

findFFLs, calCentrality, calSensitivity

Examples

```
data(amrn)
amrn <- findFBLs(amrn, maxLength = 10)
print(amrn$nodes)
print(amrn$edges)
print(amrn$network)
```

`findFFLs` *Identifies feedforward loops in a network or in a set of networks.*

Description

Searches and counts feedforward loops for all nodes/edges in a network or in a set of networks.

Usage

```
findFFLs(networks)
```

Arguments

`networks`	A network or a set of networks used for FFLs search

Details

This function searches feedforward loops (FFLs) in a specific network or in a set of networks. For each network, the returned results are stored in two corresponding data frames of node/edge attributes. Each data frame has one column for node/edge identifiers, and four columns contain corresponding number of all FFL motifs and number of FFL motifs with three different roles A, B and C. Another data frame of the network object, "network" data frame, contains total numbers of FFLs and coherent/incoherent FFLs in the network.

Value

The updated network objects including number of FFL motifs for each node/edge.

See Also

`findFBLs`, `calCentrality`, `calSensitivity`

Examples

```
data(amrn)
amrn <- findFFLs(amrn)
print(amrn$nodes)
print(amrn$edges)
print(amrn$network)
```

generateGroup *Generate a specific group of nodes/edges.*

Description

Generate a specific group of nodes/edges in a network.

Usage

```
generateGroup(network, nodes, edges = "")
```

Arguments

network	The network contains the generated group
nodes	A list of nodes in the generated group
edges	A list of edges in the generated group

Details

This function generates a specific group of elements in a network. The group would be used to analyze the dynamics of the examined network, for ex., calculating sensitivity, perturbing the network, or restoring the network to the origin. The element group contains only nodes, only edges, or a combination of nodes/edges.

Value

The updated network object including the generated group

See Also

calSensitivity, perturb, restore

Examples

```
data(amrn)
# Generate a group of two nodes and two edges in the AMRN network
amrn <- generateGroup(amrn, nodes = "AG, SUP", edges = "UFO (1) PI, LUG (-1) PI")
print(amrn$Group_1)
```

generateGroups *Generate random node/edge groups.*

Description

Generate random groups of node/edge in a network or in a set of networks.

Usage

```
generateGroups(networks, numGroups, nodeSize = 1, edgeSize = 0, newEdges = FALSE)
```

Arguments

networks	A network or a set of networks contain the generated groups
numGroups	Number of random groups to be generated for each network. If set to "all", all possible groups would be generated.
nodeSize	Number of nodes in each group, default is 1
edgeSize	Number of edges in each group, default is 0
newEdges	If TRUE, new edges would be created for each group. Otherwise, existing edges of the networks are selected for each group.

Details

This function generates random groups of elements in a network or in a set of networks. The groups would be used to analyze the dynamics of the examined networks, for ex., calculating sensitivity, perturbing a network, or restoring a network to the origin. Each element group contains only nodes, only edges, or a combination of nodes/edges.

Value

The updated network objects including generated groups

See Also

calSensitivity, perturb, restore

Examples

```
data(amrn)
# Generate all possible groups each containing a single node in the AMRN network
amrn <- generateGroups(amrn, "all", 1, 0)
print(amrn$Group_1)

# Generate all possible groups each containing a single edge in the AMRN network
amrn <- generateGroups(amrn, "all", 0, 1)
print(amrn$Group_2)

# Generate all possible groups each containing a new edge (the edge did not exist in the AMRN network)
amrn <- generateGroups(amrn, "all", 0, 1, TRUE)
print(amrn$Group_3)
```

---

generateRule *Generate a default set of update-rules.*

Description

Generate a default set of update-rules for a network.

Usage

```
generateRule(network, ruleType = 0L)
```

Arguments

`network`	The network used for the generation
`ruleType`	Type of random update-rules, default is 0

Details

This function generates a default set of update-rules for a network. The rules would be used to analyze the dynamics of the examined network, for ex., calculating sensitivity, searching attractors. The type of random update-rules can be specified by the parameter `ruleType`: 0 means only Conjunction rules, 1 means only Disjunction rules and 2 means random Nested Canalyzing rules.

Value

The string "ok" if success, otherwise NULL object

See Also

`findAttractors`, `perturb`, `restore`

Examples

```
data(amrn)
# Generate a set of random Nested Canalyzing rules
generateRule(amrn, 2)

# Generate a specific initial-state for the AMRN network
state1 <- generateState(amrn, "1110011011")

att <- findAttractors(amrn, state1)
print(att)
```

`generateState` *Generate a specific initial-state.*

Description

Generate a specific initial-state for a network.

Usage

```
generateState(network, state)
```

Arguments

`network`	The network used for the generation
`state`	A binary string with one entry for each node of the network in alphabetical order

Details

This function generates a specific initial-state for a network. The initial-state would be used to analyze the dynamics of the examined network, for ex., calculating sensitivity or searching attractors.

Value

An identifier for accessing the generated initial-state. The identifier would be used as a parameter of the functions of calculating sensitivity and finding attractors.

See Also

calSensitivity, findAttractors

Examples

```
data(amrn)
state1 <- generateState(amrn, "1010101111")
print(state1)
```

---

generateStates *Generate random initial-states.*

Description

Generate random initial-states for a network or a set of networks.

Usage

```
generateStates(numNodes, numStates)
```

Arguments

numNodes	Number of nodes in each initial-state or a network object
numStates	Number of random initial-states to be generated. If set to "all", all possible initial-states would be generated. For the large networks, we should use a specific value becaused of memory limitation.

Details

This function generates random initial-states for a network or a set of networks. The initial-states would be used to analyze the dynamics of the examined networks, for ex., calculating sensitivity or searching attractors.

Value

An identifier for accessing the generated initial-states. The identifier would be used as a parameter of the functions of calculating sensitivity and finding attractors.

See Also

calSensitivity, findAttractors

Examples

```
# Generate a set of 200 initial-states each containing 10 Boolean nodes
set1 <- generateStates(10, 200)
print(set1)

# Generate all possible initial-states each containing 10 Boolean nodes
set2 <- generateStates(10, "all")
print(set2)
```

`hsn` *Human signaling network.*

Description

The large-scale human signaling network (HSN) with 1192 nodes and 3102 links. Based on the network, some general principles were provided for understanding protein evolution in the context of signaling networks.

Usage

```
data(hsn)
```

Format

A data frame with 3102 rows and 3 variables:

`Source` the identifier of the source node

`Interaction` interaction type of the edge

`Target` the identifier of the target node ...

Source

http://www.ncbi.nlm.nih.gov/pubmed/19226461

`initJVM` *Initialize the Java Virtual Machine.*

Description

`initJVM` initializes the Java Virtual Machine (JVM). This function must be called before any RMut functions can be used.

Usage

```
initJVM(maxHeapSize)
```

Arguments

`maxHeapSize`	The maximum Java heap size. Default is "1G" (means 1 gigabytes).

Details

This function initializes the JVM with a parameter of the maximum Java heap size `maxHeapSize`. The parameter is a string composed of a number and followed by a letter K, or M, or G (K indicates kilobytes, M indicates megabytes, G indicates gigabytes).

Value

TRUE denotes successful initialization, and FALSE indicates failure.

See Also

`setOpencl`, `showOpencl`

Examples

```
initJVM("1G")
```

`loadNetwork` *Loads a network from a file.*

Description

`loadNetwork` loads a network from a file and returns the network object.

Usage

```
loadNetwork(pathToFile)
```

Arguments

`pathToFile`	The path points to a file

Details

This function loads a network from a Tab-separated values text file and returns the network object. The file format contains three columns: source, interaction type, and target. "Source" and "target" are gene/protein identifiers that are used to define nodes, while "interaction type" labels the edges connecting each pair of nodes. The returned network object contains the network name, three data frames used for storing the nodes/edges and network attributes, respectively.

Value

The network object

See Also

`output`, `createRBNs`, `calSensitivity`

Examples

```
amrn <- loadNetwork("D:\\AMRN.sif")
print(amrn)
```

output — *Exports all node/edge/network attributes of a network or a set of networks.*

Description

`output` writes all node/edge/network attributes of a network or a set of networks into CSV files.

Usage

```
output(networks)
```

Arguments

`networks`	The network or the set of networks

Details

This function writes all node/edge/network attributes of a network or a set of networks into CSV files. For each network, the function exports all data frames of the network object containing structural attributes of the nodes/edges/network and sensitivity values of mutated groups.

The CSV files were outputed with names as follows: `[network name]`**_out_**`[data-frame name]`**.csv**. The structure of these networks are also exported as Tab-separated values text files (.SIF extension).

See Also

`loadNetwork`, `createRBNs`

Examples

```
data(amrn)
# Generate all possible initial-states each containing 10 Boolean nodes
set1 <- generateStates(10, "all")

# Generate all possible groups each containing a single node in the AMRN network
amrn <- generateGroups(amrn, "all", 1, 0)
amrn <- calSensitivity(amrn, set1, "knockout")

output(amrn)
```

perturb — *Perturb a set of node/edge groups.*

Description

Perturb a set of node/edge groups in a network.

Usage

```
perturb(network, groupSet, mutateMethod = "rule flip")
```

Arguments

`network`	The network contains the node/edge groups
`groupSet`	The indexing number of node/edge groups in the network
`mutateMethod`	The method of mutation to be performed, default is "rule flip"

Details

This function perturbs a set of node/edge groups in a network. Two parameters `groupSet`, and `mutateMethod` have same meaning as in the `calSensitivity` function.

Value

None. Error messages or information would be outputed to the screen.

See Also

`restore`, `generateGroups`, `generateGroup`, `calSensitivity`, `findAttractors`

Examples

```
data(amrn)
# Generate a group of two nodes and two edges in the AMRN network
amrn <- generateGroup(amrn, nodes = "AG, SUP", edges = "UFO (1) PI, LUG (-1) PI")
print(amrn$Group_1)

# Generate a specific initial-state for the AMRN network
state1 <- generateState(amrn, "1110011011")

# Find the original transition network (before making perturbations)
transNet <- findAttractors(amrn, state1)
print(transNet)

# Perturb the group with overexpression mutation,
#  in this case only two nodes (AG, SUP) of the group are affected by the mutation.
perturb(amrn, 1, "overexpression")

# Continuously perturb the group with edge-removal mutation,
#  in this case only two edges of the group are removed by the mutation.
perturb(amrn, 1, "edge removal")

# Continuously perturb the group with "state-flip" mutation,
#  thus only two nodes (AG, SUP) of the group are affected by the mutation.
perturb(amrn, 1, "state flip")

# Find the perturbed transition network
perturbed_transNet <- findAttractors(amrn, state1)
print(perturbed_transNet)
```

printSensitivity *Print out the sensitivity values of node/edge groups*

Description

Print out the sensitivity values of node/edge groups in a network

Usage

```
printSensitivity(network, groupSet = 0)
```

Arguments

network	A network used for the outputting
groupSet	The indexing number of node/edge groups for whose sensitivity values are calculated. Default is 0 which specify the latest generated groups.

Details

This function prints out the sensitivity values of node/edge groups in a specific network. And the parameter groupSet has same meaning as in the calSensitivity function.

Value

None

See Also

calSensitivity, generateStates, generateGroups, generateGroup, findFBLs, findFFLs, calCentrality, findAttractors

Examples

```
data(amrn)

# generate 1000 random initial-states
states <- generateStates(amrn, 1000)
print(states)

# generate all possible groups each containing a single node in the HSN network
amrn <- generateGroups(amrn, "all", 1, 0)

# calculate sensitivity values of all nodes against the knockout mutation
amrn <- calSensitivity(amrn, states, "knockout")

# view the calculated sensitivity values and export all results to files
printSensitivity(amrn)
```

`restore` *Restore a set of node/edge groups.*

Description

Restore a set of node/edge groups in a network.

Usage

```
restore(network, groupSet)
```

Arguments

`network`	The network contains the node/edge groups
`groupSet`	The indexing number of node/edge groups in the network

Details

This function restores a set of node/edge groups in a network to its normal condition. And the parameter `groupSet` has same meaning as in the `calSensitivity` function.

Value

None. Error messages or information would be outputed to the screen.

See Also

`perturb`, `generateGroups`, `generateGroup`, `calSensitivity`, `findAttractors`

Examples

```
data(amrn)
# Generate a group of two nodes in the AMRN network
amrn <- generateGroup(amrn, nodes = "AG, SUP")
print(amrn$Group_1)

# Generate a specific initial-state for the AMRN network
state1 <- generateState(amrn, "1110011011")

# Find the original transition network (before making perturbations)
transNet <- findAttractors(amrn, state1)
print(transNet)

# Perturb the group with overexpression mutation
perturb(amrn, 1, "overexpression")

# Continuously perturb the group with "state-flip" mutation
perturb(amrn, 1, "state flip")

# Find the perturbed transition network
perturbed_transNet <- findAttractors(amrn, state1)
print(perturbed_transNet)
```

```
# Restore the group from previous mutations
restore(amrn, 1)

# Find again the original transition network, it should be same with the "transNet" network
origin_transNet <- findAttractors(amrn, state1)
print(origin_transNet)
```

`setOpencl` *Enables or disables OpenCL computation.*

Description

`setOpencl` enables or disables OpenCL computation.

Usage

```
setOpencl(deviceType)
```

Arguments

`deviceType`	The type of OpenCL device, including three options: \'none\', \'cpu\' and \'gpu\'

Details

This function enables OpenCL computation by selecting a CPU/GPU device and utilizing all of its cores for further computation. Thus, all tasks will be executed in parallel. About the parameter `deviceType`, there exists three options: \'none\' means disable OpenCL, \'cpu\' means selecting a CPU device and \'gpu\' means using a GPU device.

Value

Information of the successfully selected device

See Also

`showOpencl`

Examples

```
showOpencl()
setOpencl("gpu")
setOpencl("cpu")
setOpencl("none")
```

`showOpencl` *Shows OpenCL information.*

Description

`showOpencl` gets OpenCL information and prints them to the console screen.

Usage

```
showOpencl()
```

Details

This function gets OpenCL information and prints them to the console screen. For ex., installed OpenCL platforms and its corresponding CPU/GPU devices.

Value

A string of OpenCL information

See Also

`setOpencl`

Examples

```
showOpencl()
```

Index

∗Topic **datasets**
amrn, 5
cchs, 8
ccsn, 9
cdrn, 9
hsn, 18

amrn, 5

calCentrality, *3*, 6, *8*, *11–13*, *22*
calSensitivity, *3*, *6*, 7, *11–15*, *17*, *19*, *21–23*
cchs, 8
ccsn, 9
cdrn, 9
createRBNs, *2*, 10, *19*, *20*

data, *2*

findAttractors, *2*, *8*, 11, *16*, *17*, *21–23*
findFBLs, *3*, *6*, *8*, *11*, 12, *13*, *22*
findFFLs, *3*, *6*, *8*, *11*, *12*, 13, *22*

generateGroup, *8*, *11*, 14, *21–23*
generateGroups, *8*, *11*, 14, *21–23*
generateRule, *12*, 15
generateState, *8*, *11*, *12*, 16
generateStates, *8*, *11*, *12*, 17, *22*

hsn, 18

initJVM, 18

loadNetwork, *2*, *11*, 19, *20*

output, *2*, *19*, 20

perturb, *2*, *14–16*, 20, *23*
printSensitivity, 22

restore, *2*, *14–16*, *21*, 23
RMut-package, 2

setOpencl, *2*, *19*, 24, *25*
showOpencl, *2*, *19*, *24*, 25
