# Supplementary material for: RMut: R package for a Boolean sensitivity analysis against various types of mutations
Source: PLoS One. 2019 Mar 19;14(3):e0213736. doi: 10.1371/journal.pone.0213736 (PMC6424452; doi:10.1371/journal.pone.0213736)
Supplement: S3 File — (PDF 1,002 KB). (PDF) [file pone.0213736.s003.pdf]

RMut: R package for Boolean sensitivity analysis about various types of mutations

Hung-Cuong Trinh, Yung-Keun Kwon

2019-02-11

Contents

1 Setup guide **2**
1.1 Java SE Development Kit 2
1.2 RMut package 2
1.3 OpenCL library 2

2 Loading networks **4**
2.1 *loadNetwork* function 4
2.2 *data* function 5
2.3 WikiPathways network files conversion 6

3 Dynamics analyses **8**
3.1 Sensitivity analyses 8
3.2 Attractor cycles identification 15

4 Structural characteristics computation **17**
4.1 Feedback/Feed-forward loops search 17
4.2 Centrality measures computation 23

5 Export results **24**

6 Batch-mode analysis **24**

7 References **34**

1 Setup guide

To run and utilize all functions of *RMut* package, three following installations should be conducted in sequence:

1.1 Java SE Development Kit

Core algorithms of *RMut* package were written in Java, thus a Java SE Development Kit (JDK) is required to run the package. The JDK is available at:

http://www.oracle.com/technetwork/java/javase/downloads/index.html.

Two following kinds of JDK can be used alternatively:

- Old series

  Java SE 8u201 / Java SE 8u202 or higher version

- New series

  Java SE 11.0.2(LTS) or higher version

1.2 RMut package

Firstly, the *devtools* package must be installed by typing the following commands into the R console:

> *install.packages(“devtools”)*

More details about the *devtools* package could be found in the website https://github.com/r-lib/devtools.

Make sure you have Java Development Kit installed and correctly registered in R. If in doubt, run the command *R CMD javareconf* as root or administrator permission.

Next, the *RMut* package should be properly installed into the R environment by typing the following commands:

> *install.packages(“rJava”)*

> *devtools::install_github(“csclab/RMut”, INSTALL_opts=“–no-multiarch”)*

We note that the new version of *devtools* package uses the keyword *INSTALL_opts* to specify additional installation options instead of the old keyword *args*. Though all of core algorithms written in Java, the *rJava* package must be installed in the R environment before the *RMut* installation. After installation, the RMut package can be loaded via

> *library(RMut)*

In addition, we must initialize the Java Virtual Machine (JVM) with a *Maximum Java heap size* via the function *initJVM*. This function must be called before any RMut functions can be used. The following command will initialize the JVM with the maximum Java heap size of 8GB (in case of large-scale networks analysis, we could set the Java heap size to a larger value):

> *initJVM(“8G”)*

1.3 OpenCL library

In order to utilize the full computing power of multi-core central processing units (CPUs) and graphics processing units (GPUs), OpenCL drivers should be installed into your system. Here are necessary steps for a system with:

- NVIDIA graphics cards

  OpenCL support is included in the latest drivers, in the driver CD or available at

  https://www.nvidia.com/Download/index.aspx?lang=en-us

- AMD graphics cards

  The OpenCL GPU runtime library is included in the drivers of your AMD cards. The drivers could be in the driver CD or available at

  https://www.amd.com/en/support

- CPU devices only (No graphics cards)

  At the time of developing this R package, CPU devices from AMD are no longer supported as OpenCL device. For Intel CPU devices, the OpenCL runtime library is available at:

  https://software.intel.com/en-us/articles/opencl-drivers

After installation, OpenCL information can be outputed via the function *showOpencl*. Then we can enable OpenCL computation on a CPU/GPU device via the function *setOpencl*:

```
library(RMut)
```

```
## Loading required package: rJava
```

```
## [1] "Please firstly initialize the Java Virtual Machine by using the function 'initJVM(maxHeapSize)'
```

```
initJVM("8G")
```

```
## [1] "The Java Virtual Machine is successfully initialized!"
```

```
## [1] TRUE
```

```
showOpencl()
```

```
## Your system has 1 installed OpenCL platform(s):
## 1. Intel(R) OpenCL
##   PROFILE = FULL_PROFILE
##   VERSION = OpenCL 2.1
##   VENDOR = Intel(R) Corporation
##   EXTENSIONS = cl_intel_dx9_media_sharing cl_khr_3d_image_writes cl_khr_byte_addressable_store cl_kh
##  1 CPU device(s) found on the platform:
##  1. Intel(R) Core(TM) i3-6006U CPU @ 2.00GHz
##  DEVICE_VENDOR = Intel(R) Corporation
##  DEVICE_VERSION = OpenCL 1.2 (Build 611)
##  CL_DEVICE_MAX_COMPUTE_UNITS: 4
##  1 GPU device(s) found on the platform:
##  1. Intel(R) HD Graphics 520
##  DEVICE_VENDOR = Intel(R) Corporation
##  DEVICE_VERSION = OpenCL 2.1 NEO
##  CL_DEVICE_MAX_COMPUTE_UNITS: 23
```

```
setOpencl("gpu")
```

```
## Enabled OpenCL computation based on the device: Intel(R) HD Graphics 520.
```

The above functions show installed OpenCL platforms with their corresponding CPU/GPU devices, and try to select an graphics card for OpenCL computing.

2 Loading networks

Networks can be loaded in two ways using RMut:

2.1 *loadNetwork* function

The *loadNetwork* function creates a network from a Tab-separated values text file. The file format contains three columns:

- *source* and *target*: are gene/protein identifiers that are used to define nodes
- *interaction type*: labels the edges connecting each pair of nodes

The function returned a network object which contains:

- The network name
- Three data frames used for storing attributes of the nodes/edges and the network itself, respectively

Here is an example:

```
amrn <- loadNetwork("networks/AMRN.sif")
print(amrn)
```

```
## $name
## [1] "AMRN.sif"
##
## $nodes
##    NodeID
## 1      AG
## 2     AP1
## 3     AP3
## 4    EMF1
## 5     LFY
## 6     LUG
## 7      PI
## 8     SUP
## 9    TFL1
## 10    UFO
##
## $edges
##           EdgeID
## 1    AG (-1) AP1
## 2    AP1 (-1) AG
## 3    AP1 (1) LFY
## 4    AP3 (1) AP3
## 5     AP3 (1) PI
## 6  EMF1 (-1) AP1
## 7  EMF1 (-1) LFY
## 8  EMF1 (1) TFL1
## 9  LFY (-1) TFL1
## 10    LFY (1) AG
## 11   LFY (1) AP1
## 12   LFY (1) AP3
## 13    LFY (1) PI
## 14   LUG (-1) AG
## 15    PI (1) AP3
```

```
## 16     PI (1) PI
## 17  SUP (-1) AP3
## 18   SUP (-1) PI
## 19  TFL1 (-1) AG
## 20 TFL1 (-1) LFY
## 21   UFO (1) AP3
## 22    UFO (1) PI
##
## $network
##   NetworkID
## 1  AMRN.sif
##
## $transitionNetwork
## [1] FALSE
##
## attr(,"class")
## [1] "list"    "NetInfo"
```

Finally, the loaded network object *amrn* has five components:

- *name*: a string variable represents the network identifier, *AMRN.sif* in this case.
- *nodes*: a data frame which initially contains one column for node identifiers.

  In this example network, there exists 10 nodes. Additional columns for other node-based attributes would be inserted later.
- *edges*: a data frame which initially contains one column for edge identifiers.

  In this example, there exists 22 edges. Additional columns for other edge-based attributes would be inserted later.
- *network*: a data frame which initially contains one column for the network identifier (*AMRN.sif* in this case).

  Additional columns for other network-based attributes would be inserted later, such as total number of feedback/feed-forward loops.
- *transitionNetwork*: a Boolean variable denotes whether the network is a transition network or not, in this case the value is *FALSE*.

  The *findAttractors* function returns a transition network object in which the *transitionNetwork* variable has a value *TRUE*. For all other cases, the variable has a value *FALSE*.

2.2 *data* function

In addition, the package provides some example networks that could be simply loaded by *data* command. For ex.,

```
data(amrn)
```

The package supplied four example datasets from small-scale to large-scale real biological networks:

- *amrn*

  The Arabidopsis morphogenesis regulatory network (AMRN) with 10 nodes and 22 links.
- *cdrn*

  The cell differentiation regulatory network (CDRN) with 9 nodes and 15 links.

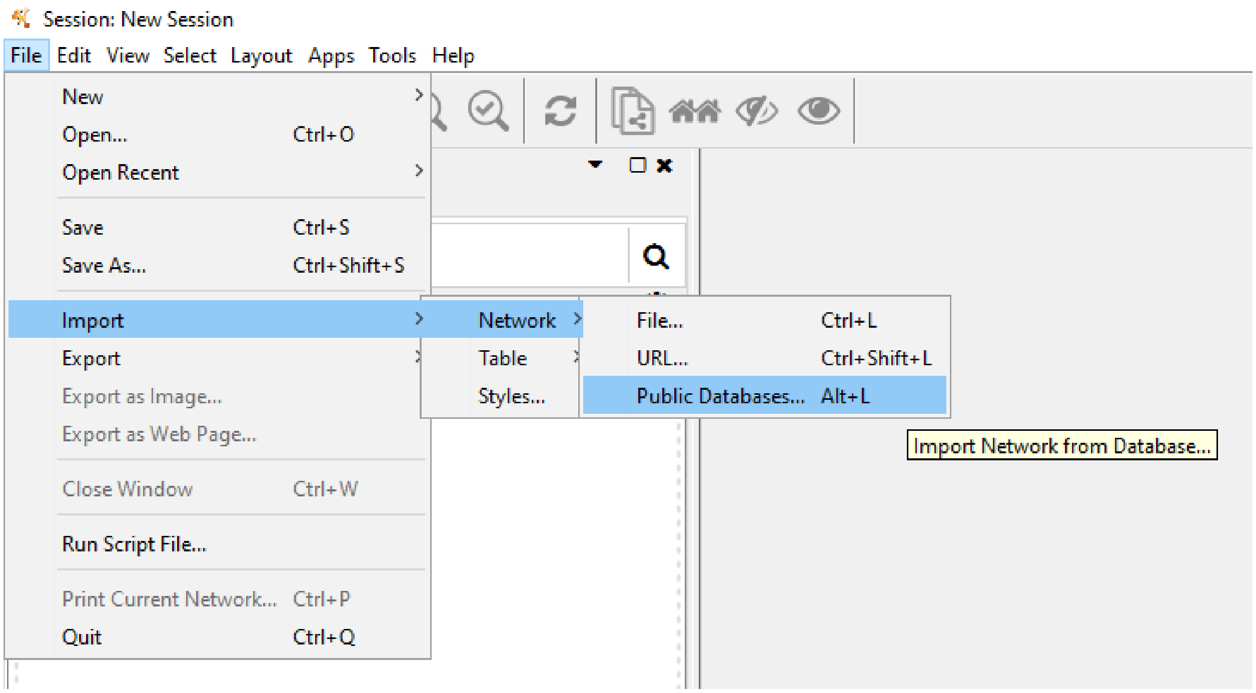

Figure 1: Import network from public databases

- *cchs*

  The cell cycle pathway of the species Homo sapiens (CCHS) with 161 nodes and 223 links.

- *ccsn*

  The canonical cell signaling network (CCSN) with 771 nodes and 1633 links.

- *hsn*

  The large-scale human signaling network (HSN) with 1192 nodes and 3102 links.

All original network files (Tab-separated values text files) could be downloaded in the folder *vignettes/networks* of the RMut website https://github.com/csclab/RMut.

2.3 WikiPathways network files conversion

A user could retrieve pathways in WikiPathways database (https://www.wikipathways.org) as a SIF file by the wikiPathways plugin of the Cytoscape software. The version of Cytoscape should be greater than or equal 3.6.1.

Firstly, the pathway could be loaded into Cytoscape by some steps indicated in the Figure 1 and 2.

After that, we select the "Edge Table" tab and detach it for easy modification (Figure 3).

There does not exist relationship types in the attribute or column *interaction* (activation, inhibition, or neutral), thus we must update them based on some existing columns as follows:

- *activation* interaction (value is *1*)

  In case at least one of the corresponding columns *WP.type* or *Source Arrow Shape* has the value "mim-conversion" or "Arrow".

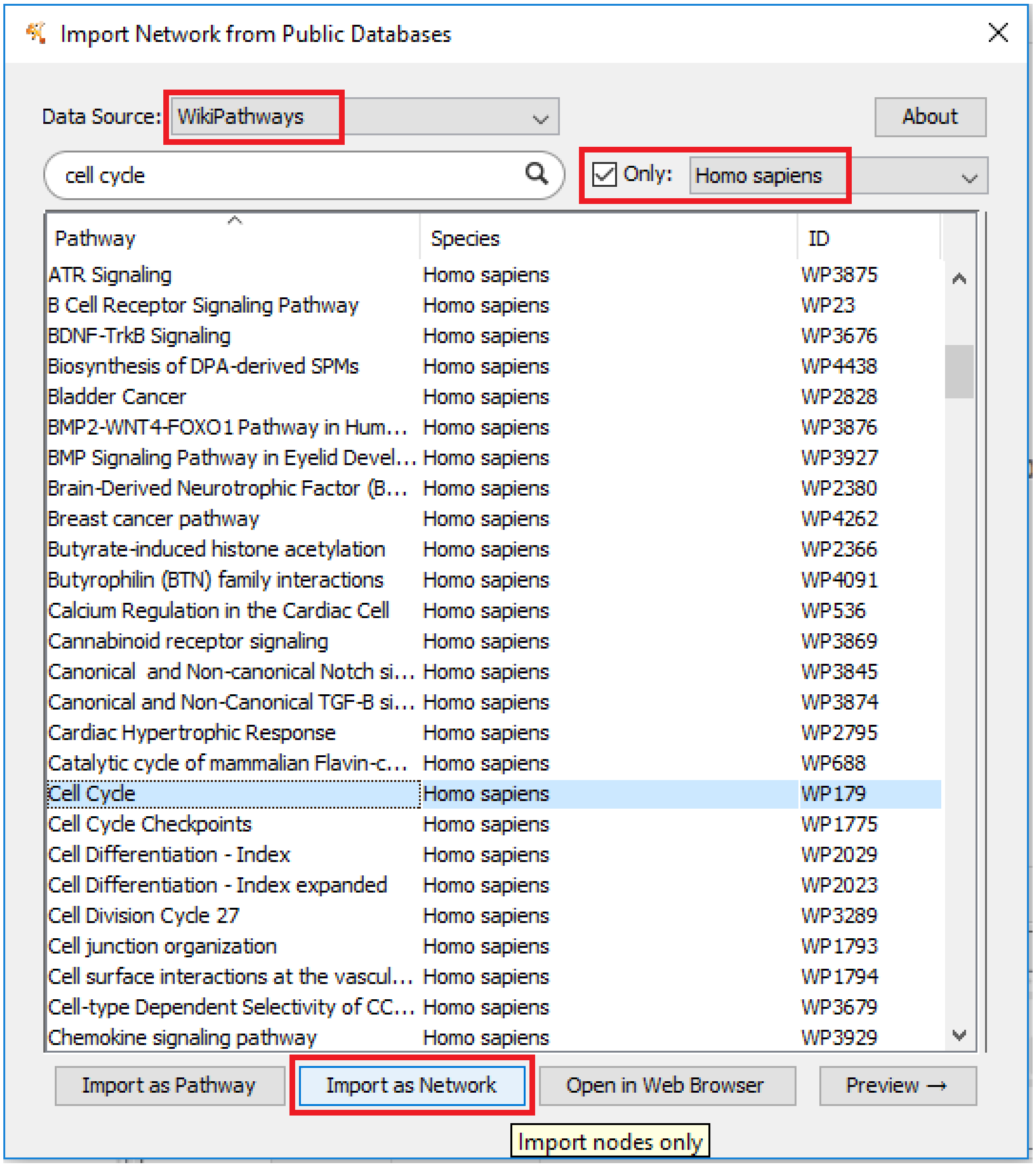

Figure 2: Select and import a pathway from WikiPathways database

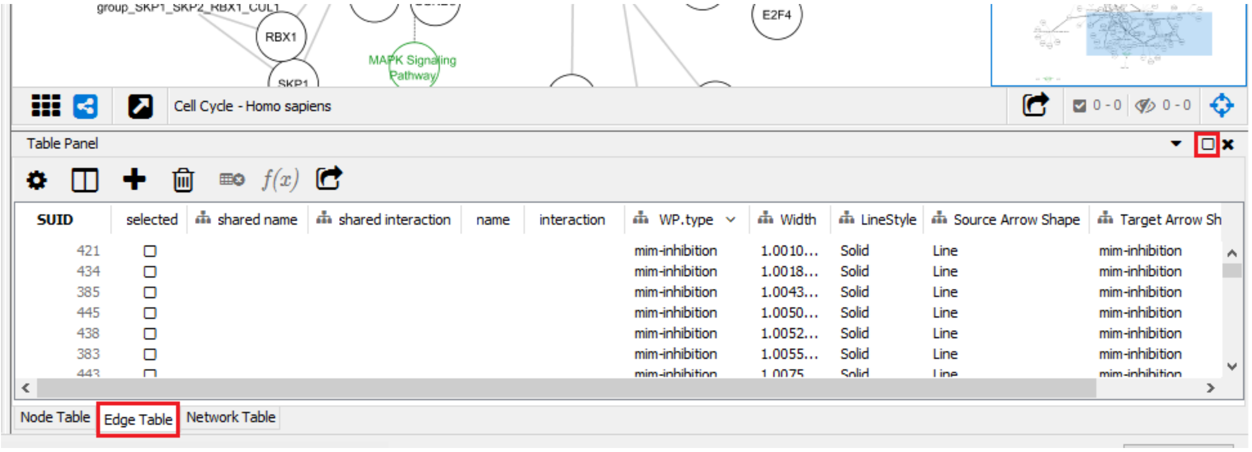

Figure 3: Select and detach the "Edge Table" tab

- *inhibition* interaction (value is *-1*)

  In case at least one of the corresponding columns *WP.type* or *Source Arrow Shape* has the value "mim-inhibition" or "TBar".

- *neutral* interaction (value is *0*)

  In case both the corresponding columns *WP.type* and *Source Arrow Shape* has the value "Line", or the corresponding column *WP.type* is empty.

For each type of interaction, we select the rows or interactions that satisfy the above conditions, and then modify the values of the column *interaction* as a way like Figure 4.

To repeat this step for other types, we deselect edges by clicking in the empty space of the network visualization panel. Finally, we export the pathway to SIF file format by the following menu: *File / Export / Network...* . We might need to remove wrong rows of interactions (missing the interaction type) in the SIF file by a spreadsheet software like Microsoft Excel (Figure 5).

3 Dynamics analyses

The package utilizes a Boolean network model with synchronous updating scheme, and provides two types of useful analyses of Boolean dynamics in real biological networks or random networks:

3.1 Sensitivity analyses

Via *calSensitivity* function, this package computes nodal/edgetic sensitivity against many types of mutations in terms of Boolean dynamics. We classified ten well-known mutations into two types (refer to RMut paper for more details):

- *Node-based* mutations: state-flip, rule-flip, outcome-shuffle, knockout and overexpression
- *Edgetic* mutations: edge-removal, edge-attenuation, edge-addition, edge-sign-switch, and edge-reverse

Two kinds of sensitivity measures are computed: macro-distance and bitwise-distance sensitivity measures. In addition, we note that multiple sets of random Nested Canalyzing rules could be specified, and thus resulted in multiple sensitivity values for each node/edge. Here, we show an example of some sensitivity types:

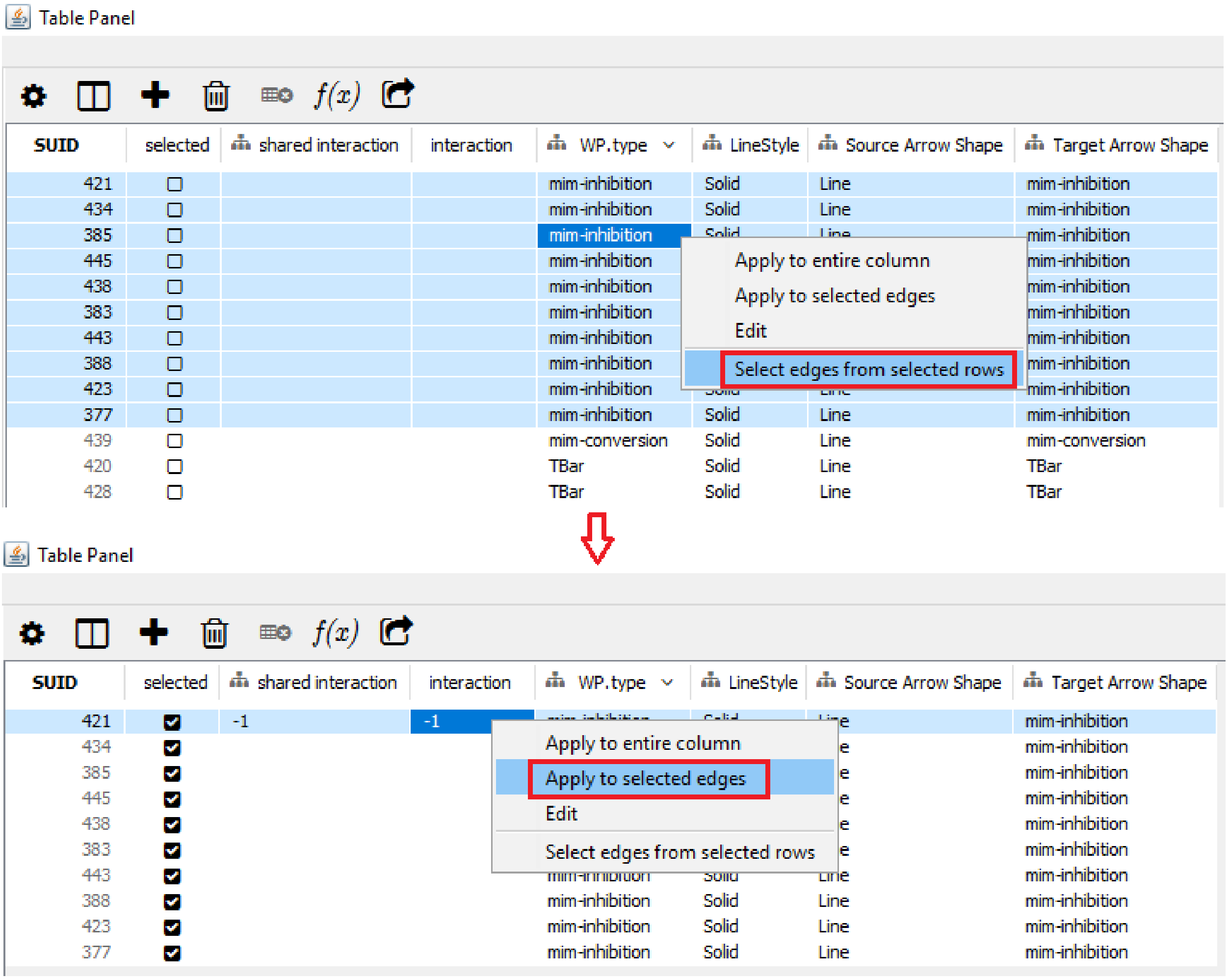

Figure 4: Update relationship types in the attribute "interaction"

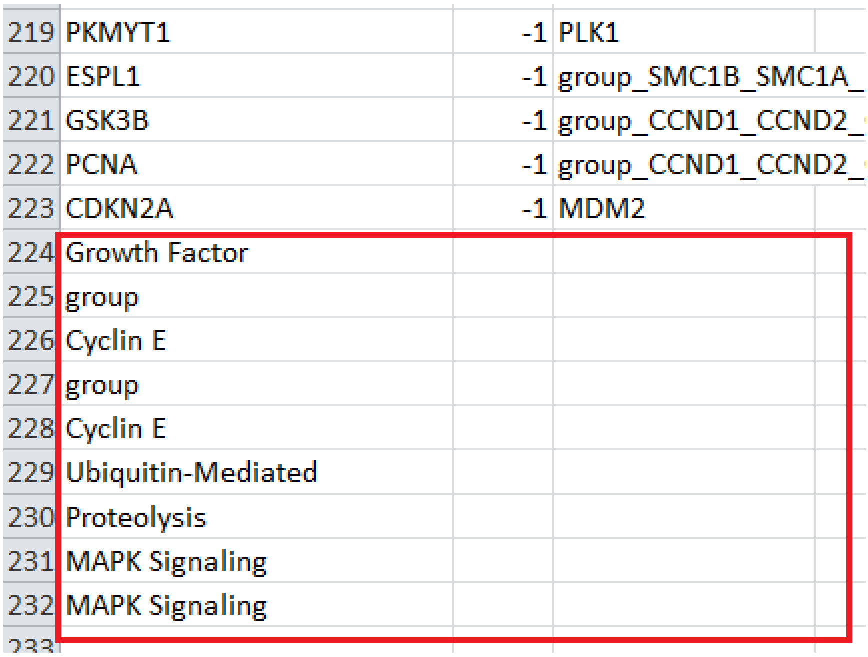

219	PKMYT1	-1	PLK1
220	ESPL1	-1	group_SMC1B_SMC1A_
221	GSK3B	-1	group_CCND1_CCND2_
222	PCNA	-1	group_CCND1_CCND2_
223	CDKN2A	-1	MDM2
224	Growth Factor		
225	group		
226	Cyclin E		
227	group		
228	Cyclin E		
229	Ubiquitin-Mediated		
230	Proteolysis		
231	MAPK Signaling		
232	MAPK Signaling		

Figure 5: Remove wrong rows of interactions which have not an interaction type)

```r
data(amrn)

# generate all possible initial-states each containing 10 Boolean nodes
set1 <- generateStates(10, "all")

# generate all possible groups each containing a single node in the AMRN network
amrn <- generateGroups(amrn, "all", 1, 0)
```

```
## [1] "Number of possibly mutated groups:10"
```

```r
amrn <- calSensitivity(amrn, set1, "rule flip", numRuleSets = 2)
print(amrn$Group_1)
```

```
##    GroupID ruleflip_t1000_r1_macro ruleflip_t1000_r1_bitws
## 1       AG               1.0000000              0.12262370
## 2       PI               0.7988281              0.10511068
## 3      AP3               0.7617188              0.08886719
## 4     TFL1               0.4687500              0.05335286
## 5      SUP               0.0000000              0.00000000
## 6     EMF1               0.0000000              0.00000000
## 7      LFY               0.9062500              0.16064453
## 8      AP1               0.9687500              0.13518880
## 9      UFO               0.0000000              0.00000000
## 10     LUG               0.0000000              0.00000000
##    ruleflip_t1000_r2_macro ruleflip_t1000_r2_bitws
## 1                1.0000000              0.12177734
## 2                0.9707031              0.10488281
## 3                0.9707031              0.10488281
## 4                0.4687500              0.05458984
## 5                0.0000000              0.00000000
```

```
## 6                  0.0000000              0.00000000
## 7                  0.9062500              0.14690755
## 8                  0.9687500              0.12900391
## 9                  0.0000000              0.00000000
## 10                 0.0000000              0.00000000
```

```
# generate all possible groups each containing a single edge in the AMRN network
amrn <- generateGroups(amrn, "all", 0, 1)
```

```
## [1] "Number of possibly mutated groups:22"
```

```
amrn <- calSensitivity(amrn, set1, "edge removal")
print(amrn$Group_2)
```

```
##          GroupID edgeremoval_t1000_r1_macro edgeremoval_t1000_r1_bitws
## 1  TFL1 (-1) LFY                 0.12500000                0.015755208
## 2     UFO (1) PI                 0.01757812                0.003222656
## 3    LFY (1) AP3                 0.00390625                0.000390625
## 4  EMF1 (-1) AP1                 0.00000000                0.000000000
## 5    AG (-1) AP1                 0.12500000                0.016178385
## 6    UFO (1) AP3                 0.01562500                0.003710938
## 7      PI (1) PI                 0.00390625                0.000390625
## 8    AP1 (-1) AG                 0.03125000                0.005794271
## 9   TFL1 (-1) AG                 0.01269531                0.003808594
## 10   AP3 (1) AP3                 0.00000000                0.000000000
## 11    PI (1) AP3                 0.18945312                0.026269531
## 12 LFY (-1) TFL1                 0.00000000                0.000000000
## 13    AP3 (1) PI                 0.02539062                0.006152344
## 14   AP1 (1) LFY                 0.46875000                0.075358073
## 15  SUP (-1) AP3                 0.01562500                0.003710938
## 16   LFY (1) AP1                 0.42187500                0.074804688
## 17   LUG (-1) AG                 0.09375000                0.010188802
## 18    LFY (1) AG                 0.14062500                0.014062500
## 19   SUP (-1) PI                 0.01757812                0.003222656
## 20 EMF1 (1) TFL1                 0.46875000                0.053352865
## 21 EMF1 (-1) LFY                 0.00000000                0.000000000
## 22    LFY (1) PI                 0.18164062                0.034375000
```

```
# generate all possible groups each containing a new edge (not exist in the AMRN network)
amrn <- generateGroups(amrn, "all", 0, 1, TRUE)
```

```
## [1] "Number of possibly mutated groups:178"
```

```
amrn <- calSensitivity(amrn, set1, "edge addition")
print(amrn$Group_3)
```

```
##            GroupID edgeaddition_t1000_r1_macro edgeaddition_t1000_r1_bitws
## 1      AP1 (1) AP3                 0.987304688                 0.353027344
## 2      LFY (1) LFY                 0.062500000                 0.008463542
## 3        PI (1) AG                 0.996093750                 0.120670573
## 4      AP1 (1) AP1                 0.062500000                 0.008463542
## 5     EMF1 (1) UFO                 0.500000000                 0.051953125
## 6     AP1 (-1) SUP                 0.593750000                 0.059505208
## 7     AP1 (-1) LUG                 0.593750000                 0.062890625
## 8        AG (1) AP1                 0.000000000                 0.000000000
## 9     AP3 (-1) SUP                 0.537109375                 0.069531250
## 10    AP1 (-1) UFO                 0.593750000                 0.053938802
```

```
## 11    UFO (-1) LUG                0.500000000                0.060904948
## 12    SUP (-1) UFO                0.500000000                0.051953125
## 13     AP1 (1) SUP                0.593750000                0.059505208
## 14      AG (-1) AG                0.000000000                0.000000000
## 15      PI (-1) AG                0.113281250                0.018489583
## 16     LFY (-1) PI                1.000000000                0.472753906
## 17    AG (-1) TFL1                0.500000000                0.050000000
## 18    LUG (-1) AP3                0.987304688                0.353027344
## 19    AP1 (1) EMF1                0.468750000                0.116048177
## 20    LFY (1) EMF1                0.468750000                0.116048177
## 21    AP1 (-1) AP1                0.531250000                0.071126302
## 22      PI (1) LFY                0.083984375                0.011458333
## 23     PI (-1) SUP                0.552734375                0.074023438
## 24     UFO (1) LUG                0.500000000                0.061002604
## 25      AG (1) LUG                0.531250000                0.058886719
## 26    TFL1 (-1) PI                1.000000000                0.472753906
## 27   TFL1 (-1) LUG                0.500000000                0.060742188
## 28     SUP (1) AP1                0.109375000                0.015397135
## 29    LFY (-1) UFO                0.593750000                0.060286458
## 30    UFO (-1) SUP                0.500000000                0.055566406
## 31    LUG (-1) UFO                0.500000000                0.054003906
## 32    EMF1 (-1) AG                0.000000000                0.000000000
## 33   AP1 (-1) TFL1                0.468750000                0.069173177
## 34    SUP (-1) SUP                1.000000000                0.056770833
## 35     PI (1) EMF1                0.619140625                0.116731771
## 36    LUG (-1) SUP                0.500000000                0.054003906
## 37    LFY (-1) AP1                0.000000000                0.000000000
## 38     LUG (1) LFY                0.109375000                0.016634115
## 39     AP3 (1) LUG                0.517578125                0.062337240
## 40    AP3 (-1) UFO                0.539062500                0.070605469
## 41   LUG (-1) EMF1                0.500000000                0.116634115
## 42    AP1 (-1) AP3                0.987304688                0.353027344
## 43     LFY (1) LUG                0.593750000                0.062890625
## 44     LFY (-1) AG                0.968750000                0.112076823
## 45   TFL1 (-1) AP1                0.218750000                0.029720052
## 46   SUP (-1) EMF1                0.500000000                0.115397135
## 47    AP3 (1) EMF1                0.619140625                0.116731771
## 48    UFO (1) TFL1                0.250000000                0.025000000
## 49     AG (-1) LUG                0.593750000                0.073561198
## 50    AP3 (-1) LUG                0.505859375                0.062500000
## 51      SUP (1) PI                1.000000000                0.472753906
## 52    TFL1 (1) SUP                0.509765625                0.057031250
## 53    EMF1 (1) AP3                0.987304688                0.353027344
## 54     LUG (1) UFO                0.500000000                0.054003906
## 55    EMF1 (1) LUG                0.500000000                0.060742188
## 56     SUP (1) LFY                0.109375000                0.015397135
## 57  EMF1 (-1) EMF1                1.000000000                0.129720052
## 58   LUG (-1) TFL1                0.250000000                0.041634115
## 59   TFL1 (-1) SUP                0.515625000                0.062597656
## 60   AP3 (-1) EMF1                0.619140625                0.116731771
## 61  TFL1 (-1) TFL1                0.500000000                0.027799479
## 62   EMF1 (-1) AP3                0.987304688                0.353027344
## 63     AP3 (-1) PI                1.000000000                0.472753906
## 64    SUP (-1) LUG                0.500000000                0.060904948
```

```
## 65    LFY (-1) LFY                0.781250000                0.082812500
## 66     EMF1 (1) PI                1.000000000                0.472753906
## 67    SUP (-1) AP1                0.484375000                0.064322917
## 68   EMF1 (1) EMF1                0.000000000                0.000000000
## 69    LUG (1) TFL1                0.250000000                0.025000000
## 70    TFL1 (1) LUG                0.525390625                0.061165365
## 71      LUG (1) AG                0.093750000                0.010188802
## 72    AP3 (-1) LFY                0.216796875                0.028938802
## 73   EMF1 (-1) SUP                0.500000000                0.051953125
## 74     AP3 (1) SUP                0.539062500                0.070605469
## 75     AP1 (1) UFO                0.593750000                0.053938802
## 76    EMF1 (1) AP1                0.000000000                0.000000000
## 77     AG (-1) UFO                0.562500000                0.072493490
## 78     UFO (-1) AG                0.046875000                0.005729167
## 79      AG (1) AP3                0.987304688                0.353027344
## 80      AP3 (1) AG                0.998046875                0.121647135
## 81     AP3 (1) UFO                0.539062500                0.070605469
## 82    LFY (-1) SUP                0.593750000                0.060286458
## 83     EMF1 (1) AG                0.500000000                0.072623698
## 84     AP3 (-1) AG                0.005859375                0.001757813
## 85    TFL1 (1) UFO                0.515625000                0.062597656
## 86    AP1 (1) TFL1                0.468750000                0.069173177
## 87   AP3 (-1) TFL1                0.099609375                0.015104167
## 88    SUP (1) EMF1                0.500000000                0.115397135
## 89    AP3 (-1) AP3                0.987304688                0.353027344
## 90   LFY (-1) EMF1                0.718750000                0.129720052
## 91      AP1 (1) PI                1.000000000                0.449316406
## 92     PI (-1) AP3                0.987304688                0.353027344
## 93    LUG (-1) LUG                0.000000000                0.000000000
## 94    EMF1 (1) LFY                0.218750000                0.029720052
## 95      PI (1) UFO                0.535156250                0.073339844
## 96   TFL1 (-1) UFO                0.515625000                0.062597656
## 97     SUP (1) SUP                1.000000000                0.056770833
## 98   TFL1 (1) EMF1                1.000000000                0.127923177
## 99     PI (-1) AP1                0.070312500                0.010058594
## 100  EMF1 (-1) UFO                0.500000000                0.055566406
## 101  SUP (-1) TFL1                0.250000000                0.040397135
## 102     AG (-1) PI                0.992187500                0.454003906
## 103     UFO (1) AG                0.500000000                0.061946615
## 104   UFO (1) EMF1                0.500000000                0.115397135
## 105    SUP (1) UFO                0.500000000                0.055566406
## 106      AG (1) AG                0.578125000                0.066764323
## 107    UFO (1) UFO                0.000000000                0.000000000
## 108    TFL1 (1) AG                0.500000000                0.072623698
## 109     AG (1) SUP                0.562500000                0.072493490
## 110    PI (-1) LFY                0.214843750                0.028157552
## 111    LUG (1) LUG                0.000000000                0.000000000
## 112   EMF1 (-1) PI                1.000000000                0.472753906
## 113    AG (-1) SUP                0.562500000                0.072493490
## 114 TFL1 (-1) EMF1                1.000000000                0.127923177
## 115   TFL1 (1) AP3                0.987304688                0.353027344
## 116   LUG (1) EMF1                0.500000000                0.116634115
## 117     PI (1) SUP                0.552734375                0.074023438
## 118    UFO (1) AP1                0.109375000                0.014322917
```

```
## 119     PI (1) LUG              0.517578125           0.062337240
## 120   PI (-1) TFL1              0.496093750           0.077766927
## 121  EMF1 (-1) LUG              0.500000000           0.061165365
## 122     SUP (1) AG              0.500000000           0.061946615
## 123    AP3 (1) LFY              0.216796875           0.028938802
## 124   UFO (-1) AP3              0.987304688           0.353027344
## 125  TFL1 (1) TFL1              0.375000000           0.037500000
## 126   LUG (-1) AP1              0.109375000           0.013085937
## 127    LFY (1) SUP              0.593750000           0.060286458
## 128  UFO (-1) TFL1              0.250000000           0.025000000
## 129    UFO (1) SUP              0.500000000           0.051953125
## 130  TFL1 (-1) AP3              0.987304688           0.353027344
## 131     AP1 (1) AG              0.000000000           0.000000000
## 132   AP1 (-1) LFY              0.218750000           0.029720052
## 133  AP1 (-1) EMF1              0.468750000           0.116048177
## 134   EMF1 (1) SUP              0.500000000           0.051953125
## 135    LUG (1) AP1              0.484375000           0.065820313
## 136 EMF1 (-1) TFL1              0.500000000           0.050000000
## 137    AG (1) EMF1              0.500000000           0.129720052
## 138   TFL1 (1) LFY              0.000000000           0.000000000
## 139   AP3 (1) TFL1              0.500000000           0.050000000
## 140    SUP (1) AP3              0.987304688           0.353027344
## 141    LUG (-1) PI              1.000000000           0.472753906
## 142     AG (1) UFO              0.562500000           0.072623698
## 143    SUP (-1) AG              0.500000000           0.061946615
## 144   AG (-1) EMF1              0.625000000           0.116048177
## 145     LUG (1) PI              1.000000000           0.472753906
## 146    PI (1) TFL1              0.500000000           0.050000000
## 147    SUP (1) LUG              0.500000000           0.061002604
## 148   UFO (-1) AP1              0.484375000           0.064322917
## 149   PI (-1) EMF1              0.619140625           0.116731771
## 150    LUG (1) AP3              0.987304688           0.353027344
## 151   LFY (1) TFL1              0.218750000           0.029720052
## 152    AP1 (-1) PI              1.000000000           0.449316406
## 153   UFO (-1) LFY              0.484375000           0.121842448
## 154   LFY (-1) LUG              0.593750000           0.062988281
## 155    AG (-1) AP3              0.987304688           0.353027344
## 156      AG (1) PI              0.992187500           0.438509115
## 157     PI (-1) PI              1.000000000           0.472753906
## 158   AP3 (-1) AP1              0.080078125           0.011197917
## 159    LUG (1) SUP              0.500000000           0.053515625
## 160    AP3 (1) AP1              0.207031250           0.031770833
## 161    UFO (-1) PI              1.000000000           0.472753906
## 162     AG (1) LFY              0.171875000           0.023046875
## 163   LUG (-1) LFY              0.109375000           0.016634115
## 164    PI (-1) LUG              0.505859375           0.062500000
## 165    TFL1 (1) PI              1.000000000           0.472753906
## 166   UFO (-1) UFO              1.000000000           0.056770833
## 167    AP1 (1) LUG              0.593750000           0.062890625
## 168   LFY (-1) AP3              0.987304688           0.353027344
## 169   TFL1 (1) AP1              0.062500000           0.007291667
## 170    PI (-1) UFO              0.535156250           0.073339844
## 171    LFY (1) UFO              0.593750000           0.060286458
## 172    UFO (1) LFY              0.484375000           0.121842448
```

```
## 173    AG (-1) LFY                0.203125000                0.028776042
## 174  UFO (-1) EMF1               0.500000000                0.115397135
## 175   SUP (-1) LFY               0.484375000                0.121842448
## 176     PI (1) AP1               0.212890625                0.032649740
## 177    AG (1) TFL1               0.500000000                0.079720052
## 178   SUP (1) TFL1               0.250000000                0.025000000
```

As shown above, we firstly need to generate a set of initial-states by the function *generateStates*. Then by the function *generateGroups*, we continue to generate three sets of node/edge groups whose their sensitivity would be calculated. Finally, the sensitivity values are stored in the same data frame of node/edge groups. The data frame has one column for group identifiers (lists of nodes/edges), and some next columns containing their sensitivity values according to each set of random update-rules. For example, the mutation *rule-flip* used two sets of Nested Canalyzing rules, thus resulted in two corresponding sets of sensitivity values. RMut automatically generates a file of Boolean logics for each set, or uses existing files in the working directory of RMut. Here, two rule files "*AMRN_rules_0*" and "*AMRN_rules_1*" are generated. A user can manually create or modify these rule files before the calculation. In addition, the column names which contain the sequence "*macro*" or "*bitws*" denote the macro-distance and bitwise-distance sensitivity measures, respectively.

3.2 Attractor cycles identification

Via *findAttractors* function, the landscape of the network state transitions along with attractor cycles would be identified. The returned transition network object has same structures with the normal network object resulted from *loadNetwork* function (see section "*loadNetwork* function"). An example is demonstrated as follows:

```
data(amrn)

# generate all possible initial-states each containing 10 Boolean nodes
set1 <- generateStates(10, "all")

# generate a set of only conjunction rules
generateRule(amrn)
```

```
## [1] "Generate a default set of update-rules successfully!"

## [1] "ok"
```

```
transNet <- findAttractors(amrn, set1)
```

```
## [1] "Number of found attractors:34"
## [1] "Number of transition nodes:1024"
## [1] "Number of transition edges:1024"
```

```
# print some first network states
head(transNet$nodes)
```

```
##   NodeID Attractor NetworkState
## 1     N1         1   0000000000
## 2     N2         1   0000000001
## 3     N3         0   0000000010
## 4     N4         0   0000000011
## 5     N5         1   0000000100
## 6     N6         1   0000000101
```

```
# print some first transition links between network states
head(transNet$edges)
```

```
##      EdgeID Attractor
## 1 N1 (1) N1         1
## 2 N2 (1) N2         1
## 3 N3 (1) N1         0
## 4 N4 (1) N2         0
## 5 N5 (1) N5         1
## 6 N6 (1) N6         1
```

```r
output(transNet)
```

```
## [1] "All output files get created in the working directory:"
## [1] "D:/HCStore/Projects/R/RMut/vignettes"
```

As shown in the example, there exists some different points inside two nodes/edges's data frames of the *transNet* object compared to those of normal network objects:

- *nodes*:

  The first column is also used for node identifiers, but in this case they represent *states* of the analyzed network *amrn*. There exists 1024 nodes which are equivalent to 1024 network states of *amrn*.

  Additional columns are described as follows:

  - *Attractor*: value *1* denotes the network state belongs to an attractor, otherwises *0*.
  - *NetworkState*: specifies the network state of the node.

- *edges*:

  The first column is also used for edge identifiers, but in this case they represent *transition links* of the analyzed network *amrn*. Each edge identifier has a string *(1)* which denotes a directed link between two node identifiers. There exists 1024 edges which are equivalent to 1024 transition links of *amrn*.

  Additional columns are described as follows:

  - *Attractor*: value *1* means that the transition link connects two network states of an attractor, otherwises *0*.

We take the node *N6* as an example. Its corresponding network state is *0000000101* which represents Boolean values of all nodes in alphabetical order of the analyzed network *amrn*:

```
## [1] "Number of found FBLs:4"
## [1] "Number of found positive FBLs:4"
## [1] "Number of found negative FBLs:0"
```

```
## AG      AP1     AP3     EMF1    LFY     LUG     PI      SUP     TFL1    UFO

## 0       0       0       0       0       0       0       1       0       1
```

Moreover, the *Attractor* value *1* means that *N6* belongs to an attractor. And the data frame *edges* also shows a transition link *N6 (1) N6* with *Attractor* value 1. It means that *N6 (1) N6* is a fixed point attractor.

Finally, the resulted transition network could be exported by the function *output* (see section "*Export results*"). Three CSV files were outputed for the transition network itself and nodes/edges attributes with the following names: *AMRN_trans.sif*, *AMRN_trans_out_nodes.csv* and *AMRN_trans_out_edges.csv*, respectively. Then, those resulted files could be further loaded and analyzed by other softwares with powerful visualization functions like Cytoscape. For more information on Cytoscape, please refer to http://www.cytoscape.org/. In this tutorial, we used Cytoscape version 3.4.0.

The transition network is written as a SIF file (***.sif**). The SIF file could be loaded to Cytoscape with the following menu:

File | Import | Network | File... or using the shortcut keys *Ctrl/Cmd + L* (*Figure 6(a)*)

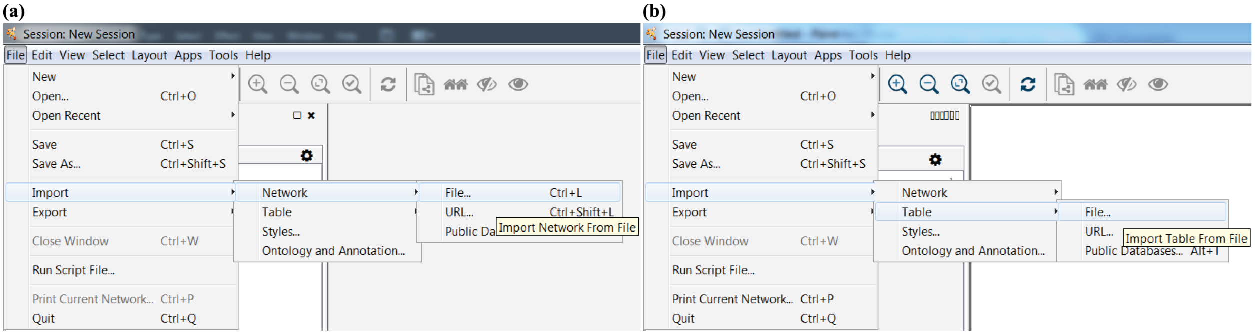

Figure 6: Import network (a) and nodes/edges attributes (b) in Cytoscape software

In next steps, we import two CSV files of nodes/edges attributes via *File | Import | Table | File...* menu (*Figure 6(b)*). For the nodes attributes file, we should select *String* data type for the column *NetworkState* (*Figure 7*). For the edges attributes file, we must select *Edge Table Columns* in the drop-down list beside the text *Import Data as:* (*Figure 8*).

After importing, we select *Style* panel and modify the node and edge styles a little to highlight all attractor cycles. For node style, select *Red* color in *Fill Color* property for the nodes that belong to an attractor (*Figure 9(a)*). Regards to edge style, select *Red* color in *Stroke Color* property and change *Width* property to a larger value (optional) for the edges that connect two states of an attractor (*Figure 9(b)*).

As a result, *Figure 10* shows the modified transition network with clearer indication of attractor cycles.

4 Structural characteristics computation

4.1 Feedback/Feed-forward loops search

Via *findFBLs* and *findFFLs*, the package supports methods of searching feedback/feed-forward loops (FBLs/FFLs), respectively, for all nodes/edges in a network. The following is an example R code for the search:

```
data(amrn)

# search feedback/feed-forward loops
amrn <- findFBLs(amrn, maxLength = 10)
```

```
## [1] "Number of found FBLs:6"
## [1] "Number of found positive FBLs:4"
## [1] "Number of found negative FBLs:2"
```

```
amrn <- findFFLs(amrn)
```

```
## [1] "Number of found FFLs:15"
## [1] "Number of found coherent FFLs:10"
## [1] "Number of found incoherent FFLs:5"
```

```
print(amrn$nodes)
```

```
##    NodeID NuFBL NuPosFBL NuNegFBL NuFFL NuFFL_A NuFFL_B NuFFL_C
## 1      AG     3        1        2     5       0       1       4
## 2     AP1     4        2        2     5       1       2       2
## 3     AP3     1        1        0     6       0       3       3
## 4    EMF1     0        0        0     4       4       0       0
```

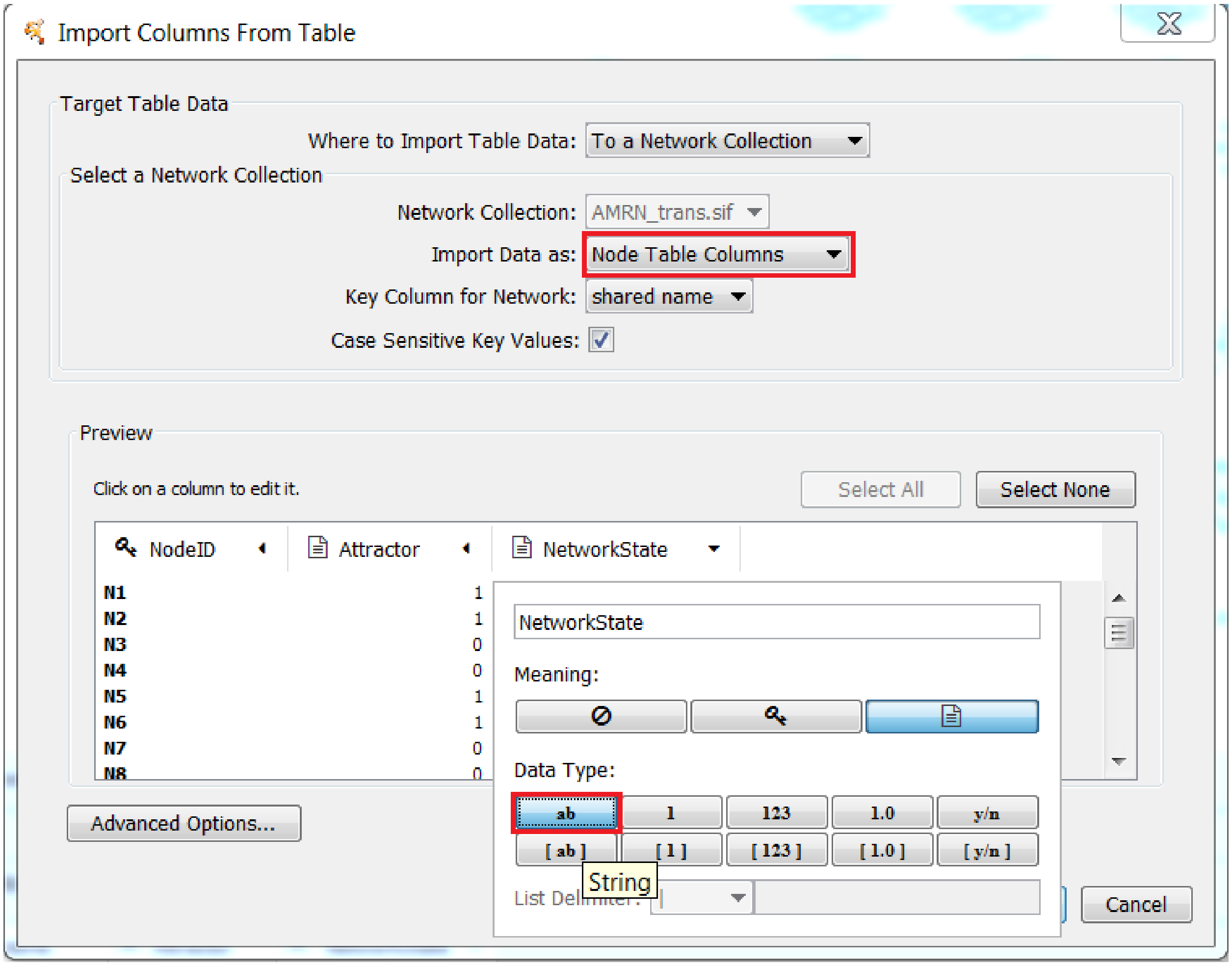

Figure 7: Nodes attributes importing dialog

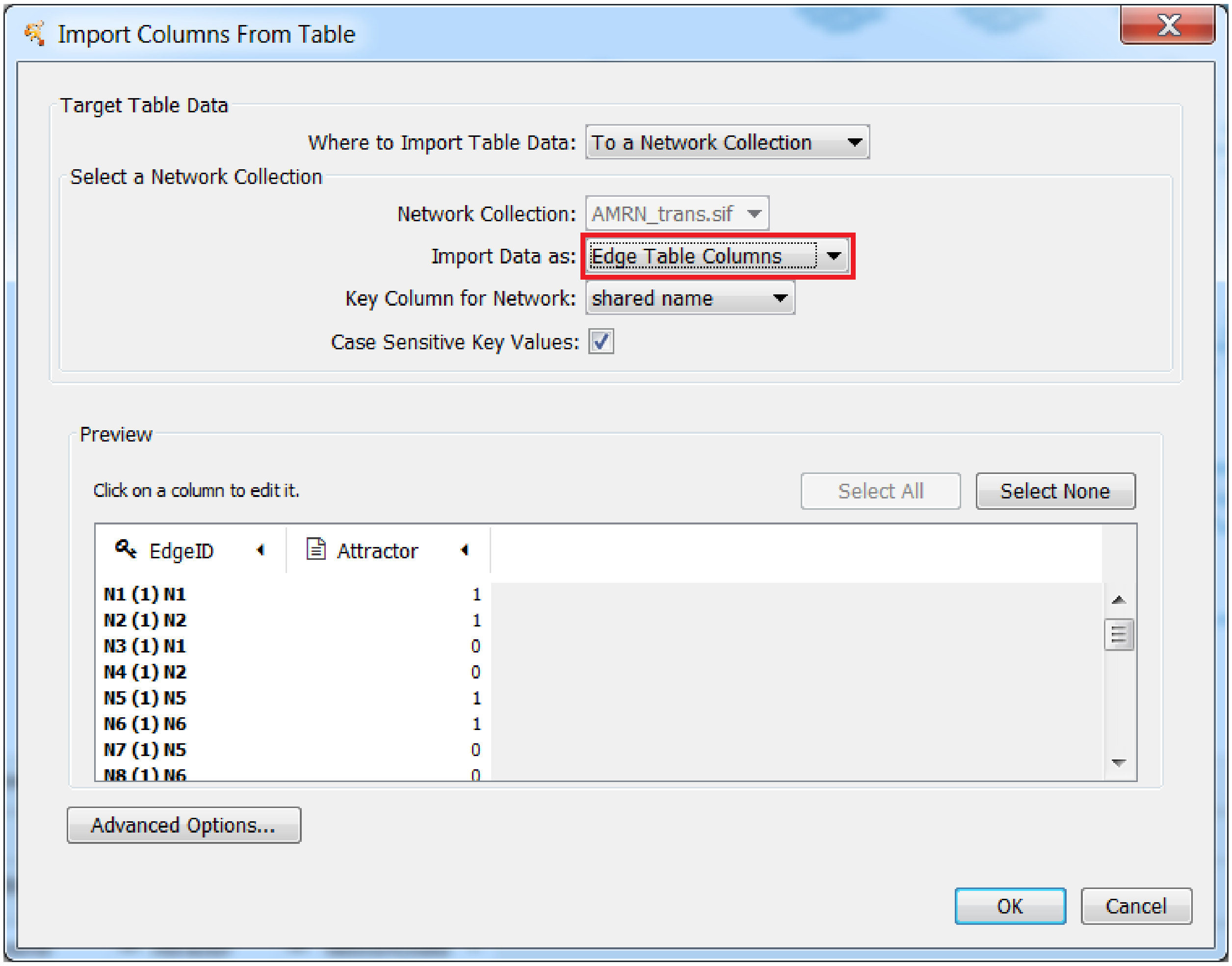

Figure 8: Edges attributes importing dialog

(a) (b)

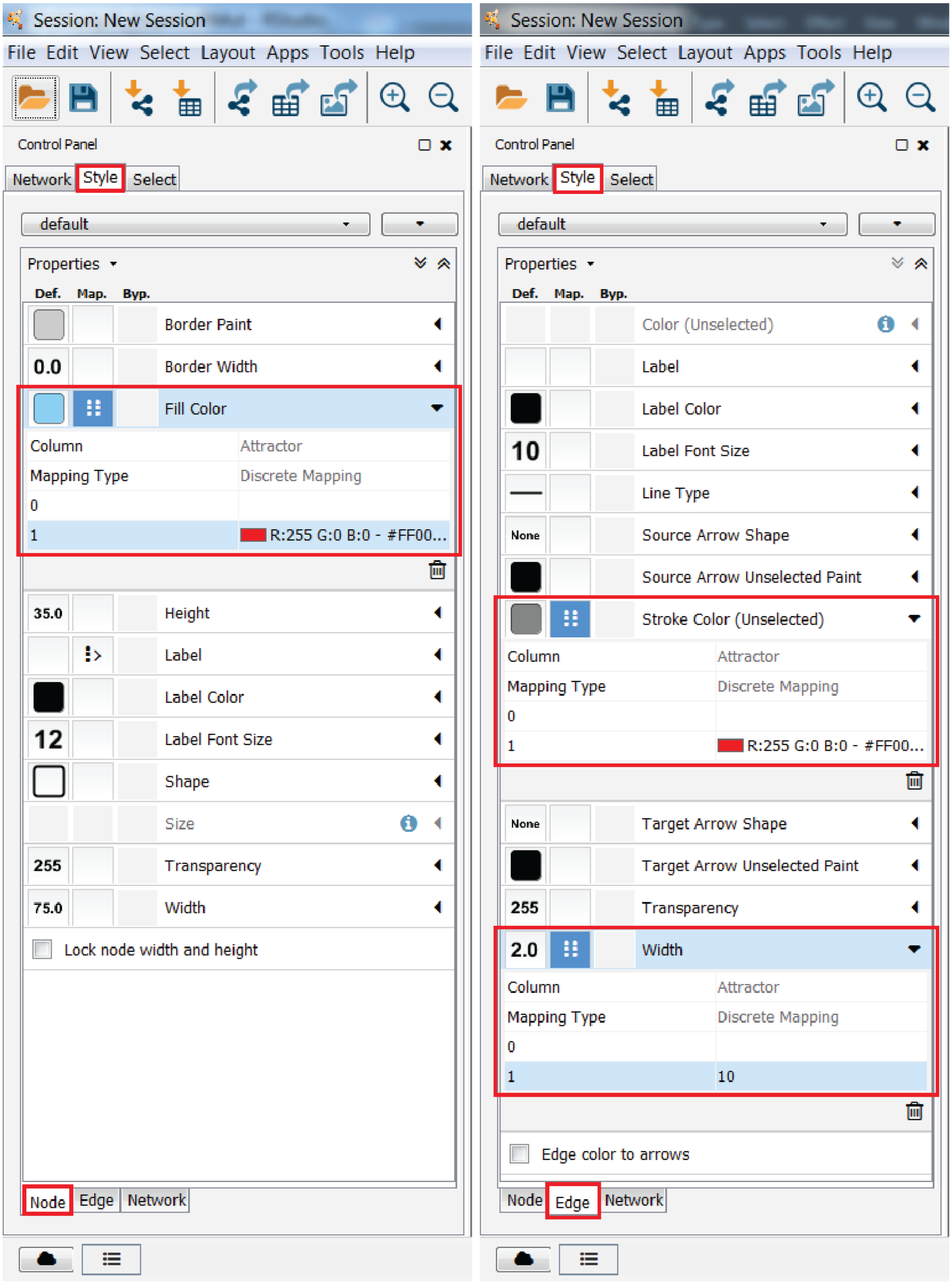

Figure 9: Nodes (a) and edges (b) style modification

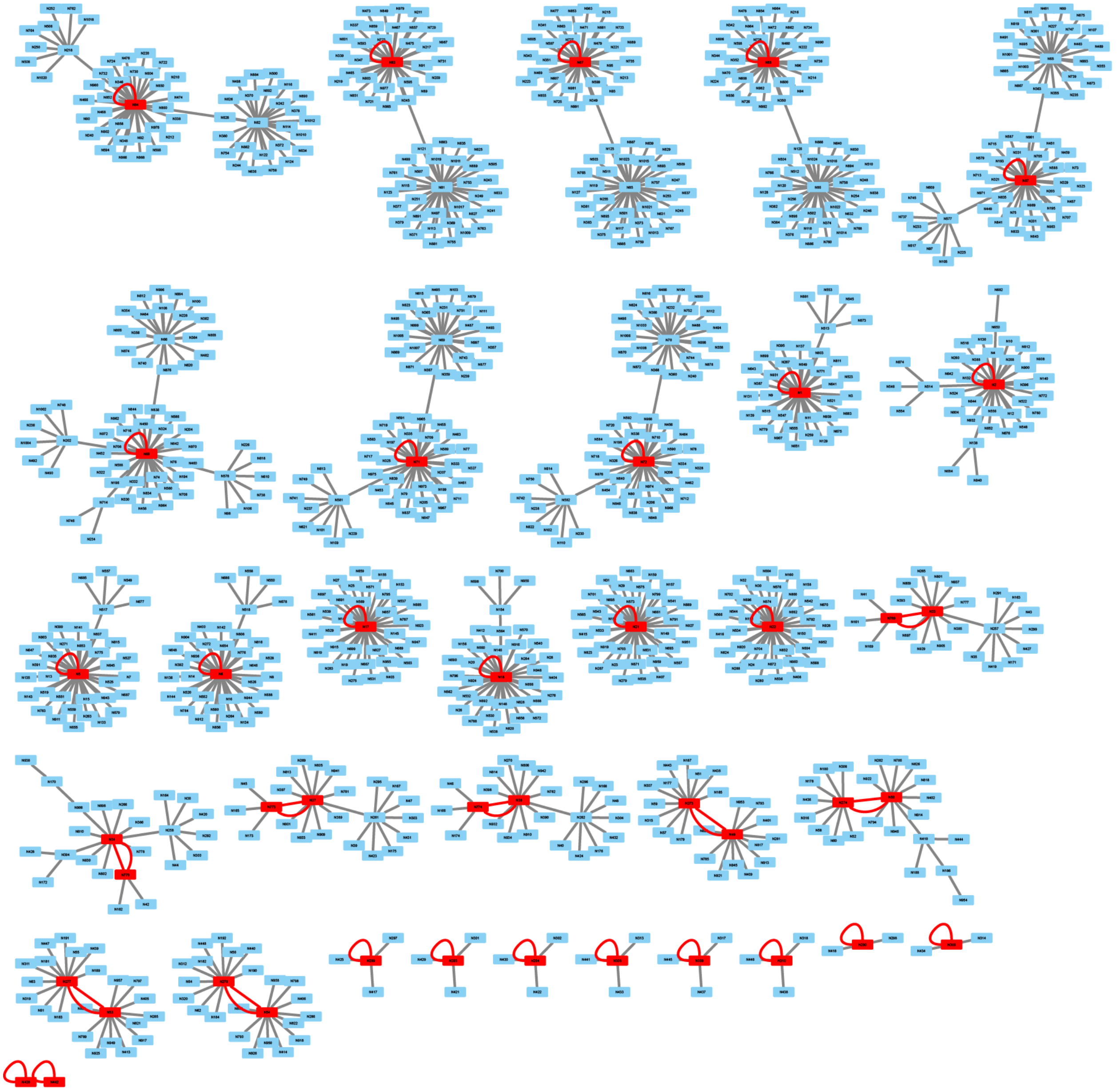

Figure 10: The transition network of AMRN

```
## 5      LFY     4        2        2    11        5        4        2
## 6      LUG     0        0        0     0        0        0        0
## 7       PI     1        1        0     6        0        3        3
## 8      SUP     0        0        0     2        2        0        0
## 9     TFL1     2        1        1     4        1        2        1
## 10     UFO     0        0        0     2        2        0        0
```

```r
print(amrn$edges)
```

```
##             EdgeID NuFBL NuPosFBL NuNegFBL NuFFL NuFFL_AB NuFFL_BC NuFFL_AC
## 1     AG (-1) AP1     3        1        2     1        0        1        0
## 2     AP1 (-1) AG     1        1        0     2        0        1        1
## 3     AP1 (1) LFY     3        1        2     2        1        1        0
## 4     AP3 (1) AP3     0        0        0     0        0        0        0
## 5      AP3 (1) PI     1        1        0     3        0        3        0
## 6   EMF1 (-1) AP1     0        0        0     2        1        0        1
## 7   EMF1 (-1) LFY     0        0        0     3        2        0        1
## 8   EMF1 (1) TFL1     0        0        0     2        1        0        1
## 9   LFY (-1) TFL1     2        1        1     2        1        1        0
## 10     LFY (1) AG     1        0        1     4        1        2        1
## 11    LFY (1) AP1     1        1        0     3        1        1        1
## 12    LFY (1) AP3     0        0        0     2        1        0        1
## 13     LFY (1) PI     0        0        0     2        1        0        1
## 14    LUG (-1) AG     0        0        0     0        0        0        0
## 15     PI (1) AP3     1        1        0     3        0        3        0
## 16      PI (1) PI     0        0        0     0        0        0        0
## 17   SUP (-1) AP3     0        0        0     2        1        0        1
## 18    SUP (-1) PI     0        0        0     2        1        0        1
## 19   TFL1 (-1) AG     1        0        1     2        0        1        1
## 20 TFL1 (-1) LFY     1        1        0     2        1        1        0
## 21    UFO (1) AP3     0        0        0     2        1        0        1
## 22     UFO (1) PI     0        0        0     2        1        0        1
```

```r
print(amrn$network)
```

```
##   NetworkID NuFBL NuPosFBL NuNegFBL NuFFL NuCoFFL NuInCoFFL
## 1      AMRN     6        4        2    15      10         5
```

In the above output, some abbreviations in the two nodes/edges data frames are explained as follows (refer to the literature [3-4] in the References section for more details):

- *NuFBL*: number of feedback loops involving the node/edge
- *NuPosFBL*, *NuNegFBL*: number of positive and negative feedback loops, respectively, involving the node/edge
- *NuFFL*: number of feed-forward loops involving the node/edge
- *NuFFL_A*, *NuFFL_B* and *NuFFL_C*: number of feed-forward loops with role A, B and C, respectively, involving the node
- *NuFFL_AB*, *NuFFL_BC* and *NuFFL_AC*: number of feed-forward loops with role AB, BC and AC, respectively, involving the edge

In the *network* data frame, *NuFBL*, *NuPosFBL*, *NuNegFBL*, *NuFFL*, *NuCoFFL* and *NuInCoFFL* denote total numbers of FBLs, positive/negative FBLs, FFLs and coherent/incoherent FFLs in the network, respectively.

4.2 Centrality measures computation

The *calCentrality* function calculates node-/edge-based centralities of a network such as Degree, In-/Out-Degree, Closeness, Betweenness, Stress, Eigenvector, Edge Degree and Edge Betweenness. An example is demonstrated as follows:

```r
data(amrn)

# calculate node-/edge-based centralities
amrn <- calCentrality(amrn)
print(amrn$nodes)
```

```
##    NodeID Degree In_Degree Out_Degree  Closeness Betweenness Stress
## 1      AG      5         4          1 0.01923077   5.5000000      6
## 2     AP1      5         3          2 0.02083333   8.3333333      9
## 3     AP3      7         5          2 0.01234568   0.0000000      0
## 4    EMF1      3         0          3 0.02564103   0.0000000      0
## 5     LFY      8         3          5 0.02222222  13.8333333     15
## 6     LUG      1         0          1 0.02083333   0.0000000      0
## 7      PI      7         5          2 0.01234568   0.0000000      0
## 8     SUP      2         0          2 0.01388889   0.0000000      0
## 9    TFL1      4         2          2 0.02083333   0.3333333      1
## 10    UFO      2         0          2 0.01388889   0.0000000      0
##     Eigenvector
## 1  1.962552e-01
## 2  3.688391e-01
## 3  8.780781e-49
## 4  6.569244e-01
## 5  4.969356e-01
## 6  1.044252e-01
## 7  8.780781e-49
## 8  1.756156e-48
## 9  3.688391e-01
## 10 1.756156e-48
```

```r
print(amrn$edges)
```

```
##            EdgeID Degree Betweenness
## 1    AG (-1) AP1     10   10.500000
## 2    AP1 (-1) AG     10    1.333333
## 3    AP1 (1) LFY     13   12.000000
## 4    AP3 (1) AP3     14    0.000000
## 5     AP3 (1) PI     14    1.000000
## 6  EMF1 (-1) AP1      8    1.333333
## 7  EMF1 (-1) LFY     11    3.333333
## 8  EMF1 (1) TFL1      7    1.333333
## 9  LFY (-1) TFL1     12    4.000000
## 10    LFY (1) AG     13    1.333333
## 11   LFY (1) AP1     13    1.500000
## 12   LFY (1) AP3     15    6.000000
## 13    LFY (1) PI     15    6.000000
## 14   LUG (-1) AG      6    6.000000
## 15    PI (1) AP3     14    1.000000
## 16     PI (1) PI     14    0.000000
## 17  SUP (-1) AP3      9    1.000000
```

```
## 18   SUP (-1) PI      9    1.000000
## 19  TFL1 (-1) AG      9    1.833333
## 20 TFL1 (-1) LFY     12    3.500000
## 21   UFO (1) AP3      9    1.000000
## 22    UFO (1) PI      9    1.000000
```

5 Export results

Via *output* function, all examined attributes of the networks and their nodes/edges will be exported to CSV files. The structure of these networks are also exported as Tab-separated values text files (.SIF extension). The following is an example R code for the output:

```r
data(amrn)

# generate all possible initial-states each containing 10 Boolean nodes
set1 <- generateStates(10, "all")

# generate all possible groups each containing a single node in the AMRN network
amrn <- generateGroups(amrn, "all", 1, 0)
```

```
## [1] "Number of possibly mutated groups:10"
```

```r
amrn <- calSensitivity(amrn, set1, "knockout")

# search feedback/feed-forward loops
amrn <- findFBLs(amrn, maxLength = 10)
```

```
## [1] "Number of found FBLs:6"
## [1] "Number of found positive FBLs:4"
## [1] "Number of found negative FBLs:2"
```

```r
amrn <- findFFLs(amrn)
```

```
## [1] "Number of found FFLs:15"
## [1] "Number of found coherent FFLs:10"
## [1] "Number of found incoherent FFLs:5"
```

```r
# calculate node-/edge-based centralities
amrn <- calCentrality(amrn)

# export all results to CSV files
output(amrn)
```

```
## [1] "All output files get created in the working directory:"
## [1] "D:/HCStore/Projects/R/RMut/vignettes"
```

6 Batch-mode analysis

The methods of dynamics and structure analysis described in the above sections (except the *findAttractors* function due to memory limitation) could also be applied to a set of networks, not limited to a single network. The RMut package provides the *createRBNs* function to generate a set of random networks using a generation model from among four models (refer to the literature in the References section for more details):

- Barabasi-Albert (BA) model [1]

- Erdos-Renyi (ER) variant model [2]
- Two shuffling models (Shuffle 1 and Shuffle 2) [3]

Here, we show two examples of generating a set of random networks and analyzing dynamics-related sensitivity and structural characteristic of those networks:

Example 1

```
# Example 1: generate random networks based on BA model #
#########################################################

# generate all possible initial-states each containing 10 Boolean nodes
set1 <- generateStates(10, "all")

# generate two random networks based on BA model
ba_rbns <- createRBNs("BA_RBN_", 2, "BA", 10, 17)

# for each random network, generate all possible groups each containing a single node
ba_rbns <- generateGroups(ba_rbns, "all", 1, 0)
```

```
## [1] "Number of possibly mutated groups:10"
## [1] "Number of possibly mutated groups:10"
```

```
# for each random network, calculate the sensitivity values of all nodes against "knockout" mutation
ba_rbns <- calSensitivity(ba_rbns, set1, "knockout")

# for each random network, calculate structural measures of all nodes/edges
ba_rbns <- findFBLs(ba_rbns, maxLength = 10)
```

```
## [1] "Number of found FBLs:5"
## [1] "Number of found positive FBLs:2"
## [1] "Number of found negative FBLs:3"
## [1] "Number of found FBLs:4"
## [1] "Number of found positive FBLs:2"
## [1] "Number of found negative FBLs:2"
```

```
ba_rbns <- findFFLs(ba_rbns)
```

```
## [1] "Number of found FFLs:4"
## [1] "Number of found coherent FFLs:0"
## [1] "Number of found incoherent FFLs:4"
## [1] "Number of found FFLs:6"
## [1] "Number of found coherent FFLs:4"
## [1] "Number of found incoherent FFLs:2"
```

```
ba_rbns <- calCentrality(ba_rbns)

print(ba_rbns)
```

```
## [[1]]
## $name
## [1] "BA_RBN_1"
##
## $nodes
##    NodeID NuFBL NuPosFBL NuNegFBL NuFFL NuFFL_A NuFFL_B NuFFL_C Degree
## 1       0     4        1        3     3       0       1       2     10
## 2       1     1        1        0     2       2       0       0      5
## 3       2     0        0        0     1       0       0       1      3
```

```
## 4       3    2        1        1     1       1       0       0      3
## 5       4    1        1        0     1       0       1       0      2
## 6       5    1        1        0     0       0       0       0      2
## 7       6    1        0        1     0       0       0       0      3
## 8       7    0        0        0     0       0       0       0      2
## 9       8    0        0        0     1       0       1       0      2
## 10      9    1        0        1     0       0       0       0      2
##    In_Degree Out_Degree  Closeness Betweenness Stress Eigenvector
## 1          6          4 0.02631579        32.0     36   0.4737116
## 2          1          4 0.06666667         8.0      8   0.5386850
## 3          2          1 0.01234568         4.5      7   0.0000000
## 4          1          2 0.02439024         6.0      6   0.3861732
## 5          1          1 0.02380952         0.0      0   0.2520567
## 6          1          1 0.04347826         0.0      0   0.2866283
## 7          1          2 0.02439024         2.5      5   0.2520567
## 8          2          0 0.01111111         0.0      0   0.0000000
## 9          1          1 0.02857143         0.0      0   0.2520567
## 10         1          1 0.02325581         0.0      0   0.2520567
##
## $edges
##      EdgeID NuFBL NuPosFBL NuNegFBL NuFFL NuFFL_AB NuFFL_BC NuFFL_AC
## 1  0 (-1) 3     2        1        1     0        0        0        0
## 2  0 (-1) 6     1        0        1     0        0        0        0
## 3  0 (-1) 9     1        0        1     0        0        0        0
## 4   0 (1) 2     0        0        0     1        0        1        0
## 5  1 (-1) 0     0        0        0     2        1        0        1
## 6  1 (-1) 5     1        1        0     0        0        0        0
## 7   1 (1) 2     0        0        0     1        0        0        1
## 8   1 (1) 8     0        0        0     1        1        0        0
## 9  2 (-1) 7     0        0        0     0        0        0        0
## 10 3 (-1) 4     1        1        0     1        1        0        0
## 11  3 (1) 0     1        0        1     1        0        0        1
## 12  4 (1) 0     1        1        0     1        0        1        0
## 13 5 (-1) 1     1        1        0     0        0        0        0
## 14 6 (-1) 7     0        0        0     0        0        0        0
## 15  6 (1) 0     1        0        1     0        0        0        0
## 16  8 (1) 0     0        0        0     1        0        1        0
## 17  9 (1) 0     1        0        1     0        0        0        0
##    Degree Betweenness
## 1      13        13.0
## 2      13         9.5
## 3      12         7.0
## 4      13         8.5
## 5      15        10.0
## 6       7         1.0
## 7       8         4.0
## 8       7         2.0
## 9       5         5.5
## 10      5         7.0
## 11     13         5.0
## 12     12         6.0
## 13      7         9.0
## 14      5         3.5
## 15     13         5.0
```

```
## 16     12         7.0
## 17     12         6.0
##
## $network
##   NetworkID NuFBL NuPosFBL NuNegFBL NuFFL NuCoFFL NuInCoFFL
## 1  BA_RBN_1     5        2        3     4       0         4
##
## $transitionNetwork
## [1] FALSE
##
## $Group_1
##    GroupID knockout_t1000_r1_macro knockout_t1000_r1_bitws
## 1        4                     1.0                    0.10
## 2        7                     1.0                    0.10
## 3        5                     1.0                    0.10
## 4        9                     0.0                    0.00
## 5        3                     0.0                    0.00
## 6        2                     0.0                    0.00
## 7        8                     0.5                    0.05
## 8        1                     0.0                    0.00
## 9        6                     0.0                    0.00
## 10       0                     0.0                    0.00
##
## attr(,"class")
## [1] "list"    "NetInfo"
##
## [[2]]
## $name
## [1] "BA_RBN_2"
##
## $nodes
##    NodeID NuFBL NuPosFBL NuNegFBL NuFFL NuFFL_A NuFFL_B NuFFL_C Degree
## 1       0     2        2        0     2       1       1       0      6
## 2       1     1        0        1     3       0       2       1      5
## 3       2     1        1        0     3       1       1       1      5
## 4       3     2        0        2     2       0       0       2      6
## 5       4     1        1        0     0       0       0       0      2
## 6       5     0        0        0     1       1       0       0      2
## 7       6     1        0        1     0       0       0       0      2
## 8       7     0        0        0     1       1       0       0      2
## 9       8     1        1        0     0       0       0       0      2
## 10      9     1        0        1     0       0       0       0      2
##    In_Degree Out_Degree  Closeness Betweenness Stress  Eigenvector
## 1          3          3 0.03030303        14.5     21 0.5085434716
## 2          4          1 0.01538462         6.5     11 0.0004888656
## 3          2          3 0.03125000        11.0     16 0.2901696807
## 4          4          2 0.01562500        17.0     23 0.0006476091
## 5          1          1 0.02564103         0.0      0 0.3835723105
## 6          0          2 0.03846154         0.0      0 0.6024349671
## 7          1          1 0.01515152         0.0      0 0.0004888656
## 8          0          2 0.01785714         0.0      0 0.0008578994
## 9          1          1 0.02631579         2.0      2 0.3835723105
## 10         1          1 0.01515152         2.0      2 0.0003690338
##
```

```
## $edges
##      EdgeID NuFBL NuPosFBL NuNegFBL NuFFL NuFFL_AB NuFFL_BC NuFFL_AC
## 1  0 (-1) 1     0        0        0     1        0        0        1
## 2  0 (-1) 2     1        1        0     2        1        1        0
## 3   0 (1) 4     1        1        0     0        0        0        0
## 4  1 (-1) 3     1        0        1     2        0        2        0
## 5   2 (1) 1     0        0        0     2        1        1        0
## 6   2 (1) 3     0        0        0     1        0        0        1
## 7   2 (1) 8     1        1        0     0        0        0        0
## 8  3 (-1) 9     1        0        1     0        0        0        0
## 9   3 (1) 6     1        0        1     0        0        0        0
## 10  4 (1) 0     1        1        0     0        0        0        0
## 11 5 (-1) 2     0        0        0     1        0        0        1
## 12  5 (1) 0     0        0        0     1        1        0        0
## 13 6 (-1) 3     1        0        1     0        0        0        0
## 14 7 (-1) 1     0        0        0     1        1        0        0
## 15  7 (1) 3     0        0        0     1        0        0        1
## 16 8 (-1) 0     1        1        0     0        0        0        0
## 17 9 (-1) 1     1        0        1     0        0        0        0
##    Degree Betweenness
## 1      11         8.0
## 2      11         9.5
## 3       8         4.0
## 4      11         9.5
## 5      10         1.5
## 6      11        10.5
## 7       7         6.0
## 8       8        11.0
## 9       8         9.0
## 10      8         7.0
## 11      7         5.5
## 12      8         2.5
## 13      8         3.0
## 14      7         1.0
## 15      8         3.0
## 16      8         9.0
## 17      7         5.0
##
## $network
##   NetworkID NuFBL NuPosFBL NuNegFBL NuFFL NuCoFFL NuInCoFFL
## 1  BA_RBN_2     4        2        2     6       4         2
##
## $transitionNetwork
## [1] FALSE
##
## $Group_1
##    GroupID knockout_t1000_r1_macro knockout_t1000_r1_bitws
## 1        7              0.50000000             0.075507812
## 2        0              0.05664062             0.009882813
## 3        2              1.00000000             0.280742187
## 4        6              0.50000000             0.167070312
## 5        9              1.00000000             0.154335937
## 6        3              0.05273438             0.013085938
## 7        5              0.50000000             0.061210938
```

```
## 8        1              0.50000000            0.067070312
## 9        8              0.50000000            0.151445312
## 10       4              0.50000000            0.024414062
##
## attr(,"class")
## [1] "list"    "NetInfo"
```

```
output(ba_rbns)
```

```
## [1] "All output files get created in the working directory:"
## [1] "D:/HCStore/Projects/R/RMut/vignettes"
```

Example 2

```
# Example 2: generate random networks based on "Shuffle 2" model #
##################################################################

data(amrn)

# generate all possible initial-states each containing 10 Boolean nodes
set1 <- generateStates(10, "all")

# generate two random networks based on "Shuffle 2" model
amrn_rbns <- createRBNs("AMRN_RBN_", 2, "shuffle 2", referedNetwork = amrn)

# for each random network, generate all possible groups each containing a single edge
amrn_rbns <- generateGroups(amrn_rbns, "all", 0, 1)
```

```
## [1] "Number of possibly mutated groups:22"
## [1] "Number of possibly mutated groups:22"
```

```
# for each random network, calculate the sensitivity values of all edges against "remove" mutation
amrn_rbns <- calSensitivity(amrn_rbns, set1, "edge removal")

# for each random network, calculate structural measures of all nodes/edges
amrn_rbns <- findFBLs(amrn_rbns, maxLength = 10)
```

```
## [1] "Number of found FBLs:10"
## [1] "Number of found positive FBLs:6"
## [1] "Number of found negative FBLs:4"
## [1] "Number of found FBLs:9"
## [1] "Number of found positive FBLs:4"
## [1] "Number of found negative FBLs:5"
```

```
amrn_rbns <- findFFLs(amrn_rbns)
```

```
## [1] "Number of found FFLs:15"
## [1] "Number of found coherent FFLs:12"
## [1] "Number of found incoherent FFLs:3"
## [1] "Number of found FFLs:21"
## [1] "Number of found coherent FFLs:9"
## [1] "Number of found incoherent FFLs:12"
```

```
amrn_rbns <- calCentrality(amrn_rbns)

print(amrn_rbns)
```

```
## [[1]]
```

```
## $name
## [1] "AMRN_RBN_1"
##
## $nodes
##    NodeID NuFBL NuPosFBL NuNegFBL NuFFL NuFFL_A NuFFL_B NuFFL_C Degree
## 1      AG     4        0        4     5       0       2       3      5
## 2     AP1     3        2        1     4       1       3       0      5
## 3     AP3     9        6        3     7       1       1       5      7
## 4    EMF1     0        0        0     2       2       0       0      3
## 5     LFY     8        5        3    10       8       2       0      8
## 6     LUG     0        0        0     0       0       0       0      1
## 7      PI     6        2        4     8       0       3       5      7
## 8     SUP     0        0        0     1       1       0       0      2
## 9    TFL1     4        2        2     4       0       3       1      4
## 10    UFO     0        0        0     1       1       0       0      2
##    In_Degree Out_Degree  Closeness Betweenness Stress Eigenvector
## 1          4          1 0.01851852    0.500000      1   0.1036192
## 2          3          2 0.02083333    3.333333      5   0.2753881
## 3          5          2 0.02083333   14.166667     17   0.3654706
## 4          0          3 0.02500000    0.000000      0   0.2817502
## 5          3          5 0.02222222   15.833333     18   0.5571382
## 6          0          1 0.02439024    0.000000      0   0.2618515
## 7          5          2 0.02000000    6.833333     10   0.2204693
## 8          0          2 0.02439024    0.000000      0   0.3011996
## 9          2          2 0.02040816    1.333333      2   0.2204693
## 10         0          2 0.02500000    0.000000      0   0.3654706
##
## $edges
##           EdgeID NuFBL NuPosFBL NuNegFBL NuFFL NuFFL_AB NuFFL_BC NuFFL_AC
## 1     AG (-1) PI     4        0        4     2        0        2        0
## 2  AP1 (-1) TFL1     2        1        1     2        1        1        0
## 3    AP1 (1) AP3     1        1        0     3        0        2        1
## 4    AP3 (1) LFY     8        5        3     1        1        0        0
## 5     AP3 (1) PI     1        1        0     2        0        1        1
## 6  EMF1 (-1) AP1     0        0        0     0        0        0        0
## 7   EMF1 (-1) PI     0        0        0     2        1        0        1
## 8    EMF1 (1) AG     0        0        0     2        1        0        1
## 9  LFY (-1) TFL1     2        1        1     3        2        0        1
## 10    LFY (1) AG     1        0        1     2        1        0        1
## 11   LFY (1) AP1     3        2        1     2        2        0        0
## 12   LFY (1) AP3     1        1        0     2        1        0        1
## 13    LFY (1) PI     1        1        0     5        2        2        1
## 14  LUG (-1) LFY     0        0        0     0        0        0        0
## 15     PI (1) AG     1        0        1     2        0        2        0
## 16    PI (1) AP3     5        2        3     1        0        1        0
## 17  SUP (-1) AP1     0        0        0     1        1        0        0
## 18  SUP (-1) AP3     0        0        0     1        0        0        1
## 19  TFL1 (-1) AG     2        0        2     1        0        1        0
## 20 TFL1 (-1) AP3     2        2        0     2        0        2        0
## 21   UFO (1) LFY     0        0        0     1        1        0        0
## 22    UFO (1) PI     0        0        0     1        0        0        1
##    Degree Betweenness
## 1      12    5.500000
## 2       9    4.333333
```

```
## 3      12    4.000000
## 4      15   14.833333
## 5      14    4.333333
## 6       8    3.000000
## 7      10    2.000000
## 8       8    1.000000
## 9      12    6.000000
## 10     13    3.333333
## 11     13    7.000000
## 12     15    2.500000
## 13     15    2.000000
## 14      9    6.000000
## 15     12    2.333333
## 16     14    9.500000
## 17      7    2.333333
## 18      9    3.666667
## 19      9    2.833333
## 20     11    3.500000
## 21     10    4.000000
## 22      9    2.000000
##
## $network
##    NetworkID NuFBL NuPosFBL NuNegFBL NuFFL NuCoFFL NuInCoFFL
## 1 AMRN_RBN_1    10        6        4    15      12         3
##
## $transitionNetwork
## [1] FALSE
##
## $Group_1
##          GroupID edgeremoval_t1000_r1_macro edgeremoval_t1000_r1_bitws
## 1     UFO (1) PI                       0.00                      0.000
## 2    AP1 (1) AP3                       0.00                      0.000
## 3  AP1 (-1) TFL1                       0.00                      0.000
## 4   EMF1 (-1) PI                       0.00                      0.000
## 5    LFY (1) AP3                       0.25                      0.025
## 6     AG (-1) PI                       0.00                      0.000
## 7     LFY (1) AG                       0.00                      0.000
## 8   SUP (-1) AP3                       0.00                      0.000
## 9  EMF1 (-1) AP1                       0.00                      0.000
## 10 LFY (-1) TFL1                       0.00                      0.000
## 11   AP3 (1) LFY                       0.50                      0.050
## 12   UFO (1) LFY                       0.00                      0.000
## 13    PI (1) AP3                       0.00                      0.000
## 14   EMF1 (1) AG                       0.00                      0.000
## 15    AP3 (1) PI                       0.00                      0.000
## 16  SUP (-1) AP1                       0.00                      0.000
## 17     PI (1) AG                       0.00                      0.000
## 18  LUG (-1) LFY                       0.00                      0.000
## 19   LFY (1) AP1                       0.00                      0.000
## 20    LFY (1) PI                       0.50                      0.100
## 21  TFL1 (-1) AG                       0.00                      0.000
## 22 TFL1 (-1) AP3                       0.00                      0.000
##
## attr(,"class")
```

```
## [1] "list"    "NetInfo"
##
## [[2]]
## $name
## [1] "AMRN_RBN_2"
##
## $nodes
##    NodeID NuFBL NuPosFBL NuNegFBL NuFFL NuFFL_A NuFFL_B NuFFL_C Degree
## 1      AG     4        0        4     7       0       2       5      5
## 2     AP1     7        4        3     6       1       2       3      5
## 3     AP3     7        2        5    10       1       4       5      7
## 4    EMF1     0        0        0     4       4       0       0      3
## 5     LFY     6        4        2    13       8       4       1      8
## 6     LUG     0        0        0     0       0       0       0      1
## 7      PI     7        3        4     7       0       4       3      7
## 8     SUP     0        0        0     1       1       0       0      2
## 9    TFL1     2        1        1     5       2       2       1      4
## 10    UFO     0        0        0     1       1       0       0      2
##    In_Degree Out_Degree  Closeness Betweenness Stress Eigenvector
## 1          4          1 0.01818182         1.0      2  0.08774418
## 2          3          2 0.02083333        12.5     14  0.35564013
## 3          5          2 0.01960784         8.5     10  0.16754969
## 4          0          3 0.02564103         0.0      0  0.57574021
## 5          3          5 0.02222222        12.0     15  0.51155400
## 6          0          1 0.02222222         0.0      0  0.12159890
## 7          5          2 0.02040816        17.0     19  0.23219614
## 8          0          2 0.02325581         0.0      0  0.20934308
## 9          2          2 0.01960784         1.0      2  0.13369497
## 10         0          2 0.02500000         0.0      0  0.33791073
##
## $edges
##           EdgeID NuFBL NuPosFBL NuNegFBL NuFFL NuFFL_AB NuFFL_BC NuFFL_AC
## 1    AG (-1) AP3     4        0        4     2        0        2        0
## 2   AP1 (-1) AP3     1        0        1     2        0        1        1
## 3    AP1 (1) LFY     6        4        2     2        1        1        0
## 4     AP3 (1) AG     1        0        1     3        0        2        1
## 5     AP3 (1) PI     6        2        4     3        1        2        0
## 6  EMF1 (-1) AP1     0        0        0     2        1        0        1
## 7  EMF1 (-1) LFY     0        0        0     3        2        0        1
## 8    EMF1 (1) PI     0        0        0     2        1        0        1
## 9  LFY (-1) TFL1     2        1        1     3        2        1        0
## 10    LFY (1) AG     1        0        1     2        1        0        1
## 11   LFY (1) AP1     1        1        0     3        1        1        1
## 12   LFY (1) AP3     1        1        0     4        2        1        1
## 13    LFY (1) PI     1        1        0     4        2        1        1
## 14   LUG (-1) PI     0        0        0     0        0        0        0
## 15     PI (1) AG     1        0        1     2        0        2        0
## 16    PI (1) AP1     6        3        3     2        0        2        0
## 17  SUP (-1) AP3     0        0        0     1        1        0        0
## 18   SUP (-1) PI     0        0        0     1        0        0        1
## 19  TFL1 (-1) AG     1        0        1     3        1        1        1
## 20 TFL1 (-1) AP3     1        1        0     3        1        1        1
## 21   UFO (1) LFY     0        0        0     1        1        0        0
## 22  UFO (1) TFL1     0        0        0     1        0        0        1
```

```
##    Degree Betweenness
## 1      12         6.0
## 2      12         3.5
## 3      13        14.0
## 4      12         2.0
## 5      14        11.5
## 6       8         1.5
## 7      11         3.0
## 8      10         1.5
## 9      12         8.0
## 10     13         2.5
## 11     13         2.0
## 12     15         2.0
## 13     15         2.5
## 14      8         6.0
## 15     12         4.0
## 16     12        18.0
## 17      9         1.5
## 18      9         4.5
## 19      9         1.5
## 20     11         4.5
## 21     10         4.0
## 22      6         2.0
##
## $network
##    NetworkID NuFBL NuPosFBL NuNegFBL NuFFL NuCoFFL NuInCoFFL
## 1 AMRN_RBN_2     9        4        5    21       9        12
##
## $transitionNetwork
## [1] FALSE
##
## $Group_1
##          GroupID edgeremoval_t1000_r1_macro edgeremoval_t1000_r1_bitws
## 1      PI (1) AG                 0.00000000               0.0000000000
## 2  TFL1 (-1) AP3                 0.01171875               0.0015625000
## 3   SUP (-1) AP3                 0.00000000               0.0000000000
## 4   AP1 (-1) AP3                 0.00000000               0.0000000000
## 5    AG (-1) AP3                 0.00000000               0.0000000000
## 6     AP3 (1) PI                 0.24609375               0.0369791667
## 7  LFY (-1) TFL1                 0.01171875               0.0015625000
## 8   TFL1 (-1) AG                 0.00000000               0.0000000000
## 9    EMF1 (1) PI                 0.00000000               0.0000000000
## 10   AP1 (1) LFY                 0.01171875               0.0015625000
## 11   LUG (-1) PI                 0.00000000               0.0000000000
## 12    LFY (1) PI                 0.01171875               0.0011718750
## 13   UFO (1) LFY                 0.00000000               0.0000000000
## 14   LFY (1) AP1                 0.00000000               0.0000000000
## 15  UFO (1) TFL1                 0.00000000               0.0000000000
## 16 EMF1 (-1) AP1                 0.01171875               0.0015625000
## 17   SUP (-1) PI                 0.00781250               0.0009765625
## 18    AP3 (1) AG                 0.00000000               0.0000000000
## 19 EMF1 (-1) LFY                 0.00000000               0.0000000000
## 20    LFY (1) AG                 0.06250000               0.0070312500
## 21   LFY (1) AP3                 0.03125000               0.0070312500
```

```
## 22    PI (1) AP1                 0.00000000              0.0000000000
##
## attr(,"class")
## [1] "list"    "NetInfo"
```

```
output(amrn_rbns)
```

```
## [1] "All output files get created in the working directory:"
## [1] "D:/HCStore/Projects/R/RMut/vignettes"
```

7 References

1. Barabasi A-L, Albert R (1999) Emergence of Scaling in Random Networks. Science 286: 509-512. doi: 10.1126/science.286.5439.509
2. Le D-H, Kwon Y-K (2011) NetDS: A Cytoscape plugin to analyze the robustness of dynamics and feedforward/feedback loop structures of biological networks. Bioinformatics.
3. Trinh H-C, Le D-H, Kwon Y-K (2014) PANET: A GPU-Based Tool for Fast Parallel Analysis of Robustness Dynamics and Feed-Forward/Feedback Loop Structures in Large-Scale Biological Networks. PLoS ONE 9: e103010.
4. Koschutzki D, Schwobbermeyer H, Schreiber F (2007) Ranking of network elements based on functional substructures. Journal of Theoretical Biology 248: 471-479.
